# Enhancing photovoltaic parameter estimation: integration of non-linear hunting and reinforcement learning strategies with golden jackal optimizer

**DOI:** 10.1038/s41598-024-52670-8

**Published:** 2024-02-02

**Authors:** Chappani Sankaran Sundar Ganesh, Chandrasekaran Kumar, Manoharan Premkumar, Bizuwork Derebew

**Affiliations:** 1 Department of Electrical and Electronics Engineering, Karpagam College of Engineering, Coimbatore, Tamil Nadu 641032 India; 2 Department of Electrical and Electronics Engineering, Dayananda Sagar College of Engineering, Bengaluru, Karnataka 560078 India; 3 Department of Statistics, College of Natural and Computational Science, Mizan-Tepi University, Tepi Bushira, Ethiopia

**Keywords:** Engineering, Mathematics and computing

## Abstract

The advancement of Photovoltaic (PV) systems hinges on the precise optimization of their parameters. Among the numerous optimization techniques, the effectiveness of each often rests on their inherent parameters. This research introduces a new methodology, the Reinforcement Learning-based Golden Jackal Optimizer (RL-GJO). This approach uniquely combines reinforcement learning with the Golden Jackal Optimizer to enhance its efficiency and adaptability in handling various optimization problems. Furthermore, the research incorporates an advanced non-linear hunting strategy to optimize the algorithm’s performance. The proposed algorithm is first validated using 29 CEC2017 benchmark test functions and five engineering-constrained design problems. Secondly, rigorous testing on PV parameter estimation benchmark datasets, including the single-diode model, double-diode model, three-diode model, and a representative PV module, was carried out to highlight the superiority of RL-GJO. The results were compelling: the root mean square error values achieved by RL-GJO were markedly lower than those of the original algorithm and other prevalent optimization methods. The synergy between reinforcement learning and GJO in this approach facilitates faster convergence and improved solution quality. This integration not only improves the performance metrics but also ensures a more efficient optimization process, especially in complex PV scenarios. With an average Freidman’s rank test values of 1.564 for numerical and engineering design problems and 1.742 for parameter estimation problems, the proposed RL-GJO is performing better than the original GJO and other peers. The proposed RL-GJO stands out as a reliable tool for PV parameter estimation. By seamlessly combining reinforcement learning with the golden jackal optimizer, it sets a new benchmark in PV optimization, indicating a promising avenue for future research and applications.

## Introduction

The global challenge today revolves around the environmental impact of electricity generation from traditional energy sources. When unfiltered, these sources release harmful greenhouse gases, primarily nitrogen oxides and sulfur oxides. These pollutants are major culprits behind air contamination, accelerated climate shifts, and the broader issue of global warming^1^. Given these pressing concerns, there has been a marked shift towards seeking alternative energy sources as replacements for traditional ones. The emphasis is on devising technologies that are cost-effective, sustainable, and, above all, eco-friendly. Addressing the rising concerns of carbon dioxide emissions and the urgent need for carbon neutrality is essential in our efforts to safeguard the environment. In this context, renewable energy sources have gained significant traction as viable solutions to these challenges. Among the myriad of renewable energy options, such as wind, tidal, and solar power, solar energy stands out as one of the most promising avenues. Its appeal lies in its cleanliness, ubiquity, and the fact that it’s an endless resource^2,3^.

Solar photovoltaic (PV) systems have emerged as a leading contender in this search. The sun, as an inexhaustible energy reservoir, offers a sustainable solution across various global regions. Solar energy’s potential is harnessed by converting it into electrical energy, a process facilitated by photovoltaic (PV) systems^4^. Within the realm of PV systems, there exists a variety of models designed to optimize this energy conversion. Notably, the Single Diode Model (SDM) and Double Diode Model (DDM) are frequently employed due to their effectiveness and reliability^5,6^. PV systems harnessing solar energy are versatile in their deployment, ranging from expansive solar farms to compact rooftop installations. Furthermore, solar technology stands out for its minimal environmental footprint. However, it is worth noting that these PV systems, often stationed outdoors, are subjected to the rigours of the external environment. Exposure to varying weather conditions and other environmental factors can lead to their degradation over time. As such, there’s a pressing need to monitor, control, and predict the behaviour of these PV systems to ensure they operate at peak energy efficiency. An essential aspect of this monitoring process revolves around the model parameters of the PV systems. These parameters play a decisive role in influencing the overall performance of the systems. Ensuring their accuracy and reliability is crucial, not just for the system’s quality control but also for performance estimation and maximum power point tracking. Proper parameter management can significantly enhance the efficiency and longevity of PV systems, making them even more valuable in our search for sustainable energy solutions^7,8^.

Accurate and efficient modelling of solar cells and PV modules remains a pivotal challenge in PV systems. This complexity arises from the inherent non-linear nature of solar cells and the often-incomplete availability of their parameters. For a comprehensive analysis and evaluation of PV system behaviour, it is imperative to establish a precise model for simulation purposes. Such a model aids in crafting high-performance controllers and optimizing PV system functionality. Over time, various mathematical constructs have been introduced to capture PV characteristics under diverse operational conditions. A prevalent choice is the diode-based model^9^. The SDM stands out for its simplicity, encompassing five parameters. In contrast, the DDM offers a more detailed representation with seven parameters^10^. For an even more nuanced understanding, the Three-Diode Model (TDM) incorporates factors like leakage current coefficients, grain boundaries, and carrier recombination, encompassing nine parameters^11^. More recently, the multi-diode model has emerged, tailored to encapsulate various PV technologies. This model integrates multiple parallel diodes, with its parameters varying based on the number of diodes. Another innovative approach is the multi-dimension diode PV model, characterized as a structured network of numerous series and parallel diodes, renowned for its precision. It is evident that as models incorporate more parameters, they gain in accuracy but also in complexity. The choice of model often hinges on the specific PV application and strikes a balance between precision and simplicity. Ultimately, the more adeptly we discern the model’s unknown parameters, the closer we get to optimizing the PV system’s performance and control^12,13^.

In the vast expanse of literature, the methodologies for identifying the parameters of PV systems can be broadly categorized into three primary approaches: analytical, deterministic, and metaheuristics. The analytical method is typically straightforward and rapid. It operates on certain assumptions to simplify the process. However, this simplicity often comes at the cost of precision, leading to potential inaccuracies in the derived solutions. Methods like the Lagrangian equation^14^ and Newton–Raphson^15^ fall under the deterministic category. These techniques initiate their search for the optimal solution from a predetermined starting point, leveraging gradient information. A significant challenge with deterministic methods is their sensitivity to these initial points. The choice of these starting points can profoundly influence the outcome, and there is a definite risk of these methods getting ensnared in local optima, especially when identifying parameters for systems with complex, non-linear characteristics. Given the non-linear nature of PV systems, metaheuristic methods, inspired by swarm intelligence and natural phenomena, have gained prominence. These algorithms stand out for their simplicity, flexibility, and ability to sidestep local optima. They don’t impose stringent requirements on PV models, making them versatile tools for parameter identification^16^.

Deterministic methodologies can be bifurcated into two primary categories: analytical and iterative techniques. Analytical strategies leverage data from PV datasheets or their I–V characteristic curves to frame the parameter estimation challenge. Notable analytical methods encompass the reduced space search^17^, Lambert-W function^18^, etc. However, these methods, while comprehensive, can be intricate and time-intensive. They rely on solving non-linear equations, and as the number of unidentified parameters in the PV model grows, so does the computational complexity. To simplify these equations, certain model adjustments might be made, potentially leading to less accurate results. Iterative techniques, on the other hand, rely on a repetitive process, often involving trial and error. Notable examples include the Newton–Raphson^19^, Gauss–Seidel^20^, and least squares^21^ methods. For these to be effective, the system’s equations must exhibit specific properties like convexity, continuity, and differentiability. Moreover, the choice of initial values is crucial, as incorrect selections can lead to suboptimal solutions. Given the limitations of deterministic methods, the research community has gravitated towards metaheuristic strategies. Here, the unknown parameters of the PV model are discerned by addressing an optimization challenge. By crafting an apt objective function, these parameters are refined to minimize the function’s value.

According to the existing body of research, a range of heuristic methods have been effectively employed for the extraction of PV parameters^22^. Several of these methods are enumerated below: genetic algorithm^23^, simulated annealing^24^, artificial bee colony algorithm^25^, artificial bee swarm optimization^26^, harmony search^27^, particle swarm optimization^28^, sunflower optimization^29^, Moth-Flame Optimizer (MFO)^30^, salp swarming algorithm^31^, Grey Wolf Optimizer (GWO)^32^, whale optimizer^33^, multi-verse optimizer^34^, flower-pollinating optimization ^35^, Conscious Neighbourhood-Based Crow Search Algorithm (CNCSA)^36^, slime mould algorithm^37^, Jaya optimization^38^, water cycle optimization^39^, Marine Predator Algorithm (MPA)^40^, wind driven optimization^41^, equilibrium optimizer^42^, quantum-based avian navigation optimizer (QANO)^43^, Gradient-Based Optimizer (GBO)^44^, reptile search algorithm^45^, bird mating optimizer^46^, mine blast algorithm^47^, Runge–Kutta optimizer^48^, cat swarm optimization^49^, teaching learning based optimization ^50^, starling murmuration optimizer^51^, northern goshawk optimizer^52^, atom search optimizer^53^, Harris hawk optimizer^54^, resistance–capacitance optimizer^11^, beluga whale optimizer^55^, growth optimizer^56^, hunter-prey optimizer^57^, MFO with stagnation finding and replacing strategy^58^, Newton–Raphson-based optimizer^59^, etc. While heuristic methods have demonstrated superior accuracy and performance compared to deterministic methods, many heuristic approaches exhibit a significant requirement for iterations to achieve convergence or yield inconsistent outcomes upon repetition. In order to minimize the number of iterations and achieve reliable outcomes, several enhanced techniques have been suggested. These methods include chaos particle swarm optimisation, mutated particle swarm optimization, guaranteed convergence particle swarm optimisation, improved opposition-based whale optimization, etc. The utilization of the Root Mean Squared Error (RMSE) as the basis for the objective function necessitates the acquisition of experimental data pertaining to PV cells/modules, which manufacturers typically do not furnish. Hence, certain authors have chosen to establish an alternative objective function by utilizing the information presented in the PV datasheet and have then determined the values of its unidentified parameters through the use of heuristic approaches. Several instances can be provided: The optimization techniques discussed in the literature include shuffle-frog leaped optimization, bacterial foraged optimization, differential evolution, etc. The No-Free Lunch (NFL) thesis posits that an optimizer is incapable of attaining the optimal global solution for all optimization problems^60^. Consequently, researchers employ several optimization techniques in order to ascertain and estimate the unidentified parameters of the photovoltaic model.

Recently, a new optimization technique termed the Golden Jackal Optimization (GJO) algorithm, has been introduced^61^. This method offers a fresh approach to tackling real-world engineering challenges. Drawing inspiration from the cooperative hunting tactics of golden jackals, the GJO algorithm incorporates three fundamental stages: prey detection, encirclement, and attack. While the GJO algorithm exhibits rapid convergence, it tends to plateau in local minima when faced with intricate optimization challenges. This limitation becomes more noticeable in the context of PV parameter identification, especially as the count of unidentified parameters intensifies. To enhance the GJO algorithm’s efficacy in pinpointing the global optimal solution, this paper introduces an advanced variant termed Reinforced-Learning-integrated GJO (RL-GJO). Reinforcement learning (RL) operates as a reward-centric machine learning method where an agent independently navigates the best strategy through interactions with its environment^62^. In this RL paradigm, after taking an action in a specific state, the agent receives a feedback reward from the environment. This feedback aids in refining subsequent actions, mirroring human learning processes^63^. Various studies have harnessed the power of reinforcement learning to gain control over actions and address a range of optimization challenges. For example, Ref.^64^ presented an RL-driven deterministic policy gradient to design a controller for pinpointing the best trajectory path. Ref.^65^ put forth an enhanced RL technique to chart the optimal course for multi-agent systems, leveraging greedy actions to perceive and gauge the environment, supplemented by artificial neural networks and a kernel smoothing approach. Meanwhile, Ref.^66^ employed a deep RL framework to achieve adaptive control for autonomous underwater vehicles, yielding results that surpassed existing benchmark algorithms.

The study aims to address the critical challenge of optimizing PV parameters, which is essential for enhancing the efficiency and output of PV systems. The accurate estimation and optimization of these parameters directly impact the performance and reliability of solar energy systems. As the global demand for renewable energy sources grows, improving PV system performance becomes increasingly vital for sustainable energy development. This study contributes to this field by introducing an innovative optimization technique that promises to make PV systems more efficient and cost-effective.

The choice of employing the GJO^61^ in this research is grounded in several key considerations: (i) GJO is a novel metaheuristic optimization algorithm inspired by the foraging behaviour of golden jackals. This nature-inspired approach is known for its efficiency in exploring and exploiting the solution space; (ii) GJO is further enhanced with RL techniques to improve its adaptability and decision-making capabilities. This integration allows the algorithm to learn from its environment and make more informed choices, leading to more accurate and efficient optimization; (iii) The characteristics of GJO, particularly its robustness and ability to handle complex, nonlinear problems, make it well-suited for PV parameter optimization. PV systems often involve intricate interactions between multiple parameters, and GJO’s strategic exploration and exploitation tactics are effective in navigating these complexities; (iv) The combination of GJO and reinforcement learning in optimizing PV parameters offers several advantages: (1) The adaptive nature of GJO, enhanced by learning strategies, leads to more accurate parameter estimation; (2) The algorithm shows improved convergence speed, which is crucial in time-sensitive applications; and (3) The proposed method demonstrates robust performance across a range of scenarios, maintaining its effectiveness under varying conditions. Q-Learning (QL) is a form of RL methodology that functions without relying on an explicit representation of the environment^67,68^. The utilization of the Q-learning and GJO algorithm integration has been implemented to augment the search capability of the GJO algorithm, which the improvements in RL have facilitated. The non-linear hunting strategy is also employed for its ability to enhance population diversity by utilizing an exponentially declining vector across successive rounds. The proposed strategy incorporates an unfavourable approach for constructing the differential vector while simultaneously taking into account the inclusion of randomly selected jackals. This is done to mitigate the issue of local stagnation, which frequently arises when the male jackal rapidly converges towards a singular point inside the search space. Q-learning and non-linear hunting strategies were chosen based on their direct applicability in resolving various inherent challenges associated with GJO. These challenges include the tendency to converge towards local optima, sensitivity to parameters, and the lack of a balanced exploration–exploitation trade-off. Competing paradigms have the potential to introduce unnecessary complexity or may not align as effectively with the specific dynamics and objectives of GJO. This research has made notable advancements in several key areas:

- Implementation of a non-linear hunting strategy to enhance population diversity in the algorithm.
- Integration of Q-learning to optimize the exploration and exploitation capabilities of the GJO algorithm.
- Comprehensive performance evaluation using 29 CEC2017 benchmark test functions and five engineering design problems.
- Extensive validation through diverse case studies involving various PV models.
- Detailed comparison and analysis of the RL-GJO’s performance against other state-of-the-art algorithms

The structure of the paper is as follows. Section “Mathematical models” discusses the mathematical modelling and objective function of the considered problem. Section “Proposed algorithm” comprehensively discusses the formulation procedures of the proposed RL-GJO algorithm. It also briefs the basic concepts of the GJO algorithm. Section “Results and discussions” discusses the results obtained from different case studies, including CEC2017 benchmark test functions and PV models. Section “Conclusions” concludes the paper with future scope.

## Mathematical models

In the subsequent section, we delve into the details of the problem related to extracting parameters for PV systems. This section begins by outlining the foundational framework of the PV parameter extraction problem. Following this, this study introduces three primary equivalent circuit models for PV cells and modules: the SDM, DDM, and TDM. Each of these models offers a unique perspective and approach to representing the behaviour and characteristics of PV systems. To provide a comprehensive understanding, this study further explores the mathematical underpinnings that govern these models. This section also presents the objective function that forms the root of the optimization challenge. This function is pivotal as it defines the criteria based on which the optimization process evaluates potential solutions.

### SDM of the PV cell/module

**Figure 1:**
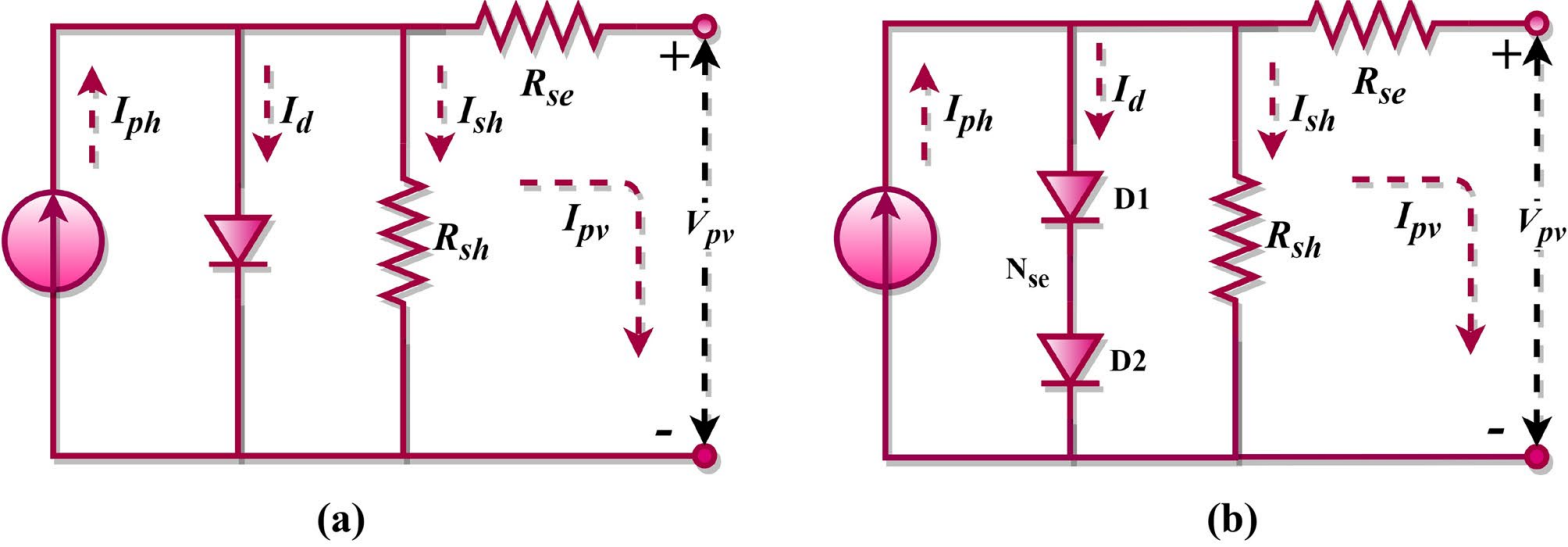
Single diode model; (**a**) PV cell, (**b**) PV module.

The fundamental unit of a PV system, often referred to as its core component, is the PV cell. This cell plays a pivotal role in transforming solar energy into direct current electrical power. To capture the non-linear behaviour of PV cells, the single-diode equivalent circuit is commonly employed. This model represents a PV cell using various components: a current source, a diode, a series resistor, and a shunt resistor, as depicted in Fig. 1a. The PV cell’s output current is described in Eq. (1)^69,70^. Detailed expressions for the diode current, junction thermal voltage, shunt resistance current, and the PV cell’s output current are provided in Eqs. (2)–(5), respectively.

1 $${I}_{pv, c}={I}_{ph}-{I}_{d}-{I}_{sh}$$

2 $${I}_{d}={I}_{sd}\left[exp\left(\frac{{V}_{pv,c}+{R}_{se}{I}_{pv,c}}{a{V}_{t}}\right)-1\right]$$

3 $${V}_{t}=\frac{kT}{q}$$

4 $${I}_{sh}=\frac{{V}_{pv,c}+{R}_{se}{I}_{pv,c}}{{R}_{sh}}$$

5 $${I}_{pv, c}={I}_{ph}-{I}_{sd}\left[exp\left(\frac{{V}_{pv,c}+{R}_{se}{I}_{pv,c}}{a{V}_{t}}\right)-1\right]-\frac{{V}_{pv,c}+{R}_{se}{I}_{pv,c}}{{R}_{sh}}$$

When multiple PV cells are interconnected in series or parallel configurations, they form what is known as PV modules. Figure 1b showcases the single-diode equivalent circuit representation for such a PV module, with its output current defined by Eq. (6) For the context of this research, it is suggested that the PV module is an assembly of cells linked in a series arrangement. For both SDM (cell) and SDM (module) configurations, five essential parameters, such as ${I}_{ph}$, ${I}_{sd}$, $a$, ${R}_{sh}$, and ${R}_{se}$ must be determined.

6 $${I}_{pv, m}={I}_{ph}-{I}_{sd}\left[exp\left(\frac{{V}_{pv, m}+{{N}_{se}{R}_{se}I}_{pv, m}}{a{V}_{t}{N}_{se}}\right)-1\right]-\frac{{V}_{pv,m}+{{N}_{se}{R}_{s}I}_{pv, m}}{{R}_{sh}{N}_{se}}$$

where ${I}_{pv, c}$ and ${I}_{pv, m}$ denotes the output current of the PV cell and module, respectively, ${I}_{ph}$ denotes the photo-generated current, ${I}_{sd}$ signifies the diode saturation current, $a$ denotes the diode ideality factor, and the ohmic resistances are represented as ${R}_{sh}$ and ${R}_{se}$, ${V}_{pv, c}$ and ${V}_{pv, m}$ denotes the output voltage of the PV cell and module, respectively, ${N}_{se}$ denotes the number of cells connected in series, $k$ denotes the Boltzmann constant, $q$ denotes the electron charge, and $T$ denotes the cell temperature.

### DDM of the PV cell/module

**Figure 2:**
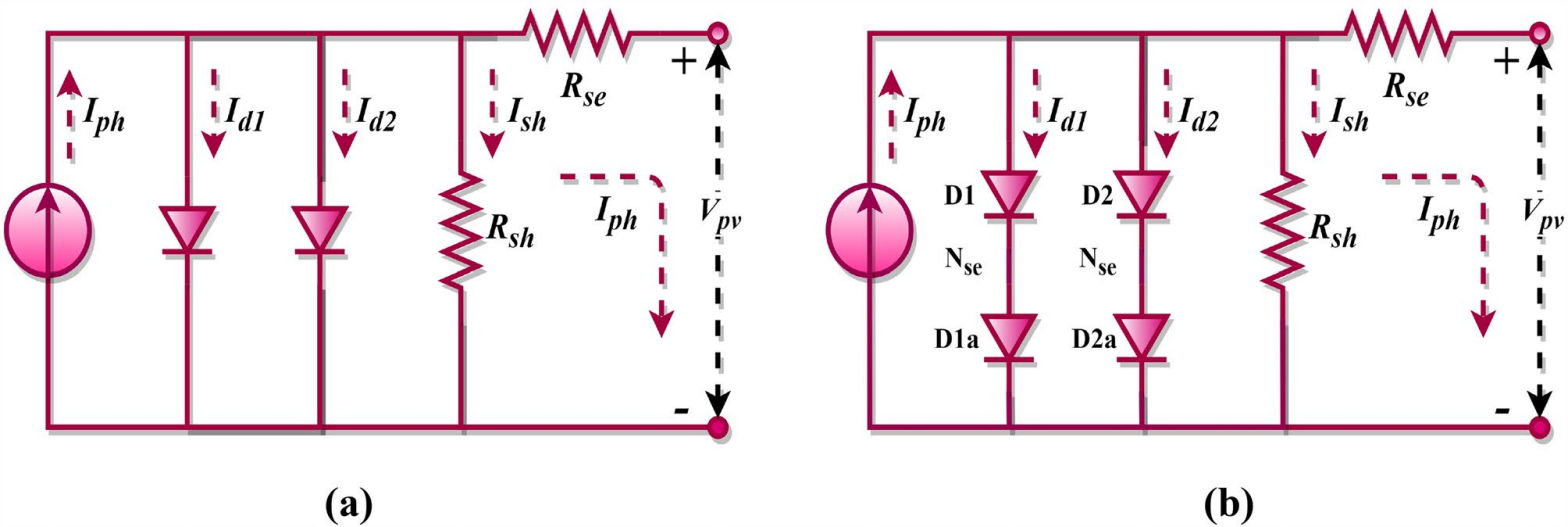
Double diode model; (**a**) PV cell, (**b**) PV module.

Beyond the SDM, the DDM incorporates recombination losses occurring within the depletion region. The double-diode equivalent circuit is frequently chosen to depict the non-linear attributes of both PV cells and modules. This model, which essentially augments the SDM with an extra diode, is illustrated in Fig. 2a. The output current for this PV cell configuration is represented by Eq. (7)^71,72^. Detailed formulations for the first diode’s current, the second diode’s current, the shunt resistance current, and the overall output current of the PV cell are provided in Eqs. (8)–(10), respectively.

7 $${I}_{pv, c}={I}_{ph}-{I}_{d1}-{I}_{d2}-{I}_{sh}$$

8 $${I}_{d1}={I}_{sd1}\left[exp\left(\frac{{V}_{pv,c}+{R}_{se}{I}_{pv,c}}{{a}_{1}{V}_{t}}\right)-1\right]$$

9 $${I}_{d2}={I}_{sd2}\left[exp\left(\frac{{V}_{pv,c}+{R}_{se}{I}_{pv,c}}{{a}_{2}{V}_{t}}\right)-1\right]$$

10 $${I}_{pv, c}={I}_{ph}-{I}_{sd1}\left[exp\left(\frac{{V}_{pv,c}+{R}_{se}{I}_{pv,c}}{{a}_{1}{V}_{t}}\right)-1\right]-{I}_{sd2}\left[exp\left(\frac{{V}_{pv,c}+{R}_{se}{I}_{pv,c}}{{a}_{2}{V}_{t}}\right)-1\right]-\frac{{V}_{pv,c}+{R}_{se}{I}_{pv,c}}{{R}_{sh}}$$

The double-diode equivalent circuit representation for a PV module is presented in Fig. 2b, with its corresponding output current defined by Eq. (11). In this context, the underlying assumption is that the PV module is composed of cells interconnected in a series fashion. Consequently, for DDM (cell) and DDM (module) configurations, a total of seven key parameters, such as ${I}_{ph}$, ${I}_{sd1}$, ${I}_{sd2}$, ${a}_{1}$, ${a}_{2}$, ${R}_{sh}$, and ${R}_{se}$ must be determined.

11 $${I}_{pv, m}={I}_{ph}-{I}_{sd1}\left[exp\left(\frac{{V}_{pv, m}+{{N}_{se}{R}_{se}I}_{pv, m}}{{a}_{1}{V}_{t}{N}_{se}}\right)-1\right]-{I}_{sd2}\left[exp\left(\frac{{V}_{pv, m}+{{N}_{se}{R}_{se}I}_{pv, m}}{{a}_{2}{V}_{t}{N}_{se}}\right)-1\right]-\frac{{V}_{pv,m}+{{N}_{se}{R}_{s}I}_{pv, m}}{{R}_{sh}{N}_{se}}$$

where ${I}_{sd1}$ and ${I}_{sd2}$ signifies the saturation current of diode 1 and diode 2, respectively, and $a$ denotes the ideality factor of diode 1 and diode 2, respectively.

### TDM of the PV cell/module

**Figure 3:**
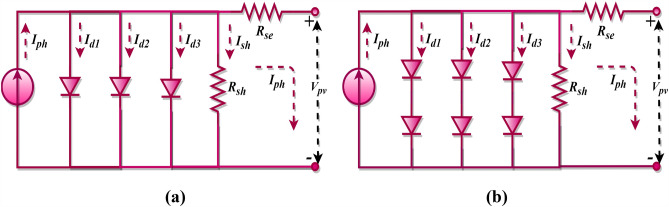
Three diode model; (**a**) PV cell, (**b**) PV module.

In continuation with the previous models, the TDM further refines the representation by considering additional complexities in the PV cell behaviour. The TDM accounts for various intrinsic characteristics and losses that might not be captured by the single or double-diode models. A three-diode equivalent circuit is frequently adopted to represent the intricate non-linear attributes of PV cells and modules. This model, which essentially extends the DDM by integrating a third diode, is depicted in Fig. 3a. The output current for this PV cell configuration is represented by Eq. (12)^73,74^. The formulations for the overall output current of the PV cell and the third diode’s current are provided in Eqs. (13) and (14), respectively.

12 $${I}_{pv, c}={I}_{ph}-{I}_{d1}-{I}_{d2}-{I}_{d3}-{I}_{sh}$$

13 $${I}_{d3}={I}_{sd2}\left[exp\left(\frac{{V}_{pv,c}+{R}_{se}{I}_{pv,c}}{{a}_{3}{V}_{t}}\right)-1\right]$$

14 $$\begin{aligned}{I}_{pv, c} & ={I}_{ph}-{I}_{sd1}\left[exp\left(\frac{{V}_{pv,c}+{R}_{se}{I}_{pv,c}}{{a}_{1}{V}_{t}}\right)-1\right]-{I}_{sd2}\left[exp\left(\frac{{V}_{pv,c}+{R}_{se}{I}_{pv,c}}{{a}_{2}{V}_{t}}\right)-1\right]\\ &\quad-{I}_{sd3}\left[exp\left(\frac{{V}_{pv,c}+{R}_{se}{I}_{pv,c}}{{a}_{3}{V}_{t}}\right)-1\right]-\frac{{V}_{pv,c}+{R}_{se}{I}_{pv,c}}{{R}_{sh}}\end{aligned}$$

The three-diode equivalent circuit representation for a PV module is presented in Fig. 3b, with its corresponding output current defined by Eq. (15). As with the previous models, the underlying assumption remains that the PV module is composed of cells interconnected in a series arrangement. Consequently, for the TDM (cell) and TDM (module) configurations, a total of nine essential parameters, such as ${I}_{ph}$, ${I}_{sd1}$, ${I}_{sd2}$, ${I}_{sd3}$, ${a}_{1}$, ${a}_{2}$, ${a}_{3}$, ${R}_{sh}$, and ${R}_{se}$ must be determined.

15 $$\begin{aligned}{I}_{pv, m}&={I}_{ph}-{I}_{sd1}\left[exp\left(\frac{{V}_{pv, m}+{{N}_{se}{R}_{se}I}_{pv, m}}{{a}_{1}{V}_{t}{N}_{se}}\right)-1\right]-{I}_{sd2}\left[exp\left(\frac{{V}_{pv, m}+{{N}_{se}{R}_{se}I}_{pv, m}}{{a}_{2}{V}_{t}{N}_{se}}\right)-1\right]\\&\quad-{I}_{sd3}\left[exp\left(\frac{{V}_{pv, m}+{{N}_{se}{R}_{se}I}_{pv, m}}{{a}_{3}{V}_{t}{N}_{se}}\right)-1\right]-\frac{{V}_{pv,m}+{{N}_{se}{R}_{s}I}_{pv, m}}{{R}_{sh}{N}_{se}}\end{aligned}$$

### Objective function

This study focuses on extracting parameters for PV systems. To set the stage, this study first introduces the models for PV cells and modules using the SDM, DDM, and TDM equivalent circuits. Following this foundational understanding, this section delves into the optimization phase, employing the proposed RL-GJO algorithm to pinpoint the optimal parameters. This optimization leverages real-world current and voltage data from PV cells and modules gathered under specific irradiance and temperature conditions. Utilizing this measured data, parameter extraction is executed in alignment with a predefined objective function. The functions and decision variables for the SDM, DDM, and TDM models, which highlight the variance between the projected and actual current, are articulated in Eq. (16). The unknown parameters, i.e., the parameters to be optimized for each model, represented in Eq. (17). This study’s primary goal, represented by the objective function defined as the RMSE, is to minimize the disparity between the estimated values and the real-world data, as detailed in Eq. (18).

16 $$f\left(x\right)=\left\{\begin{aligned} & {I}_{ph}-{I}_{sd}\left[exp\left(\frac{{V}_{pv,c}+{R}_{se}{I}_{pv,c}}{a{V}_{t}}\right)-1\right]-\frac{{V}_{pv,c}+{R}_{se}{I}_{pv,c}}{{R}_{sh}}-{I}_{pv, c}\\ & {I}_{ph}-{I}_{sd}\left[exp\left(\frac{{V}_{pv, m}+{{N}_{se}{R}_{se}I}_{pv, m}}{a{V}_{t}{N}_{se}}\right)-1\right]-\frac{{V}_{pv,m}+{{N}_{se}{R}_{s}I}_{pv, m}}{{R}_{sh}{N}_{se}}-{I}_{pv, m}\\ & {I}_{ph}-{I}_{sd1}\left[exp\left(\frac{{V}_{pv,c}+{R}_{se}{I}_{pv,c}}{{a}_{1}{V}_{t}}\right)-1\right]-{I}_{sd2}\left[exp\left(\frac{{V}_{pv,c}+{R}_{se}{I}_{pv,c}}{{a}_{2}{V}_{t}}\right)-1\right]-\frac{{V}_{pv,c}+{R}_{se}{I}_{pv,c}}{{R}_{sh}}-{I}_{pv, c}\\ & {I}_{ph}-{I}_{sd1}\left[exp\left(\frac{{V}_{pv, m}+{{N}_{se}{R}_{se}I}_{pv, m}}{{a}_{1}{V}_{t}{N}_{se}}\right)-1\right]\\ & \quad -{I}_{sd2}\left[exp\left(\frac{{V}_{pv, m}+{{N}_{se}{R}_{se}I}_{pv, m}}{{a}_{2}{V}_{t}{N}_{se}}\right)-1\right]-\frac{{V}_{pv,m}+{{N}_{se}{R}_{s}I}_{pv, m}}{{R}_{sh}{N}_{se}}-{I}_{pv, m}\\ & {I}_{ph}-{I}_{sd1}\left[exp\left(\frac{{V}_{pv,c}+{R}_{se}{I}_{pv,c}}{{a}_{1}{V}_{t}}\right)-1\right]-{I}_{sd2}\left[exp\left(\frac{{V}_{pv,c}+{R}_{se}{I}_{pv,c}}{{a}_{2}{V}_{t}}\right)-1\right]\\ & \quad -{I}_{sd3}\left[exp\left(\frac{{V}_{pv,c}+{R}_{se}{I}_{pv,c}}{{a}_{3}{V}_{t}}\right)-1\right]-\frac{{V}_{pv,c}+{R}_{se}{I}_{pv,c}}{{R}_{sh}}-{I}_{pv, c}\\ & {I}_{ph}-{I}_{sd1}\left[exp\left(\frac{{V}_{pv, m}+{{N}_{se}{R}_{se}I}_{pv, m}}{{a}_{1}{V}_{t}{N}_{se}}\right)-1\right]-{I}_{sd2}\left[exp\left(\frac{{V}_{pv, m}+{{N}_{se}{R}_{se}I}_{pv, m}}{{a}_{2}{V}_{t}{N}_{se}}\right)-1\right]\\ & \quad-{I}_{sd3}\left[exp\left(\frac{{V}_{pv, m}+{{N}_{se}{R}_{se}I}_{pv, m}}{{a}_{3}{V}_{t}{N}_{se}}\right)-1\right]-\frac{{V}_{pv,m}+{{N}_{se}{R}_{s}I}_{pv, m}}{{R}_{sh}{N}_{se}}-{I}_{pv, m}\end{aligned}\right.$$

17 $$x=\left\{\begin{array}{c}{x}_{SDM,c}={I}_{ph},{I}_{sd},a,{R}_{se},{R}_{sh}\\ {x}_{SDM,m}={I}_{ph},{I}_{o},\alpha ,{R}_{s},{R}_{sh}\\ {x}_{DDM,c}={I}_{ph},{I}_{sd1},{I}_{sd2},{a}_{1},{a}_{2},{R}_{se},{R}_{sh}\\ {x}_{DDM,m}={I}_{ph},{I}_{sd1},{I}_{sd2},{a}_{1},{a}_{2},{R}_{se},{R}_{sh}\\ {x}_{TDM,c}={I}_{ph},{I}_{sd1},{I}_{sd2},{I}_{sd3},{a}_{1},{a}_{2},{a}_{3},{R}_{se},{R}_{sh}\\ {x}_{TDM,m}={I}_{ph},{I}_{sd1},{I}_{sd2},{I}_{sd3},{a}_{1},{a}_{2},{a}_{3},{R}_{se},{R}_{sh}\end{array}\right.$$

18 $$RMSE = \sqrt {\frac{1}{N}\mathop \sum \limits_{k = 1}^{N} f_{k} \left( x \right)^{2} } = \sqrt {\frac{1}{N}\mathop \sum \limits_{k = 1}^{N} \left( {I_{e} - I_{m} } \right)^{2} }$$

where $N$ denotes the number of experimental data, ${I}_{e}$ denotes the estimated output current of the PV cell/module, ${I}_{m}$ denotes the measured output current of the PV cell/module, $x$ denotes the control vector (problem dimension), ${f}_{k}\left(x\right)$ represents the function articulating the variance between the measured and estimated current of the $k$th sample, and $RMSE$ denotes the objective function.

## Proposed algorithm

This section provides a concise overview of the fundamental principles underlying the GJO algorithm. This paper provides a concise overview of the Q-learning mechanism and non-linear hunting strategy. Subsequently, a full analysis is presented on the formulation of the proposed RL-GJO method.

### Original golden jackal optimizer

The Golden Jackal Optimization (GJO) is an algorithm inspired by the collective behaviour of golden jackals during their hunting activities. This algorithm draws its motivation from the intricate and strategic hunting actions exhibited by pairs of golden jackals, as referenced in the source^61^. The hunting process of the golden jackals can be broken down into several critical phases: (i) Searching and Approaching the Target: In this initial phase, the jackals actively search for their prey. Once they spot a potential target, they move towards it, closing the distance between them and the prey, (ii) Encircling and Provoking the Prey: Once close enough, the jackals work together to surround the prey. They then engage in actions that provoke or agitate the prey, causing it to become increasingly defensive and stationary. The primary aim during this phase is to ensure that the prey is immobilized and cannot escape, and (iii) Launching the Attack: After successfully immobilizing the prey, the jackals proceed to attack. This is the final and decisive phase, where the jackals work in tandem to overpower and capture their prey. Furthermore, this section delves into the mathematical modelling of the GJO algorithm. This section provides a comprehensive discussion of the mathematical principles and formulas that underpin the GJO algorithm, offering insights into its computational mechanics and efficiency.

Like many metaheuristic algorithms, the GJO begins with a random distribution of the population across a chosen search domain. The formula for this initial distribution is given by:

19 $${X}_{init}=lb+random\times \left(ub-lb\right)$$

where ${X}_{init}$ represents the starting random population, $ub$ and $lb$ are the upper and lower boundaries of the decision variables, respectively, and $random$ is a random value ranging between [0,1]. This initialization forms the foundational Prey matrix. In this matrix, the positions of the first and second jackals are represented. The matrix is structured as follows:

20 $$Prey=\left[\begin{array}{cccc}{X}_{\mathrm{1,1}}& {X}_{\mathrm{1,2}}& \cdots & {X}_{1,d}\\ {X}_{\mathrm{2,1}}& {X}_{\mathrm{2,1}}& \cdots & {X}_{2,d}\\ :& :& :& :\\ {X}_{n,1}& {X}_{n,2}& \cdots & {X}_{n,d}\end{array}\right]$$

where $n$ signifies the total population (or prey), $d$ indicates the dimension of the problem, and the specific solution’s variables are termed the prey’s position. For optimization purposes, an objective function calculates the value for each member of the population. A separate matrix aggregates these values for all members:

21 $$S=\left[\begin{array}{c}f\left({X}_{\mathrm{1,1}};{X}_{\mathrm{1,2}};\cdots ;{X}_{1,d}\right)\\ f\left({X}_{\mathrm{2,1}};{X}_{\mathrm{2,1}};\cdots ;{X}_{2,d}\right)\\ :\\ f\left({X}_{n,1};{X}_{n,2};\cdots ;{X}_{n,d}\right)\end{array}\right]$$

where $S$ is the matrix that holds the fitness values for each population member, and $f$ is the function that determines fitness. The jackal with the highest fitness is designated as the male, while the one with the second-highest fitness is the female. Together, they assume the relevant positions in the prey matrix. The jackal possesses a remarkable skill set that allows them to identify and pursue their quarry, although there are instances when their intended prey manages to elude capture and makes a successful escape. In such situations, the jackals exhibit patience by pausing their pursuit and seeking out alternative sources of sustenance. During the hunt, it is typically the male jackal who takes the lead, with the female jackal following closely behind.

22 $${X}_{Mm}={X}_{M}\left(t\right)-E.\left|{X}_{M}\left(t\right)-r.Prey\left(t\right)\right|$$

23 $${X}_{FMm}={X}_{FM}\left(t\right)-E.\left|{X}_{FM}\left(t\right)-r.Prey\left(t\right)\right|$$

where ${X}_{M}\left(t\right)$ signifies the current position of the male jackal, ${X}_{FM}\left(t\right)$ represents the current position of the female jackal, ${X}_{Mm}$ denotes the updated position of the male jackal, ${X}_{FMm}$ represents the updated position of the female jackal, $Prey\left(t\right)$ signifies the position of the prey, $E$ signifies the evading energy, and $r$ denotes the random number distribution obtained by the Levy flight mechanism. The evade energy is calculated as follows.

24 $$E={E}_{0}\times {E}_{1}$$

25 $${E}_{0}=2\times {r}_{1}-1$$

26 $${E}_{1}={c}_{1}\times \left(1-\left(it/{T}_{max}\right)\right)$$

where ${E}_{1}$ signifies the falling energy, ${E}_{0}$ signifies the initial energy, ${r}_{1}$ signifies the uniform random number in the range of 0 to 1, the value of the constant ${c}_{1}=1.5$, $it$ denotes the current iteration, and ${T}_{max}$ represents the maximum iterations. The position of the jackal is updated using Eq. (27).

27 $$X\left(t+1\right)=\frac{{X}_{Mm}\left(t\right)+{X}_{FMm}\left(t\right)}{2}$$

In scenarios where jackals are pursuing their prey, the prey’s capacity for evasion diminishes, leading to the jackals, working as a pair, encircling the initially located prey. This strategy involves surrounding, attacking, and ultimately capturing the prey. The process of a male and female jackal hunting in tandem is mathematically depicted in Eqs. (28) and (29).

28 $${X}_{Mm}={X}_{M}\left(t\right)-E.\left|r.{X}_{M}\left(t\right)-Prey\left(t\right)\right|$$

29 $${X}_{FMm}={X}_{FM}\left(t\right)-E.\left|r.{X}_{FM}\left(t\right)-Prey\left(t\right)\right|$$

As per previous discussions, it is possible to determine all the parameters found in Eqs. (28), (29). Finally, the positions of the jackals are updated by Eq. (27). The term $r$ helps to prevent the local optimum trap. The ecological problems typically arise in the jackals’ pursuit tracks, impeding their ability to move swiftly and appropriately in the direction of their prey. Exploration and exploitation are balanced by the term $E$. The prey experiences a sharp decrease in energy when moving evasively. The value of ${E}_{0}$ fluctuates arbitrarily from − 1 to 1 in each cycle. The prey is weakened when the ${E}_{0}$ decreases from 0 to − 1; conversely, the prey is strengthened when the ${E}_{0}$ climbs from 0 to 1. The jackal pair searches different areas for prey to investigate when $|E|\ge 1$ and strikes and takes advantage of the victim when $\left|E\right|<1$. The pseudocode of the original algorithm is shown in Algorithm 1.

**Algorithm 1:**
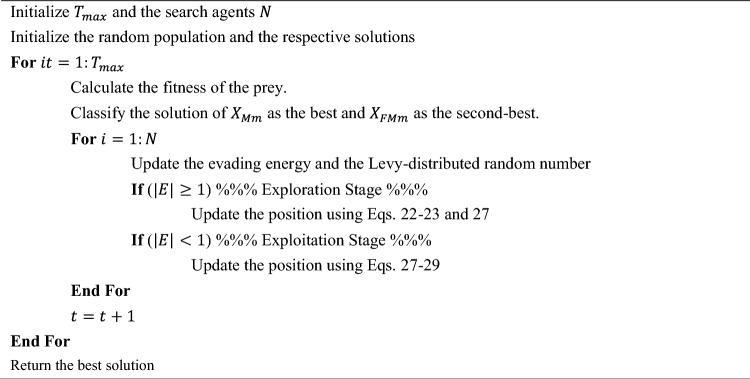
Pseudocode of the original GJO.

### Reinforcement learning-integrated golden jackal optimizer

#### Motivation

The hunting behaviour of golden jackals inspires the GJO algorithm. The algorithm uses both exploration and exploitation mechanisms to find the optimal solution. The primary entities in GJO are the male and female Jackals, which represent the best and second-best solutions, respectively. While it introduces novel mechanisms for exploration and exploitation, it can have limitations that affect its performance. The following are some reasons why GJO might perform poorly and the rationale for improving its performance: (i) One of the common challenges with optimization algorithms is maintaining diversity in the population. If the algorithm converges too quickly to a particular region of the solution space, it might get stuck in local optima and miss out on better solutions elsewhere. GJO, in its basic form, might suffer from premature convergence, especially if the male and female Jackals dominate the search direction for all agents. (ii) The movement of agents in GJO is determined by deterministic rules based on the positions of the Male and Female Jackals. Such deterministic behaviour can limit the algorithm’s ability to explore the solution space thoroughly. The algorithm might repeatedly explore the same regions of the solution space, leading to redundant evaluations and missed opportunities. (iii) Many modern optimization algorithms incorporate adaptive mechanisms that allow the algorithm to adjust its parameters or behaviour based on the current state of the search. GJO, in its basic form, lacks such adaptive mechanisms. Without adaptability, the algorithm might not perform well across a wide range of optimization problems with varying characteristics. To ensure that GJO remains competitive, it is essential to address its limitations and enhance its performance. By improving GJO, we can ensure that it performs well across a broader range of optimization problems, making it a more versatile tool for researchers and practitioners. Enhancements can lead to faster convergence and fewer function evaluations, making the algorithm more efficient and reducing computational costs. By integrating novel mechanisms like Q-learning and non-linear hunting schemes, it is possible to push the boundaries of what GJO can achieve and contribute to the advancement of the field.

#### Q-learning mechanism

Q-learning is a model-free RL algorithm used to find the optimal action-selection policy for a given finite Markov decision process^75,76^. It works by learning an action-value function and thereby gives a way to act optimally. The idea is to learn a policy that tells an agent what action to take under what circumstances. It does not require a model of the environment (hence “model-free”), and it can handle problems with stochastic transitions and rewards. In this study, the “agent” is a jackal, the “environment” is the optimization landscape, and the “actions” are the directions in which a jackal can move. The Q-value or the quality of an action taken in a state is represented by $Q(s,a)$, where $s$ is the current state and $a$ is the action taken. The Q-values are updated using Eq. (30).

30 $$Q\left({s}_{t},{a}_{t}\right)\leftarrow \left(1-\mathrm{\alpha }\right)\times Q\left({s}_{t},{a}_{t}\right)+\mathrm{\alpha }\times \left({r}_{t}+\upgamma \times \underset{a}{{\text{max}}}Q\left({s}_{t+1},a\right)\right)$$

$Q\left({s}_{t},{a}_{t}\right)$ is the current Q-value, which represents the expected utility of taking action ${a}_{t}$ (action at time $t$) in state ${s}_{t}$ (the state at time $t$). Q-values are essentially estimates of how good it is to take a certain action in a given state. $\left(1-\alpha \right)$ determines to what extent the newly acquired information will override the old information ($\alpha \mathrm{ is the learning rate}$). A factor of $\left(1-\mathrm{\alpha }\right)$ is multiplied by the current Q-value, indicating that part of the old value is retained. $\mathrm{\alpha }\times \left({r}_{t}+\upgamma \times \underset{a}{{\text{max}}}Q\left({s}_{t+1},a\right)\right)$ represents the learning update. ${r}_{t}$ is the reward received after taking action ${a}_{t}$ in state ${s}_{t}$. It represents the immediate gain from that action. $\upgamma$ is the discount factor, ranging between 0 and 1. It determines the importance of future rewards—a high gamma means long-term rewards are considered more heavily. $\underset{a}{{\text{max}}}Q\left({s}_{t+1},a\right)$ is the maximum Q-value for the next state ${s}_{t+1}$ over all possible actions. It represents the best estimated future reward from the next state. Equation (30) updates the Q-value by blending the old value with new information. The new information is a combination of the immediate reward and the estimated future rewards. This balance allows the algorithm to learn effectively, considering both its current and potential future states.

#### Non-linear hunting scheme

The non-linear hunting scheme aims to improve population diversity and prevent premature convergence^77^. This is achieved by (i) Using the worst solution to form a differential vector which pushes the population away from the worst areas of the search space, (ii) Randomly selecting jackals for updates to prevent dominance by a few good solutions and ensuring diversity, and (iii) Introducing non-linearity to prevent solutions from converging too quickly to a single point in the search space. The new position of a jackal using the non-linear hunting scheme is given by Eq. (31).

31 $${X}_{new}={X}_{current}+{\beta }^{\left(\frac{it}{{T}_{max}}\right)}\times \left({X}_{current}-{X}_{worst}\right)+\delta \times \left({X}_{random}-{X}_{current}\right)$$

where ${X}_{current}$ is the current position of the jackal, ${X}_{worst}$ is the position of the worst jackal, ${X}_{random}$ is the position of a randomly selected jackal, $\beta$ is an exponential decay factor, and $\delta$ is a random number between 0 and 1.

#### Integrating Q-learning and non-linear hunting with GJO

Traditional GJO relies on deterministic rules for exploration and exploitation based on the positions of the Male and Female Jackals. Q-learning, on the other hand, provides a mechanism for adaptive decision-making. It allows the algorithm to “learn” from its past experiences and make decisions that are more likely to lead to better solutions in the future. One of the challenges in optimization algorithms is balancing exploration and exploitation. Q-learning inherently addresses this challenge. By using an $\varepsilon$-greedy policy, it ensures that the algorithm occasionally tries random actions (exploration) while mostly acting on its learned knowledge (exploitation). The adaptive nature of Q-learning can help the algorithm avoid getting stuck in local minima. If a particular action consistently leads to poor solutions, its Q-value will decrease, making it less likely to be chosen in the future. By learning from past experiences and making more informed decisions, the algorithm can potentially converge to the optimal solution faster than traditional GJO.

To improve GJO using Q-learning, need to frame the optimization problem as a Markov Decision Process. The following procedure is followed to connect the Q-learning mechanism with the GJO algorithm.

- The state can be defined as the current position of the search agents in the solution space.
- The actions can be defined as the possible moves a search agent can make in the solution space. For instance, moving towards the Male Jackal, Female Jackal, or a random direction. The reward can be defined based on the improvement in the objective function. If the new position of the search agent results in a better solution, a positive reward is given. Otherwise, a negative reward or zero reward is given.
- Initialize a Q-table with states as rows and actions as columns. Initially, all Q-values can be set to zero.
- Instead of just using the evading energy to decide between exploitation and exploration, use the Q-values to make decisions.
- For each agent, choose an action based on the highest Q-value (with an ε-greedy policy to ensure exploration).
- After taking action, update the Q-values using the Q-learning update rule, as mentioned in Eq. (30).
- After integrating Q-learning, the positions of the search agents will be updated based on the actions chosen from the Q-table rather than just the evading energy.
- Run the algorithm for several episodes to allow the Q-values to converge. An episode here can be defined as a complete run of the maximum iterations.
- After training, the policy (i.e., the best action to take in each state) can be extracted from the Q-table as the action with the highest Q-value for each state.

The Q-values guide the jackal’s movement. The action with the highest Q-value is chosen, directing the jackal towards the Male, Female, or a random direction. The Q-values are updated based on the improvement in the objective function after the move.

The non-linear hunting scheme aims to improve population diversity and prevent premature convergence. The following procedure is used to connect the non-linear hunting scheme with the GJO.

- Use the worst solution to form the differential vector.
- Randomly select jackals to prevent local stagnation.
- Use an exponentially decreasing vector to prevent solutions from sliding towards the geometric centre.

Maintaining diversity in the population is crucial for optimization algorithms. It ensures that the algorithm explores a wide range of solutions and doesn’t converge prematurely to a sub-optimal solution. The non-linear hunting scheme introduces randomness and non-linearity in the update mechanism, ensuring a diverse population. By considering the worst solutions when forming the differential vector, the algorithm pushes the search agents away from poor areas of the solution space. This mechanism ensures that the algorithm does not waste time exploring unpromising regions. The randomized jackal selection strategy prevents a few dominant solutions from overly influencing the entire population. By occasionally considering random jackals for updates, the algorithm ensures that all solutions have a chance to influence the search direction, promoting diversity. The randomness introduced by the non-linear hunting scheme ensures that the algorithm does not quickly converge to a single point in the search space. This is particularly beneficial for complex optimization problems where the solution space has many local optima. The non-linear hunting scheme pushes the search agents towards unexplored regions of the solution space, ensuring a thorough exploration. With an exponentially decreasing probability, apply the non-linear hunting scheme to update the position of the jackal. This ensures that as the iterations progress, the influence of the worst solution and the random jackal decreases, allowing the algorithm to focus more on exploitation rather than exploration.

The integration of Q-learning and the non-linear hunting scheme into GJO provides a multi-faceted enhancement: (i) Q-learning allows the algorithm to adapt based on past experiences, making more informed decisions over time, (ii) The non-linear hunting scheme ensures a diverse population, avoids premature convergence, and makes the algorithm robust against complex optimization landscapes. The combination of Q-learning and the non-linear hunting scheme ensures a balance between exploration and exploitation. The Q-learning component provides a systematic way to make decisions based on past experiences, while the non-linear hunting scheme introduces randomness and diversity, preventing premature convergence to local optima. Together, these strategies equip the GJO algorithm with a more dynamic and adaptive search mechanism, potentially leading to better performance across a wide range of optimization problems. The pseudocode of the proposed RL-GJO algorithm is presented in Algorithm 2.

**Algorithm 2:**
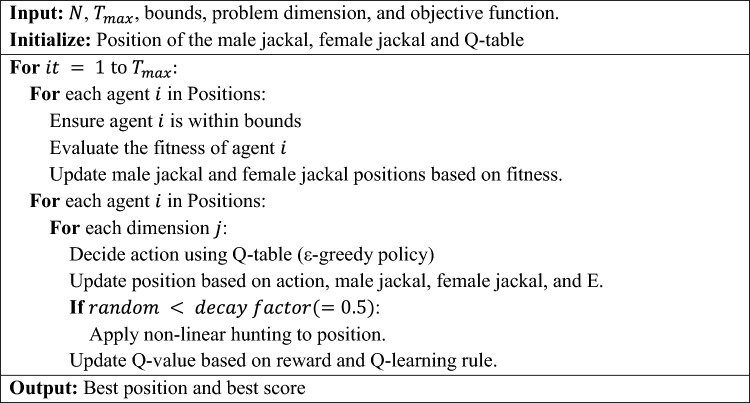
RL-GJO with Q-learning and Non-linear Hunting Scheme.

### Time and space complexity of the RL-GJO

To analyze the time and space complexity of the RL-GJO with Q-learning and the non-linear hunting scheme, let us break down the main components of the algorithm:

*Time Complexity* (i) Initializing the male and female jackals, the positions of the search agents, the Q-table, and the convergence curve takes constant time for each agent and dimension, and the complexity is $O\left({\text{N}}\times {\text{dim}}\right)$, (ii) The main loop runs for the maximum iterations. The complexity of evaluating the fitness of each agent is $O\left({\text{N}}\right)$ , the complexity of the position update based on Q-learning and the non-linear hunting scheme is $O\left({\text{N}}\times {\text{dim}}\right)$, and therefore, the complexity for the main loop is $O\left({T}_{max}\times {\text{N}}\times {\text{dim}}\right)$. Combining the above, the overall time complexity of the RL-GJO algorithm is $O\left({T}_{max}\times {\text{N}}\times {\text{dim}}\right)$. *Space Complexity* (i) The complexity of the positions storage of all search agents $O\left({\text{N}}\times {\text{dim}}\right)$, (ii) The Q-table has an entry for each agent and each possible action (3 actions in our case: move towards the male, move towards the female, or random move), and the complexity is $O\left({\text{N}}\times 3\right)$, (iii) To store the position and score of the male and female jackals, the complexity is $O\left({\text{dim}}\right)$. Combining the above, the overall space complexity of the algorithm is $O\left({\text{N}}\times {\text{dim}}+{\text{N}}+{\text{dim}}+{T}_{max}\right)$, which simplifies to $O\left({\text{N}}\times {\text{dim}}+{T}_{max}\right)]$. In conclusion, the time complexity is primarily influenced by the number of iterations, the number of search agents, and the dimensionality of the problem. The space complexity is mainly determined by the number of search agents and the problem’s dimensionality.

## Results and discussions

This section of the paper showcases the experimental findings that serve to demonstrate the effectiveness of the enhanced algorithm introduced in this research. Firstly, the proposed RL-GJO algorithm is applied to the 29 CEC2017 benchmark numerical problems, and five engineering design problems and then applied to the challenging task of parameter estimation, which is a multimodal problem. Specifically, the proposed is used to solve parameter estimation problems related to SDM, DDM, and TDM across various operating conditions. The objective is to evaluate the reliability of the proposed RL-GJO in handling numerical, real-world, multi-modal problems. To measure the performance of RL-GJO, a comprehensive comparative analysis is carried out, pitting it against seven contemporary state-of-the-art algorithms. These include Opposition-Based GBO (OBGBO)^78^, Reinforced-Learning-based GWO (RLGWO)^79^, GJO, Enhanced Jaya (EJAYA)^80^, Improved Artificial Rabbits Optimization (IARO)^81^, Improved MPA (IMPA)^40^, Adaptive LSHADE (ALSHADE) algorithm^82^, Conscious Neighborhood-based Crow Search Algorithm (CNCSA)^36^, and Quantum-based Avian Navigation Optimizer (QANO)^43^. It is important to note that all these algorithms are meticulously developed and implemented using the MATLAB software tool. The simulations themselves are executed within the MATLAB environment on a laptop system equipped with a 2.5 GHz processor, 16 GB of RAM, and running the Windows 11 operating system. To ensure a fair and consistent evaluation, a common set of parameters is established for all the algorithms involved in the study. These parameters are as follows: a population size of 40, a maximum number of iterations set to 500 for CEC2017 functions and engineering design problems, and 1000 for parameter estimations problems, and the number of independent runs fixed at 30. To determine the relative performance of these algorithms, a statistical test is conducted. Specifically, Friedman’s Ranking Test (FRT) is employed as the assessment metric. This statistical analysis provides a robust and impartial means of ranking the selected algorithms based on their performance. It is crucial to emphasize that the significance and novelty of this study are convincingly substantiated through the utilization of the results obtained from the parameter estimation problems. The experimental outcomes help to establish the unique contributions and advancements made by the proposed RL-GJO in the context of solving complex, real-world, and multimodal problems.

### Results on CEC2017 benchmark problems

This study discusses beyond conventional benchmarking scenarios to rigorously test a newly developed algorithm, leveraging a set of 29 specialized benchmark functions derived from the CEC2017 special session, which focuses on real-parameter optimization. Comprehensive details about these benchmark functions can be found in the referenced document [125]. These 29 functions are systematically categorized into four distinct groups for analytical purposes: (i) Unimodal Functions (F1–F2): This category includes functions with a single peak or trough. However, one of these functions is excluded from consideration due to inherent instability issues; (ii) Multimodal Functions (F3–F9): These functions possess multiple peaks or valleys, offering a more complex landscape for optimization; (iii) Hybrid Functions (F10–F19): This group represents a blend of different function types, creating a varied optimization challenge; (iv) Composition Functions (F20–F29): These functions are composed of multiple elements, each contributing to the overall complexity of the optimization task. In-depth experiments were carried out using this diverse test suite to thoroughly evaluate the performance of the newly proposed algorithm alongside a selection of other algorithms. The assessment process involved recording and analyzing key statistical measures such as the minimum (Min), maximum (Max), average (Avg.), and standard deviation (STD) values. This systematic analysis was aimed at understanding and contrasting the performance nuances of the proposed algorithm against others in various scenarios.

The benchmark functions in the CEC2017 suite were examined across the problem with 10 dimensions. To ensure a comprehensive evaluation, a predefined maximum number of iterations was established as a termination criterion for the experiments, set at 1000 iterations. Each experiment was conducted 30 times for each function to ensure reliability and robustness in the results. The proposed algorithm, termed RL-GJO, was contrasted against a suite of seven other algorithms, some of which are recognized winners of the CEC competition. These algorithms include the Conscious Neighborhood-based Crow Search Algorithm (CNCSA), OBGBO, RLGWO, QANO, IMPA, ALSHADE, and the original GJO algorithm. A crucial aspect of this comparative analysis was the consistent use of controlled variables for all algorithms, which were sourced from the specific literature where each algorithm was initially described. The study includes Table 1, which concisely summarize the statistical outcomes of these comparisons. These tables provide detailed insights by contrasting the benchmarks in 10 dimensions, showcasing the Min, Max, Avg., and STD values obtained from the 30 runs for 29 benchmark functions. In these tables, the most optimal solutions achieved are prominently highlighted in boldface, drawing attention to the superior performance instances.

**Table 1:** Results obtained F1–F29 CEC2017 benchmark functions.

Functions		RL-GJO	IMPA	CNCSA	ALSHADE	RLGWO	QANO	GJO	OBGBO
F1	Min	**1.005E+02**	3.422E+02	7.195E+04	1.049E+02	8.125E+02	9.242E+02	6.952E+02	7.419E+02
	Max	1.555E+02	5.329E+03	8.312E+08	1.748E+03	2.228E+03	1.274E+04	6.322E+03	2.906E+03
	Avg	1.161E+02	2.376E+03	2.344E+08	5.239E+02	1.454E+03	5.440E+03	2.338E+03	2.119E+03
	STD	2.25E+01	2.01E+03	3.65E+08	6.94E+02	6.64E+02	4.56E+03	2.26E+03	8.95E+02
F2	Min	**3.000E+02**	**3.000E+02**	4.044E+03	**3.000E+02**	**3.000E+02**	**3.000E+02**	3.001E+02	3.006E+02
	Max	3.000E+02	3.000E+02	1.171E+04	3.008E+02	3.000E+02	3.000E+02	3.063E+02	3.807E+02
	Avg	3.000E+02	3.000E+02	7.072E+03	3.003E+02	3.000E+02	3.000E+02	3.036E+02	3.168E+02
	STD	1.79E−05	1.52E−04	3.86E+03	3.57E−01	1.88E−03	1.82E−13	2.62E+00	3.57E+01
F3	Min	**4.000E+02**	**4.000E+02**	4.047E+02	4.046E+02	4.002E+02	4.004E+02	4.007E+02	4.063E+02
	Max	4.032E+02	4.042E+02	4.283E+02	4.679E+02	4.046E+02	4.083E+02	4.842E+02	4.107E+02
	Avg	4.017E+02	4.019E+02	4.135E+02	4.180E+02	4.018E+02	4.041E+02	4.196E+02	4.073E+02
	STD	1.30E+00	2.11E+00	1.00E+01	2.79E+01	1.98E+00	3.85E+00	3.62E+01	1.92E+00
F4	Min	**5.030E+02**	5.129E+02	5.111E+02	5.138E+02	5.179E+02	5.159E+02	5.269E+02	5.099E+02
	Max	5.149E+02	5.219E+02	5.288E+02	5.229E+02	5.348E+02	6.040E+02	5.348E+02	5.179E+02
	Avg	5.092E+02	5.155E+02	5.176E+02	5.172E+02	5.255E+02	5.552E+02	5.314E+02	5.133E+02
	STD	4.24E+00	3.76E+00	7.36E+00	3.60E+00	8.16E+00	3.50E+01	3.19E+00	2.95E+00
F5	Min	**6.000E+02**	**6.000E+02**	6.001E+02	**6.000E+02**	6.088E+02	6.016E+02	6.073E+02	**6.000E+02**
	Max	6.001E+02	6.053E+02	6.050E+02	6.020E+02	6.275E+02	6.178E+02	6.198E+02	6.000E+02
	Avg	6.000E+02	6.016E+02	6.025E+02	6.008E+02	6.164E+02	6.088E+02	6.148E+02	6.000E+02
	STD	2.45E−02	2.12E+00	2.34E+00	1.08E+00	6.97E+00	6.93E+00	4.57E+00	8.13E−04
F6	Min	7.209E+02	7.314E+02	7.259E+02	7.210E+02	7.462E+02	7.412E+02	7.460E+02	**7.164E+02**
	Max	7.280E+02	7.709E+02	7.540E+02	7.477E+02	7.662E+02	7.872E+02	7.744E+02	7.225E+02
	Avg	7.254E+02	7.459E+02	7.387E+02	7.323E+02	7.538E+02	7.560E+02	7.593E+02	7.189E+02
	STD	2.80E+00	1.53E+01	1.13E+01	1.04E+01	8.23E+00	1.86E+01	1.41E+01	2.55E+00
F7	Min	8.080E+02	8.129E+02	8.100E+02	**8.040E+02**	8.159E+02	8.119E+02	8.129E+02	8.090E+02
	Max	8.129E+02	8.378E+02	8.375E+02	8.189E+02	8.259E+02	8.468E+02	8.249E+02	8.189E+02
	Avg	8.105E+02	8.235E+02	8.177E+02	8.113E+02	8.229E+02	8.368E+02	8.183E+02	8.155E+02
	STD	2.40E+00	9.35E+00	1.16E+01	6.16E+00	4.04E+00	1.41E+01	5.65E+00	3.83E+00
F8	Min	**9.000E+02**	**9.000E+02**	9.001E+02	**9.000E+02**	9.576E+02	9.023E+02	9.372E+02	**9.000E+02**
	Max	9.001E+02	9.046E+02	9.306E+02	9.619E+02	1.213E+03	2.035E+03	1.397E+03	9.034E+02
	Avg	9.000E+02	9.014E+02	9.079E+02	9.174E+02	1.021E+03	1.299E+03	1.213E+03	9.008E+02
	STD	4.80E−02	1.92E+00	1.31E+01	2.63E+01	1.09E+02	4.91E+02	1.77E+02	1.45E+00
F9	Min	1.155E+03	1.436E+03	1.144E+03	1.486E+03	**1.137E+03**	1.596E+03	1.453E+03	1.148E+03
	Max	1.720E+03	2.508E+03	1.939E+03	1.842E+03	1.842E+03	2.493E+03	2.416E+03	1.868E+03
	Avg	1.456E+03	1.896E+03	1.480E+03	1.663E+03	1.576E+03	2.019E+03	1.982E+03	1.454E+03
	STD	2.25E+02	4.47E+02	3.03E+02	1.42E+02	2.90E+02	3.55E+02	3.56E+02	2.64E+02
F10	Min	**1.104E+03**	1.112E+03	1.114E+03	1.107E+03	1.121E+03	1.121E+03	1.148E+03	**1.104E+03**
	Max	1.107E+03	1.135E+03	1.132E+03	1.207E+03	1.125E+03	1.197E+03	1.216E+03	1.110E+03
	Avg	1.106E+03	1.119E+03	1.125E+03	1.134E+03	1.123E+03	1.147E+03	1.174E+03	1.106E+03
	STD	1.38E+00	9.73E+00	7.69E+00	4.15E+01	1.69E+00	2.95E+01	2.62E+01	2.17E+00
F11	Min	**1.214E+03**	2.957E+03	1.993E+04	3.462E+03	3.865E+03	1.400E+04	5.421E+03	2.356E+03
	Max	1.255E+03	5.817E+04	1.559E+06	1.040E+04	1.232E+04	3.725E+04	4.408E+04	2.434E+04
	Avg	1.236E+03	1.895E+04	4.405E+05	6.274E+03	8.763E+03	2.532E+04	1.764E+04	1.039E+04
	STD	1.82E+01	2.41E+04	6.39E+05	3.06E+03	3.80E+03	1.05E+04	1.52E+04	8.68E+03
F12	Min	**1.304E+03**	1.459E+03	4.075E+03	1.364E+03	6.860E+03	1.634E+03	2.464E+03	1.627E+03
	Max	1.311E+03	2.309E+03	2.778E+04	2.351E+03	9.190E+03	3.448E+03	2.274E+04	1.461E+04
	Avg	1.307E+03	1.828E+03	1.530E+04	1.901E+03	8.477E+03	2.257E+03	8.693E+03	7.306E+03
	STD	3.12E+00	3.91E+02	8.69E+03	4.20E+02	9.24E+02	7.82E+02	8.05E+03	4.81E+03
F13	Min	**1.402E+03**	1.449E+03	1.478E+03	1.413E+03	1.463E+03	1.496E+03	1.474E+03	1.445E+03
	Max	1.406E+03	1.511E+03	5.946E+03	1.500E+03	1.860E+03	2.250E+03	1.561E+03	1.566E+03
	Avg	1.403E+03	1.481E+03	3.703E+03	1.454E+03	1.611E+03	1.665E+03	1.524E+03	1.501E+03
	STD	1.62E+00	2.41E+01	2.12E+03	3.86E+01	1.62E+02	3.28E+02	4.16E+01	4.55E+01
F14	Min	**1.500E+03**	1.536E+03	1.712E+03	1.505E+03	1.531E+03	1.523E+03	1.582E+03	1.627E+03
	Max	1.503E+03	1.707E+03	2.014E+04	1.717E+03	2.556E+03	1.769E+03	2.418E+03	2.911E+03
	Avg	1.501E+03	1.630E+03	9.668E+03	1.593E+03	1.785E+03	1.636E+03	1.919E+03	1.979E+03
	STD	9.24E−01	7.02E+01	8.23E+03	8.38E+01	4.37E+02	9.65E+01	3.09E+02	5.28E+02
F15	Min	**1.602E+03**	1.639E+03	1.613E+03	**1.602E+03**	1.644E+03	1.819E+03	1.723E+03	1.613E+03
	Max	1.606E+03	1.842E+03	1.879E+03	1.781E+03	1.963E+03	2.210E+03	1.953E+03	1.723E+03
	Avg	1.604E+03	1.728E+03	1.694E+03	1.691E+03	1.810E+03	1.982E+03	1.813E+03	1.684E+03
	STD	1.76E+00	8.96E+01	1.17E+02	7.67E+01	1.26E+02	1.62E+02	1.10E+02	5.35E+01
F16	Min	1.718E+03	1.723E+03	1.725E+03	**1.715E+03**	1.753E+03	1.764E+03	1.732E+03	1.735E+03
	Max	1.738E+03	1.794E+03	1.885E+03	1.767E+03	1.801E+03	1.897E+03	1.859E+03	1.756E+03
	Avg	1.726E+03	1.748E+03	1.805E+03	1.739E+03	1.764E+03	1.815E+03	1.792E+03	1.743E+03
	STD	7.63E+00	2.70E+01	7.12E+01	1.89E+01	2.07E+01	5.99E+01	5.85E+01	8.05E+00
F17	Min	**1.802E+03**	2.453E+03	5.287E+03	1.946E+03	1.137E+04	1.967E+03	8.401E+03	6.402E+03
	Max	1.818E+03	2.948E+04	4.916E+04	1.368E+04	3.639E+04	5.542E+04	2.013E+04	3.764E+04
	Avg	1.810E+03	9.671E+03	2.059E+04	4.371E+03	2.296E+04	3.112E+04	1.513E+04	2.312E+04
	STD	6.58E+00	1.13E+04	1.73E+04	5.20E+03	9.68E+03	2.53E+04	5.37E+03	1.15E+04
F18	Min	**1.901E+03**	1.957E+03	2.108E+03	1.907E+03	1.915E+03	1.926E+03	1.912E+03	1.982E+03
	Max	1.903E+03	2.463E+03	2.129E+04	2.045E+03	1.324E+04	1.994E+03	2.010E+03	6.581E+03
	Avg	1.902E+03	2.117E+03	1.157E+04	1.971E+03	5.831E+03	1.956E+03	1.947E+03	3.010E+03
	STD	6.98E−01	1.98E+02	7.62E+03	5.56E+01	5.10E+03	2.78E+01	3.78E+01	2.00E+03
F19	Min	2.022E+03	2.019E+03	2.024E+03	2.021E+03	2.051E+03	2.043E+03	2.061E+03	**2.006E+03**
	Max	2.034E+03	2.169E+03	2.227E+03	2.164E+03	2.164E+03	2.293E+03	2.215E+03	2.045E+03
	Avg	2.028E+03	2.080E+03	2.128E+03	2.055E+03	2.095E+03	2.155E+03	2.123E+03	2.026E+03
	STD	5.62E+00	7.03E+01	7.72E+01	6.10E+01	4.58E+01	1.05E+02	5.65E+01	1.66E+01
F20	Min	**2.200E+03**	2.202E+03	2.205E+03	**2.200E+03**	**2.200E+03**	2.204E+03	2.205E+03	2.305E+03
	Max	2.200E+03	2.330E+03	2.319E+03	2.334E+03	2.329E+03	2.358E+03	2.350E+03	2.311E+03
	Avg	2.200E+03	2.275E+03	2.291E+03	2.296E+03	2.276E+03	2.322E+03	2.309E+03	2.309E+03
	STD	2.08E−02	6.65E+01	4.79E+01	5.44E+01	6.82E+01	6.62E+01	6.04E+01	2.42E+00
F21	Min	**2.231E+03**	2.301E+03	2.303E+03	2.301E+03	2.303E+03	2.302E+03	2.303E+03	2.301E+03
	Max	2.302E+03	2.303E+03	2.323E+03	2.303E+03	2.308E+03	2.311E+03	2.316E+03	2.301E+03
	Avg	2.287E+03	2.301E+03	2.309E+03	2.302E+03	2.306E+03	2.308E+03	2.310E+03	2.301E+03
	STD	3.15E+01	1.01E+00	8.21E+00	1.14E+00	2.14E+00	3.64E+00	4.60E+00	4.04E−01
F22	Min	**2.607E+03**	2.611E+03	2.614E+03	2.613E+03	2.609E+03	2.630E+03	2.634E+03	2.610E+03
	Max	2.617E+03	2.635E+03	2.636E+03	2.621E+03	2.628E+03	2.665E+03	2.694E+03	2.634E+03
	Avg	2.612E+03	2.623E+03	2.621E+03	2.618E+03	2.620E+03	2.643E+03	2.656E+03	2.620E+03
	STD	4.27E+00	8.84E+00	8.56E+00	3.33E+00	7.15E+00	1.39E+01	2.39E+01	9.10E+00
F23	Min	**2.500E+03**	2.744E+03	2.734E+03	2.737E+03	2.738E+03	2.753E+03	2.742E+03	2.742E+03
	Max	2.500E+03	2.765E+03	2.751E+03	2.757E+03	2.771E+03	2.815E+03	2.807E+03	2.748E+03
	Avg	2.500E+03	2.756E+03	2.743E+03	2.747E+03	2.748E+03	2.780E+03	2.762E+03	2.745E+03
	STD	3.35E−02	9.06E+00	6.89E+00	7.54E+00	1.37E+01	2.25E+01	2.68E+01	2.30E+00
F24	Min	2.898E+03	**2.600E+03**	2.915E+03	2.943E+03	2.900E+03	2.900E+03	2.900E+03	2.898E+03
	Max	2.898E+03	2.945E+03	2.944E+03	2.950E+03	2.949E+03	2.970E+03	2.952E+03	2.947E+03
	Avg	2.898E+03	2.867E+03	2.930E+03	2.947E+03	2.919E+03	2.934E+03	2.930E+03	2.926E+03
	STD	5.61E−03	1.50E+02	1.31E+01	2.61E+00	2.63E+01	3.22E+01	2.80E+01	2.50E+01
F25	Min	**2.600E+03**	2.800E+03	2.900E+03	2.900E+03	**2.600E+03**	2.995E+03	3.074E+03	2.800E+03
	Max	2.900E+03	3.169E+03	3.058E+03	3.961E+03	3.138E+03	3.630E+03	4.369E+03	3.845E+03
	Avg	2.820E+03	2.947E+03	2.981E+03	3.142E+03	3.009E+03	3.173E+03	3.914E+03	3.058E+03
	STD	1.30E+02	1.38E+02	5.68E+01	4.60E+02	2.30E+02	2.68E+02	5.79E+02	4.44E+02
F26	Min	**3.090E+03**	3.092E+03	3.096E+03	3.092E+03	3.093E+03	**3.090E+03**	3.097E+03	3.091E+03
	Max	3.090E+03	3.102E+03	3.098E+03	3.181E+03	3.096E+03	3.109E+03	3.163E+03	3.098E+03
	Avg	3.090E+03	3.097E+03	3.097E+03	3.113E+03	3.095E+03	3.101E+03	3.127E+03	3.097E+03
	STD	8.40E−02	3.97E+00	6.12E−01	3.84E+01	1.53E+00	8.51E+00	3.07E+01	3.30E+00
F27	Min	**3.100E+03**	3.219E+03	3.384E+03	3.169E+03	**3.100E+03**	3.412E+03	3.170E+03	3.160E+03
	Max	3.100E+03	3.412E+03	3.413E+03	3.412E+03	3.412E+03	3.736E+03	3.412E+03	3.412E+03
	Avg	3.100E+03	3.335E+03	3.396E+03	3.266E+03	3.238E+03	3.477E+03	3.321E+03	3.312E+03
	STD	2.53E−04	1.06E+02	1.49E+01	1.21E+02	1.51E+02	1.45E+02	1.25E+02	1.36E+02
F28	Min	**3.148E+03**	3.249E+03	3.172E+03	3.173E+03	3.155E+03	3.266E+03	3.176E+03	3.162E+03
	Max	3.177E+03	3.395E+03	3.293E+03	3.308E+03	3.233E+03	3.422E+03	3.504E+03	3.205E+03
	Avg	3.165E+03	3.341E+03	3.222E+03	3.238E+03	3.190E+03	3.328E+03	3.366E+03	3.184E+03
	STD	1.10E+01	5.88E+01	5.20E+01	6.19E+01	3.15E+01	6.36E+01	1.47E+02	1.59E+01
F29	Min	**3.412E+03**	7.044E+03	3.216E+04	4.033E+03	3.957E+03	7.356E+03	6.475E+03	7.521E+03
	Max	3.460E+03	1.252E+06	1.533E+06	1.197E+04	2.006E+04	1.252E+06	1.257E+06	8.207E+05
	Avg	3.440E+03	5.818E+05	7.287E+05	7.572E+03	9.082E+03	4.296E+05	4.326E+05	1.785E+05
	STD	1.92E+01	5.53E+05	7.42E+05	2.88E+03	6.54E+03	5.75E+05	5.75E+05	3.59E+05

Additionally, Table 2 offer a summarized view of the algorithms’ rankings based on their average FRT values. This ranking provides a clear and concise overview of each algorithm’s performance, facilitating an understanding of their relative efficiencies and strengths in tackling the benchmark functions from the CEC2017 suite.

**Table 2:** FRT values obtained by all algorithms for F1–F29 CEC2017 test functions.

Functions	RL-GJO	IMPA	CNCSA	ALSHADE	RLGWO	QANO	GJO	OBGBO
F1	1.400	4.400	8.000	2.200	4.200	6.200	4.400	5.200
F2	2.600	2.400	8.000	5.000	4.000	1.000	6.600	6.400
F3	2.400	2.400	7.000	6.000	2.600	4.600	4.800	6.200
F4	1.400	3.600	3.600	4.600	6.200	7.200	6.800	2.600
F5	2.200	4.400	4.400	3.200	7.400	6.000	7.400	1.000
F6	2.000	5.200	4.200	3.400	7.000	6.200	6.800	1.200
F7	2.000	5.800	3.600	3.000	6.200	7.200	4.600	3.600
F8	2.000	3.400	3.800	4.000	6.600	6.600	7.400	2.200
F9	3.000	5.400	3.000	5.000	3.600	6.600	6.600	2.800
F10	1.400	4.000	5.600	4.800	4.800	6.200	7.600	1.600
F11	1.000	4.200	8.000	3.200	4.200	6.200	5.200	4.000
F12	1.000	3.000	7.000	3.000	6.600	3.400	6.200	5.800
F13	1.000	4.200	6.600	2.800	5.600	6.000	4.800	5.000
F14	1.000	4.000	7.800	3.400	3.800	4.200	5.600	6.200
F15	1.200	4.600	3.800	3.800	6.000	7.200	6.000	3.400
F16	1.800	4.000	6.200	2.600	5.400	6.800	5.800	3.400
F17	1.000	4.000	5.200	2.400	5.800	6.200	5.200	6.200
F18	1.000	5.200	7.400	3.600	5.800	3.800	2.800	6.400
F19	2.800	4.600	5.600	3.200	5.600	6.800	5.800	1.600
F20	1.000	4.600	4.400	5.000	4.600	7.000	5.600	3.800
F21	2.200	2.200	6.400	3.600	6.000	6.600	6.800	2.200
F22	1.600	5.000	4.200	3.400	3.600	6.800	7.600	3.800
F23	1.000	5.800	3.400	5.000	4.000	7.600	5.800	3.400
F24	1.200	4.400	4.200	6.000	4.200	6.400	5.600	4.000
F25	2.400	3.200	4.400	3.800	5.200	6.200	7.600	3.200
F26	1.000	4.800	4.600	4.600	3.400	4.800	7.400	5.400
F27	1.000	4.800	6.000	3.400	3.800	6.800	5.400	4.800
F28	1.400	7.000	4.200	4.200	3.000	6.800	6.200	3.200
F29	1.000	6.000	7.000	3.200	2.400	5.600	5.400	5.400
Average FRT	**1.586**	4.366	5.434	3.841	4.883	5.966	5.993	3.931

The detailed analysis of the algorithm performance, as outlined in the research, focuses heavily on the FRT values recorded in Table 2. For problems with a dimensionality of 10, RL-GJO emerges as the top performer with an FRT value of 1.586, followed by the ALSHADE algorithm with a 3.841 FRT value. Other notable algorithms in the ranking include OBGBO, IMPA, RLGWO, CNCSA, QANO and GJO. The RL-GJO algorithm demonstrates exceptional capabilities in handling a wide range of unconstrained numerical benchmark problems across low, medium, and high dimensions. This is evidenced by the comprehensive comparisons, results, and discussions presented. The algorithm’s performance is notably robust and efficient, successfully attempting various function types, such as unimodal, multimodal, non-separable, separable, composition, and hybrid. The algorithm’s flexibility is highlighted by its consistent performance regardless of the nature of the challenges encountered, including the presence of multiplicative noise in fitness evaluations and rotation transformations.

**Figure 4:**
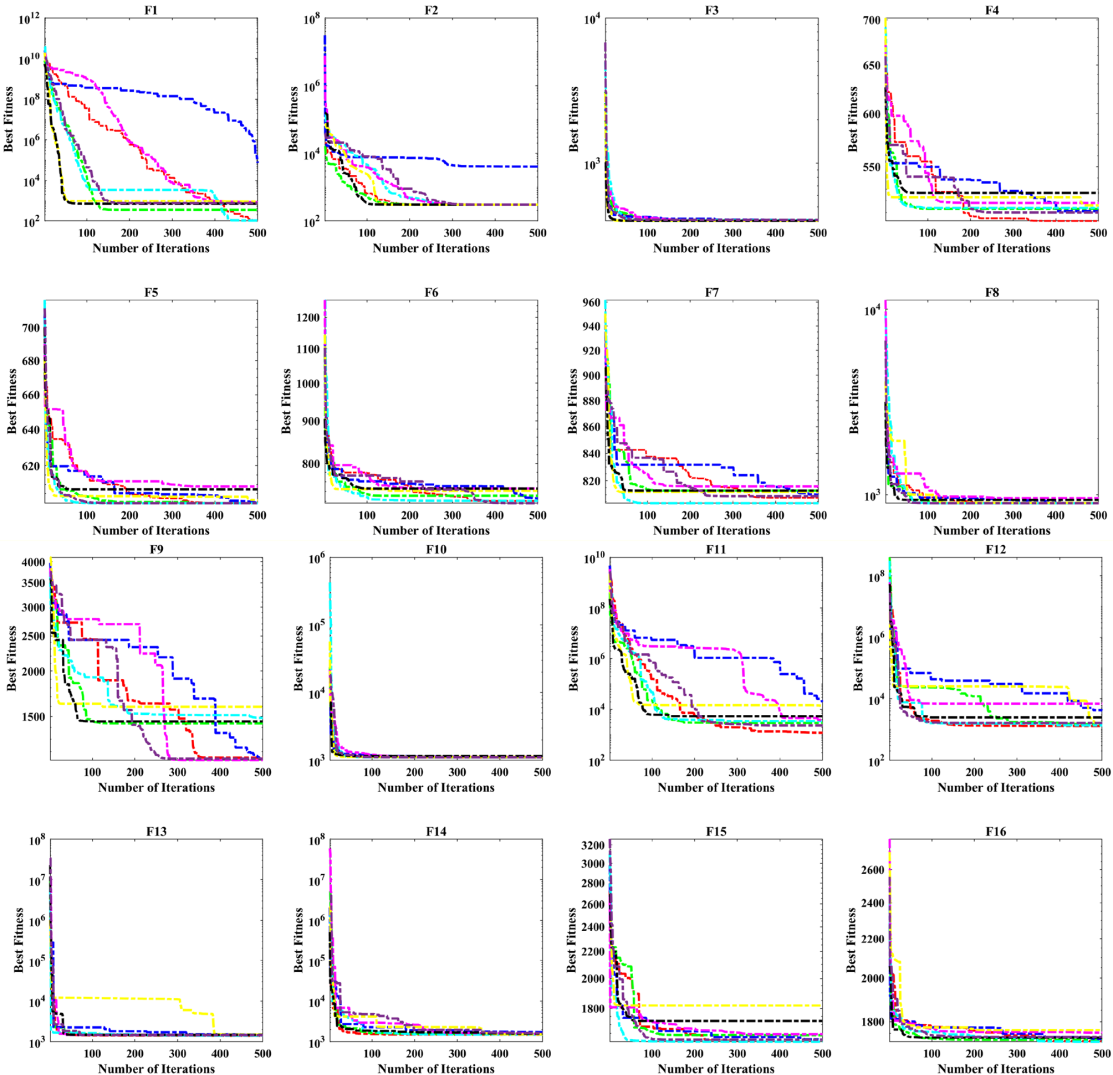

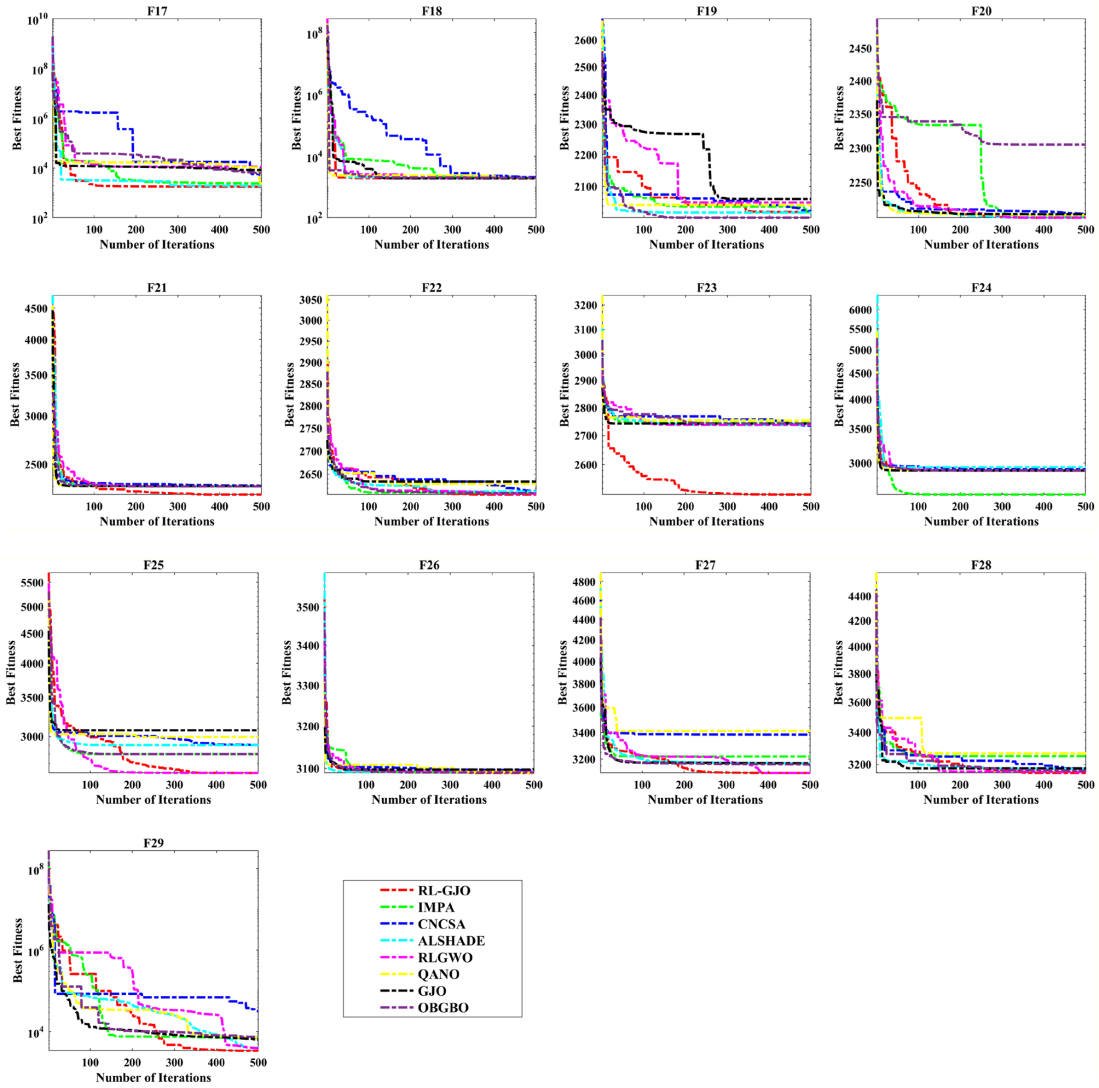
Convergence curves of functions F1-F29 obtained by all algorithms.

A key strength of RL-GJO lies in its ability to effectively balance exploration and exploitation, even in complex optimization landscapes. This balance is attributed to the inclusion of the reinforced learning mechanism within its structure, which significantly enhances the algorithm’s search process. The RL-GJO’s performance is on par with other competitive algorithms, such as ALSHADE, OBGBO, IMPA, RLGWO, CNCSA, QANO, and GJO. To further illustrate the convergence characteristics of RL-GJO and its counterparts, Fig. 4 showcases the optimal objective function values of the median run for various benchmarks in 10 dimensions. Figure 4 reveals that RL-GJO exhibits rapid convergence in the early stages of optimization across a diverse array of function types and complexities. Notably, the algorithm maintains its convergence momentum, with significant enhancements observed in the middle and later phases of the optimization process. The convergence trajectories indicate that RL-GJO can efficiently resolve most problems within a limited number of iterations. The algorithm’s scalability and its ability to maintain a consistent balance between exploitation and exploration up to the maximum iteration limit are key factors in its effectiveness. This efficiency in solving unconstrained problems with fewer iterations is particularly vital for addressing real-world design challenges. Furthermore, as depicted in Fig. 4, RL-GJO shows superior convergence speeds compared to other algorithms, underlining its effectiveness in a wide range of optimization tasks.

### Engineering design optimization problems

In this particular sub-section of the study, the effectiveness of the newly developed RL-GJO algorithm is rigorously evaluated through its application to five distinct constrained engineering design challenges. These challenges include the design of a welded beam, the construction of a pressure vessel, the tension/compression spring problem, the development of a three-bar truss problem, and the design of a tubular column. The selection of these engineering problems is deliberate due to their highly constrained nature, which serves as a robust test of the RL-GJO’s capability in managing constrained optimization scenarios. For a comprehensive assessment, each of the chosen algorithms, including RL-GJO, was put to the test across 30 separate runs. In each of these runs, a consistent population size of 40 was maintained, and the algorithms were allowed to iterate up to a maximum of 500 times. This uniformity in the testing conditions ensures a fair and comparable evaluation of all algorithms involved. To effectively navigate the constraints, a static penalty constraint handling mechanism is adopted. This approach is crucial for dealing with the constraints imposed by the engineering designs, ensuring that the solutions generated remain feasible and practical within the given constraints. The primary goal set for all the selected problems is the minimization of their respective objective functions. This common objective across all problems underscores the optimization focus of the study, where the aim is to find the most efficient, cost-effective, and feasible solutions within the set constraints. This uniform objective allows for a consistent and clear evaluation of the RL-GJO algorithm’s performance in constrained optimization, highlighting its ability to generate optimal solutions in diverse and challenging engineering contexts.

#### Welded beam design problem

**Figure 5:**
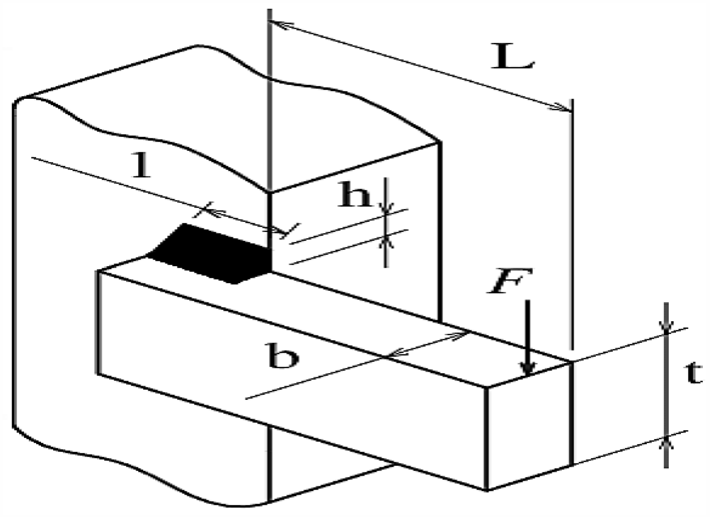
Welded beam design problem.

The primary goal in the welded beam design problem is to achieve the lowest possible cost while adhering to various constraints. This problem involves four key design variables, represented as $x=\left[{x}_{1},{x}_{2},{x}_{3},{x}_{4}\right]$, which correspond to $\left[h,l,t,b\right]$. Here, ${\prime}{l}{\prime}$ represents the length of the beam, ${\prime}{b}{\prime}$ specifies the thickness of the bar, ${\prime}{t}{\prime}$ is the thickness of the weld, and ${\prime}{h}{\prime}$ denotes the height of the beam. The problem is further defined by a set of five equality constraints that must be satisfied. These constraints include the beam bending stress $(\uptheta )$, the shear stress $\left(\uptau \right)$, the buckling load of the bar $\left({P}_{c}\right)$, the deflection at the end of the beam $\left(\updelta \right)$, and various side constraints that must be taken into account. Regarding the design variables, each has been assigned specific upper and lower bounds to ensure feasible solutions. These bounds are defined as follows: the range for ${x}_{1}$ (height) is between 0.1 and 2, for ${x}_{2}$ (length) it’s between 0.1 and 10, for ${x}_{3}$ (weld thickness) the range is also between 0.1 and 10, and for ${x}_{4}$ (bar thickness), the bounds are set between 0.1 and 2. Additional parameters specific to the design problem have also been stipulated. These include a maximum stress $\left({\upsigma }_{max}\right)$ of 30,000 psi, maximum shear stress $\left({\uptau }_{max}\right)$ of 13,600 psi, a modulus of rigidity ($G$) of $12\times {10}^{6}$ psi, an elasticity modulus ($E$) of $30\times {10}^{6}$ psi, a maximum deflection $\left({\updelta }_{max}\right)$ of 0.25 inches, a length ($L$) of 14 inches, and a load ($P$) of 6000 pounds. An illustration of the welded beam design is provided in Fig. 5. Accompanying this design, the specific fitness function and the constraints applicable to the welded beam design problem are outlined, providing a comprehensive framework for the optimization task. This detailed structure of the problem enables a thorough evaluation of the design’s feasibility and cost-effectiveness under the specified conditions and constraints.

32 $$f_{1} \left( x \right) = 1.10471x_{1}^{2} x_{2} + 0.04811x_{3} x_{4} \left( {14 + x_{2} } \right)$$

Subjected to:

33 $$\left. \begin{gathered} g_{1} \left( {\vec{x}} \right) = \tau \left( {\vec{x}} \right) - \tau_{\max } \le 0 \hfill \\ g_{2} \left( {\vec{x}} \right) = \sigma \left( {\vec{x}} \right) - \sigma_{\max } \le 0 \hfill \\ g_{3} \left( {\vec{x}} \right) = \delta \left( {\vec{x}} \right) - \delta_{\max } \le 0 \hfill \\ g_{4} \left( {\vec{x}} \right) = x_{1} - x_{4} \le 0 \hfill \\ g_{5} \left( {\vec{x}} \right) = P - P_{c} \left( {\vec{x}} \right) \le 0 \hfill \\ g_{6} \left( {\vec{x}} \right) = 0.125 - x_{1} \le 0 \hfill \\ g_{7} \left( {\vec{x}} \right) = 1.10471x_{1}^{2} + 0.04811x_{3} x_{4} \left( {14.0 + x_{2} } \right) - 5.0 \le 0 \hfill \\ \end{gathered} \right\}$$

The comparative performance of the RL-GJO algorithm against other notable algorithms like OBGBO, IMPA, RLGWO, QANO, CNCSA, ALSHADE, and GJO is systematically tabulated in Table 3. This table reveals that RL-GJO has achieved superior performance, yielding the lowest cost in comparison to its counterparts. Additionally, Table 3 provides a detailed statistical breakdown, including the Min, Mean (Avg.), STD, and RT for each algorithm. Based on this comprehensive data, it becomes evident that the RL-GJO algorithm is a more effective and reliable choice for optimizing the welded beam design problem. Visual representations in the form of convergence curves and boxplot analyses further enrich the performance evaluation.

**Table 3:** Results obtained for the welded beam problem.

Algorithm	$h$	$l$	$t$	$b$	Min	Avg	STD	RT	FRT
**RL-GJO**	0.18415	3.69397	9.04412	0.20576	**1. 6952**	**1.6966**	**0.000392**	0.159	**1.6**
CNCSA	0.20571	3.25922	9.03710	0.20574	1.6962	1.7382	0.016913	7.219	5.4
QANO	0.21318	3.41794	8.96561	0.21287	1.7801	1.8196	0.029624	0.139	7
IMPA	0.20189	3.32062	9.06531	0.20560	1.7026	1.7124	0.00953	0.250	4
RLGWO	1.12637	2.00000	2.00000	2.00000	1.09E+14	1.09E+14	238,735.8	0.244	8
OBGBO	0.20574	3.25302	9.03662	0.20573	1.7285	1.7347	0.05397	0.466	3
GJO	0.20742	3.23814	8.99110	0.20827	1.7069	1.7229	0.017246	**1.134**	4.4
ALSHADE	0.20539	3.26299	9.03675	0.20590	1.6974	1.6992	0.001715	1.469	2.6

Figure 10a illustrates the convergence patterns of all the algorithms, offering insights into their optimization progress over time. Figure 11a, on the other hand, presents a boxplot analysis, which provides a visual summary of the distribution and variability of the results obtained by each algorithm. These figures complement the numerical data in Table 3, offering a more holistic view of the algorithms’ performances. In addition to these graphical representations, the FRT values generated by all the algorithms are also presented, offering another dimension for performance comparison. The comprehensive data, both in tabular and graphical forms, underscores the efficacy of the RL-GJO algorithm. This robust evaluation demonstrates that RL-GJO stands out as the most effective algorithm for addressing the challenges of the welded beam design optimization problem.

#### Pressure vessel design problem

**Figure 6:**
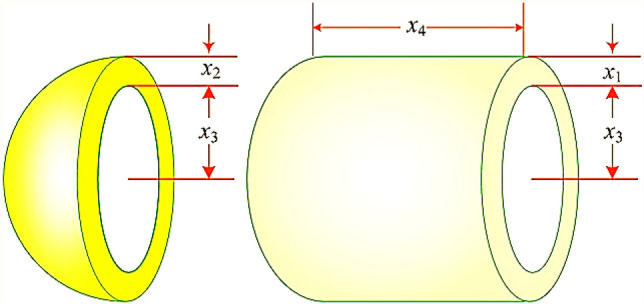
Pressure vessel design problem.

Figure 6 presents a detailed schematic illustration of the pressure vessel design optimization problem. This particular design of the pressure vessel includes capped ends and hemispherical heads. The main goal of this optimization problem is to minimize the costs associated with the vessel’s construction. The optimization process involves considering four primary control variables, denoted as $x=\left[{x}_{1},{x}_{2},{x}_{3},{x}_{4}\right]=\left[{T}_{s},{T}_{h},R,L\right]$. In this notation, ${T}_{s}$ represents the thickness of the shell, ${T}_{h}$ stands for the thickness of the head, $R$ is the inner radius of the vessel, and $L$ signifies the length of the cylindrical section of the vessel. Furthermore, the problem is constrained by four equality conditions, which are comprehensively detailed in Eq. (35). These constraints are crucial for ensuring that the design remains practical and viable while optimizing for cost. The range of values that these variables can take is also specified. The thicknesses of the shell and the head are constrained to values between 0 and 99, while the inner radius and the length of the cylindrical section are limited to a range of 10 to 200. The primary objective function, which is to minimize the construction cost of the pressure vessel, is formulated in Eq. (34). This equation is critical as it quantitatively defines the goal of the optimization problem, guiding the design process towards the most cost-effective solution while adhering to the specified constraints and variable bounds.

34 $$f_{2} \left( x \right) = 0.6224x_{1} x_{3} x_{4} + 1.7781x_{2} x_{3}^{2} + 3.1661x_{1}^{2} x_{4} + 19.84x_{1}^{2} x_{3}$$

Subjected to:

35 $$\left. {\begin{array}{*{20}c} {g_{1} \left( x \right) = - x_{1} + 0.0193x} \\ {g_{2} \left( x \right) = - x_{2} + 0.00954x_{3} \le 0} \\ {g_{3} \left( x \right) = - \pi x_{3}^{2} x_{4} - \left( {4/3} \right)\pi x_{3}^{3} + 1,296,000 \le 0} \\ {g_{4} \left( x \right) = x_{4} - 240 \le 0} \\ \end{array} } \right\}$$

The comparative performance of the RL-GJO algorithm against other notable algorithms like OBGBO, IMPA, RLGWO, QANO, CNCSA, ALSHADE, and GJO is systematically tabulated in Table 4. This table reveals that RL-GJO has achieved superior performance, yielding the lowest cost in comparison to its counterparts. Additionally, Table 4 provides a detailed statistical breakdown, including the Min, Avg., STD, and RT for each algorithm. Based on this comprehensive data, it becomes evident that the RL-GJO algorithm is a more effective and reliable choice for optimizing the pressure vessel design problem. Visual representations in the form of convergence curves and boxplot analyses further enrich the performance evaluation. Figure 10b illustrates the convergence patterns of all the algorithms, offering insights into their optimization progress over time. Figure 11b, on the other hand, presents a boxplot analysis, which provides a visual summary of the distribution and variability of the results obtained by each algorithm. These figures complement the numerical data in Table 4, offering a more holistic view of the algorithms’ performances. In addition to these graphical representations, the FRT values generated by all the algorithms are also presented, offering another dimension for performance comparison. The comprehensive data, both in tabular and graphical forms, underscores the efficacy of the RL-GJO algorithm. This robust evaluation demonstrates that RL-GJO stands out as the most effective algorithm for addressing the challenges of the pressure vessel design optimization problem.

**Table 4:** Results obtained for the pressure vessel design problem.

Algorithm	${T}_{s}$	${T}_{h}$	$R$	$L$	Min	Avg	STD	RT	FRT
**RL-GJO**	1.0953	6.86E−08	65.2263	10.0000	**2302.6073**	**2302.8113**	**2.137E−01**	0.066	**2.2**
CNCSA	1.0926	0.0000	65.2258	10.0000	2302.6784	2304.9729	5.219E+00	4.247	**2.2**
QANO	1.0561	0.0000	65.5193	10.0682	2353.4406	5317.5201	1.657E+03	0.059	7
IMPA	0.7510	0.0000	51.5408	86.6073	3411.9556	3589.6224	9.939E+01	0.097	5.8
RLGWO	1.0141	0.0000	64.4010	23.5235	2870.4430	8013.0051	6.084E+03	0.188	6.6
OBGBO	1.0936	0.0000	65.2252	10.0000	2302.5464	4554.2972	2.056E+03	0.131	4.4
GJO	1.0921	0.0000	65.2266	10.0000	2302.7046	2304.6704	2.232E+00	**0.056**	3.6
ALSHADE	1.0912	0.0000	65.2290	10.0018	2303.0586	3053.6712	1.678E+03	1.016	4.2

#### Tension/compression spring design problem

**Figure 7:**
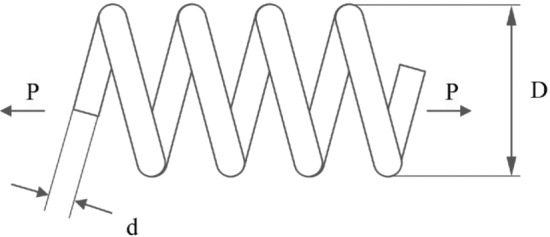
Tension/compression spring design problem.

The study also examines the classic mechanical engineering challenge of designing a tension/compression spring, with the primary goal being to minimize the weight of the spring. This design problem is visually represented in Fig. 7. The optimization process for the spring design involves three key control variables, which are expressed as $x=\left[{x}_{1},{x}_{2},{x}_{3}\right]=\left[d,D,N\right]$. In this context, ‘$d$’ refers to the wire diameter, ‘$D$’ represents the mean coil diameter, and ‘$N$’ stands for the number of active coils in the spring. Additionally, the problem is defined by four equality constraints that are crucial for ensuring a viable and functional design. These constraints are detailed in Eq. (37), serving as essential parameters that must be met in the design process. The range within which these variables can operate is also specified. The wire diameter is limited to values between 0.05 and 2, the mean coil diameter has a permissible range from 0.25 to 1.3, and the number of active coils can vary from 2 to 15. These bounds are set to ensure that the spring design remains practical and feasible within the realms of mechanical engineering principles. The primary objective function for this design problem, aimed at reducing the weight of the tension/compression spring, is formulated in Eq. (36). This objective function is critical as it quantitatively guides the optimization process, focusing on achieving a lightweight yet functional spring design while adhering to the specified constraints and variable limits.

36 $${f}_{3}\left(\overrightarrow{x}\right)=\left({x}_{3}+2\right){x}_{2}{x}_{1}^{2}$$

Subjected to:

37 $$\left.\begin{array}{c}{g}_{1}\left(\overrightarrow{x}\right)=1-\frac{{x}_{2}^{3}{x}_{3}}{71785{x}_{1}^{4}}\le 0\\ {g}_{2}\left(\overrightarrow{x}\right)=\frac{4{x}_{2}^{2}-{x}_{1}{x}_{2}}{12566\left({x}_{2}{x}_{1}^{3}-{x}_{1}^{4}\right)}+\frac{1}{5108{x}_{1}^{2}}-1\le 0\\ {g}_{3}\left(\overrightarrow{x}\right)=1-\frac{140.45{x}_{1}}{{x}_{2}^{2}{x}_{3}}\le 0\\ {g}_{4}\left(\overrightarrow{x}\right)=\frac{{x}_{1}+{x}_{2}}{1.5}-1\le 0\end{array}\right\}$$

Table 5 in the study presents a comparative analysis of the results achieved by the RL-GJO algorithm against other notable algorithms, including OBGBO, IMPA, RLGWO, QANO, CNCSA, ALSHADE, and GJO, particularly in the context of the tension/compression spring design optimization problem. This table highlights that RL-GJO has excelled above all other algorithms in terms of performance, achieving the lowest weight for the spring design. The table not only showcases the superiority of RL-GJO in terms of optimal weight but also provides a detailed statistical analysis. It includes data points such as the Min, Avg., STD, and RT for each algorithm. This comprehensive statistical information solidifies the conclusion that the RL-GJO algorithm is a highly effective and reliable tool for optimizing the design of tension/compression springs.

**Table 5:** Results obtained for the tension/compression spring design problem.

Algorithm	$d$	$D$	$P$	Min	Avg	STD	RT	FRT
**RL-GJO**	0.13914	1.30000	11.89149	**3.6619**	**3.6619**	**3.889E−06**	0.084	**2**
CNCSA	0.13915	1.30000	11.89396	**3.6619**	**3.6619**	3.802E−05	7.650	2.4
QANO	0.13892	1.30000	11.78184	3.6682	3.6847	3.229E−02	0.089	7
IMPA	0.13917	1.30000	11.89925	**3.6619**	3.6620	1.663E−04	0.159	4.2
RLGWO	0.10417	1.55717	1.98983	25.5095	27.0522	1.638E+00	0.096	8
OBGBO	0.13915	1.30000	11.89243	**3.6619**	3.6775	3.489E−02	0.234	2.6
GJO	0.13902	1.30000	11.87082	3.6624	3.6626	1.912E−04	**0.081**	5.8
ALSHADE	0.13916	1.30000	11.89336	**3.6619**	**3.6619**	3.026E−05	1.141	4

To further elaborate on the comparative performance, the study includes Fig. 10c, which displays the convergence curves for all the algorithms. These curves offer insights into how each algorithm progresses towards finding the optimal solution over time. Additionally, Fig. 11c provides a boxplot analysis of all the selected algorithms. This type of analysis offers a visual representation of the distribution and range of the results obtained by each algorithm, further demonstrating the effectiveness of RLGJO in this context. Moreover, the study also presents the FRT values derived by each algorithm, adding another layer of comparative data. These values help in understanding the efficiency of each algorithm in terms of computation time. Overall, the RL-GJO emerges as the most effective solution in addressing the challenges of the tension/compression spring design problem, as evidenced by its top performance in Table 5 and the graphical analyses in Figs. 10c and 11c.

#### Three-bar truss design problem

**Figure 8:**
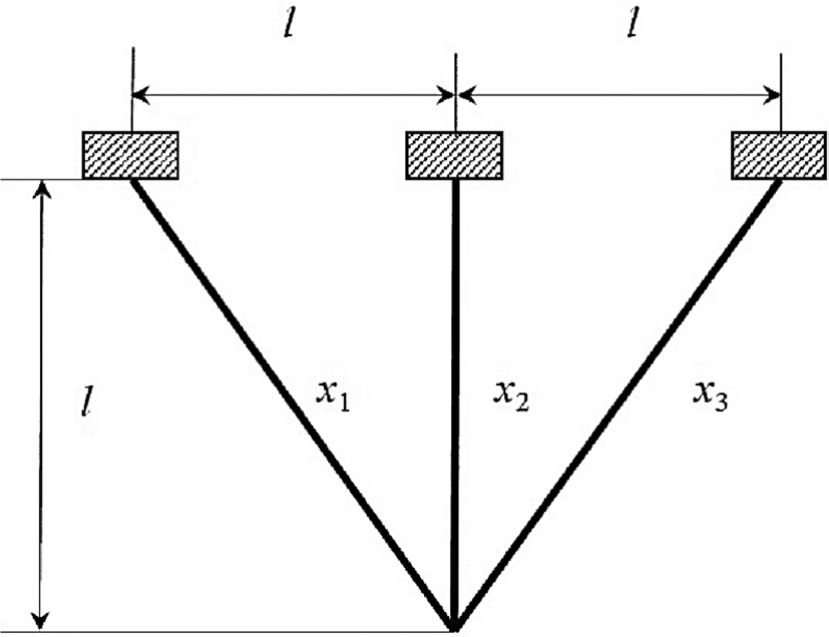
Three-bar truss design problem.

The aim of the three-bar truss design problem is focused on minimizing the overall weight of the truss structure. This problem is defined by three key equality constraints, which address critical aspects of the truss’s functionality: the stress experienced by each bar, the potential for buckling, and the degree of deflection. To navigate these constraints, the problem is formulated with three control variables, represented as $x=\left[{x}_{1},{x}_{2},{x}_{3}={x}_{1}\right]=\left[{A}_{1},{A}_{2}\right]$. These variables, ${A}_{1}$ and ${A}_{2}$, essentially denote the cross-sectional areas of the bars in the truss. The range for each of these control variables is set between 0 and 1, ensuring that the solutions remain within practical and feasible limits. The problem also incorporates certain fixed parameters that are critical to the design. These include the length of the bars ($l$), set at 100 cm, the applied load ($P$), which is $2kN/c{m}^{2}$, and the allowable stress $\left(\upsigma \right)$, also at $2kN/c{m}^{2}$. These parameters play a crucial role in defining the physical and operational context of the truss design. The primary objective of this optimization problem, which is to minimize the weight of the truss, is detailed in Eq. (38). This equation provides a quantitative goal for the optimization process. Additionally, the specific equality constraints that the design must adhere to are enumerated in Eq. (39). These constraints are fundamental in ensuring that the truss design is not only lightweight but also structurally sound and capable of withstanding the specified loads and stresses. A visual representation of the three-bar truss structure is provided in Fig. 8. This illustration gives a clearer understanding of the truss’s configuration, aiding in the comprehension of how the control variables and constraints come together in the practical context of the design problem.

38 $${f}_{4}\left(\overrightarrow{x}\right)=\left(2\sqrt{2}{x}_{1}+{x}_{2}\right)*l$$

Subject to:

39 $$\left.\begin{array}{c}{g}_{1}\left(\overrightarrow{x}\right)= \frac{2\sqrt{2}{x}_{1}+{x}_{2}}{\sqrt{2}{{x}_{1}}^{2}+2{{x}_{1}x}_{2}}, P-\sigma \le 0\\ {g}_{2}\left(\overrightarrow{x}\right)= \frac{{x}_{2}}{\sqrt{2}{{x}_{1}}^{2}+2{{x}_{1}x}_{2}}, P-\sigma \le 0\\ {g}_{3}\left(\overrightarrow{x}\right)= \frac{1}{\sqrt{2}{x}_{2}+{x}_{1}}, P-\sigma \le 0\end{array}\right\}$$

The comparative performance of the RL-GJO algorithm against other notable algorithms like OBGBO, IMPA, RLGWO, QANO, CNCSA, ALSHADE, and GJO is systematically tabulated in Table 6. This table reveals that RL-GJO has achieved superior performance, yielding the lowest cost in comparison to its counterparts. Additionally, Table 6 provides a detailed statistical breakdown, including the Min, Avg., STD, and RT for each algorithm. Based on this comprehensive data, it becomes evident that the RL-GJO algorithm is a more effective and reliable choice for optimizing the three-bar truss design problem. Visual representations in the form of convergence curves and boxplot analyses further enrich the performance evaluation. Figure 10d illustrates the convergence patterns of all the algorithms, offering insights into their optimization progress over time. Figure 11d, on the other hand, presents a boxplot analysis, which provides a visual summary of the distribution and variability of the results obtained by each algorithm. These figures complement the numerical data in Table 6, offering a more holistic view of the algorithms’ performances. In addition to these graphical representations, the FRT values generated by all the algorithms are also presented, offering another dimension for performance comparison. The comprehensive data, both in tabular and graphical forms, underscores the efficacy of the RL-GJO algorithm. This robust evaluation demonstrates that RL-GJO stands out as the most effective algorithm for addressing the challenges of the three-bar truss design optimization problem.

**Table 6:** Results obtained for the three-bar truss design problem.

Algorithm	$x1$	$x2$	Min	Avg	STD	RT	FRT
**RL-GJO**	0.78684	0.28802	**186.3859**	**186.3859**	**4.044E−06**	0.058	**1**
CNCSA	0.78683	0.28804	**186.3859**	186.3860	5.033E−05	6.525	2.8
QANO	0.78681	0.28803	**186.3859**	189.0871	6.031E+00	0.066	7
IMPA	0.78687	0.28797	**186.3859**	**186.3859**	5.096E−06	0.159	2.6
RLGWO	0.78699	0.28739	186.3873	186.4133	4.641E−02	0.059	7.4
OBGBO	0.78685	0.28801	**186.3859**	186.3859	1.421E−04	0.062	4
GJO	0.78747	0.28663	186.3864	186.3882	2.088E−03	**0.041**	6.4
ALSHADE	0.78693	0.28781	**186.3859**	186.3861	1.780E−04	0.978	4.8

#### Tubular column design problem

**Figure 9:**
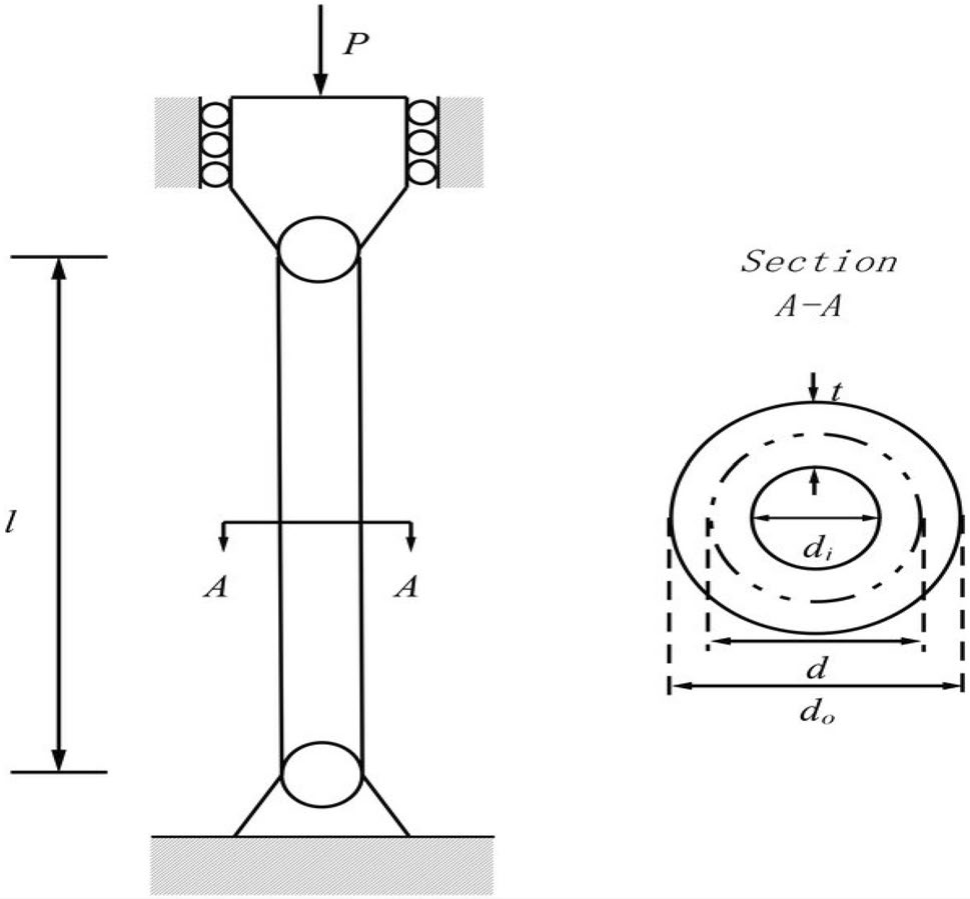
Structure of the tubular column design.

The goal of this problem is to construct a uniform tubular column capable of supporting a compressive load $P$ of 2500 kgf at the lowest possible cost, with hinge joints positioned at both ends of the column. An illustrative depiction of this tubular column design is provided in Fig. 9. The column is fabricated from a material that possesses specific mechanical properties. These include a yield strength $\left({\upsigma }_{y}\right)$ of 500 kgf/cm^3^, an elastic modulus ($E$) of $0.85\times {10}^{6}$ kgf/cm^2^, and a weight density $\left(\uprho \right)$ of 0.0025 kgf/cm^3^. The column has a total length ($L$) of 250 cm. Two critical constraints are applied to the design of this column. The first constraint (${g}_{1}$) ensures that the stress within the column does not exceed the material’s yield strength. The second constraint (${g}_{2}$) relates to the buckling stress, which must not be surpassed to maintain structural integrity. The design parameters include the average diameter of the column, which is restricted to a range between 2 and 14 cm. Furthermore, the market availability of the column restricts the thickness range to between 0.2 cm and 0.8 cm. These limitations are important for ensuring the practicality and feasibility of the column design. The cost of the column is calculated based on a formula: $5W+2d$, where $W$ represents the weight of the column in kilograms of force and d denotes the average diameter in centimetres. This cost function aims to balance the material costs against the structural requirements of the column. The objective function, aimed at optimizing the design for cost while adhering to the constraints, is outlined in Eq. (40). Additionally, six equality constraints that govern the design parameters and structural integrity are detailed in Eq. (40). To achieve this optimization, two control variables are considered, represented as $x=\left[{x}_{1},{x}_{2}\right]=\left[d,t\right]$, where ‘$d$’ stands for the average diameter and ‘$t$’ for the thickness of the tubular column. These control variables are key to navigating the design space within the specified constraints and achieving the desired balance of cost, strength, and structural feasibility.

40 $${f}_{5}\left(\overrightarrow{x}\right)=9.8\times d\times t+2\times d$$

Subject to:

41 $$\left.\begin{array}{c}\begin{array}{c}{g}_{1}\left(\overrightarrow{x}\right)= \frac{P}{\pi dt}\le {\sigma }_{y}\\ {g}_{2}\left(\overrightarrow{x}\right)= \frac{P}{\pi dt}-\frac{{\pi }^{2}E\left({d}^{2}+{t}^{2}\right)}{8{L}^{2}}\le 0\\ {g}_{3}\left(\overrightarrow{x}\right)= -d+2\le 0\end{array}\\ {g}_{4}\left(\overrightarrow{x}\right)= d-14\le 0\\ {g}_{5}\left(\overrightarrow{x}\right)= -t+0.2\le 0\\ {g}_{6}\left(\overrightarrow{x}\right)= t-0.8\le 0\end{array}\right\}$$

**Figure 10:**
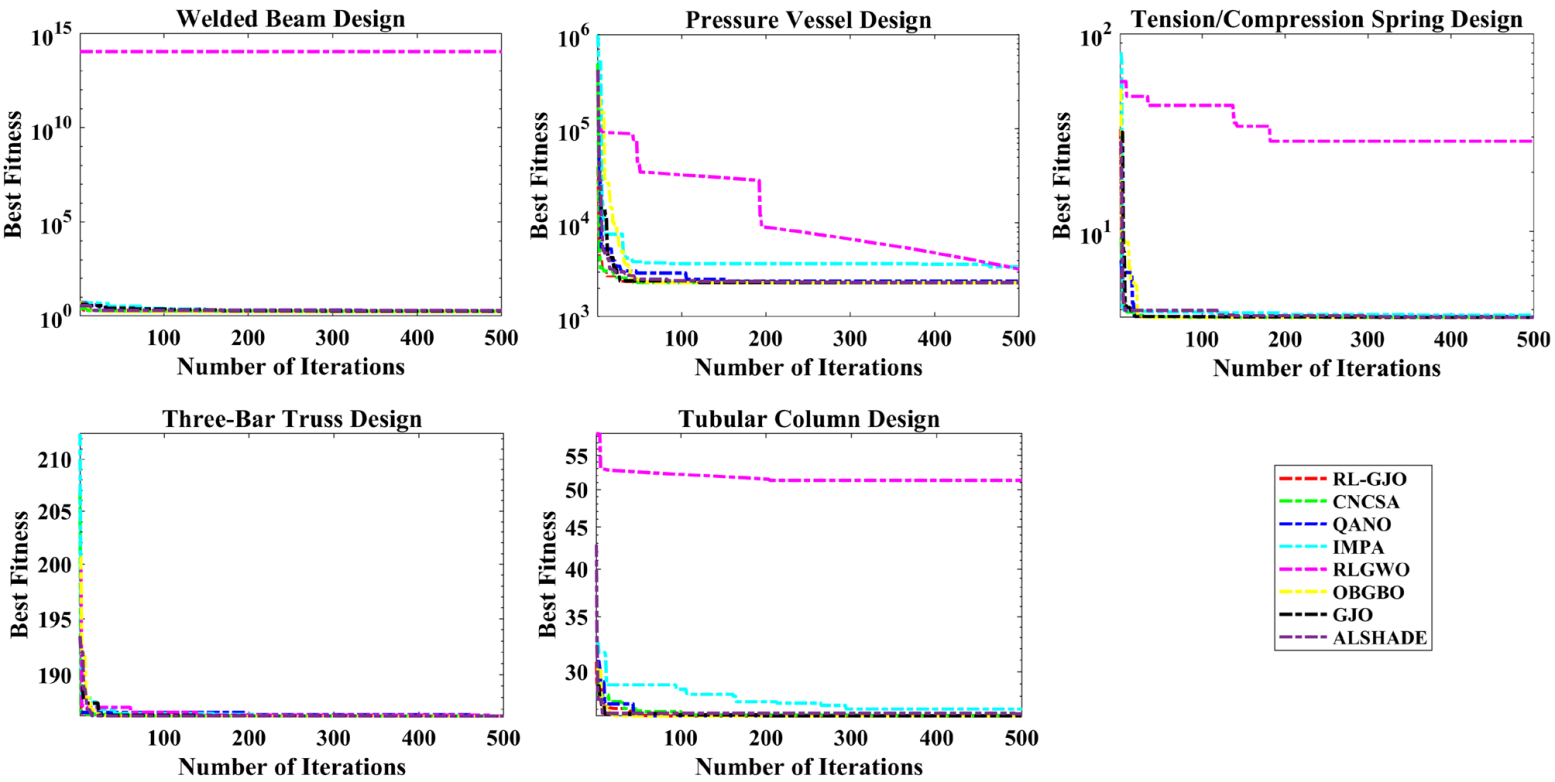
Convergence curves of all algorithms; (**a**) Welded beam design, (**b**) Pressure vessel design, (**c**) Tension/compression spring design, (**d**) Three-bar truss design, (**e**) Tubular column design.

**Figure 11:**
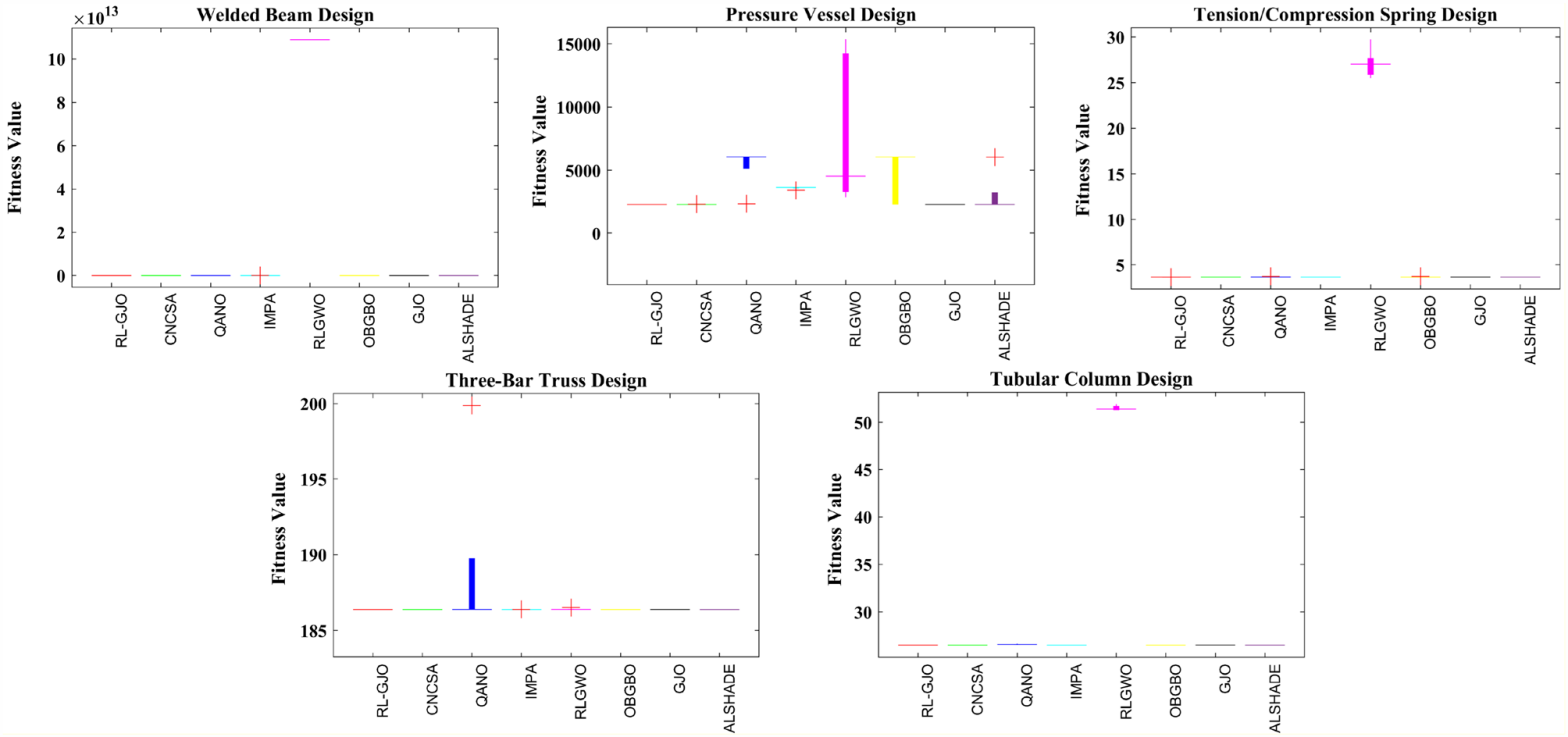
Boxplots of all algorithms; (**a**) Welded beam design, (**b**) Pressure vessel design, (**c**) Tension/compression spring design, (**d**) Three-bar truss design, (**e**) Tubular column design.

The comparative performance of the RL-GJO algorithm against other notable algorithms like OBGBO, IMPA, RLGWO, QANO, CNCSA, ALSHADE, and GJO is systematically tabulated in Table 7. This table reveals that RL-GJO has achieved superior performance, yielding the lowest cost in comparison to its counterparts. Additionally, Table 7 provides a detailed statistical breakdown, including the Min, Avg., STD, and RT for each algorithm. Based on this comprehensive data, it becomes evident that the RL-GJO algorithm is a more effective and reliable choice for optimizing the tubular column design problem. Visual representations in the form of convergence curves and boxplot analyses further enrich the performance evaluation. Figure 10e illustrates the convergence patterns of all the algorithms, offering insights into their optimization progress over time. Figure 11e, on the other hand, presents a boxplot analysis, which provides a visual summary of the distribution and variability of the results obtained by each algorithm. These figures complement the numerical data in Table 7, offering a more holistic view of the algorithms’ performances. In addition to these graphical representations, the FRT values generated by all the algorithms are also presented, offering another dimension for performance comparison. The comprehensive data, both in tabular and graphical forms, underscores the efficacy of the RL-GJO algorithm. This robust evaluation demonstrates that RL-GJO stands out as the most effective algorithm for addressing the challenges of the tubular column design optimization problem.

**Table 7:** Results obtained for the tubular column design problem.

Algorithm	$d$	$t$	Min	Avg	STD	RT	FRT
**RL-GJO**	5.45304	0.29155	**26.4854**	**26.4854**	**4.417E−10**	0.169	**1**
CNCSA	5.45294	0.29153	26.4858	26.4863	8.297E−04	1.894	3
QANO	5.46538	0.29066	26.5188	26.5729	5.303E−02	0.103	7
IMPA	5.45294	0.29152	26.4857	26.4865	8.149E−04	0.066	2.4
RLGWO	2.00000	2.41291	51.2931	51.5150	2.722E−01	0.050	8
OBGBO	5.45273	0.29154	26.4869	26.4874	1.692E−04	0.059	4.2
GJO	5.45644	0.29133	26.4926	26.4962	3.586E−03	**0.047**	5.8
ALSHADE	5.45290	0.29156	26.4864	26.4898	4.242E−03	0.841	4.6

### Photovoltaic model parameter estimation problem

As previously discussed, the RL-GJO is primarily employed in the context of distinct PV models: SDM, DDM, TDM, and the characterization of PV modules, aimed at determining their unknown parameters. To rigorously evaluate its performance, this experimental study draws upon three distinct datasets. The first dataset encompasses 26 pairs of voltage and current measurements taken from an RTC France Si solar cell. These measurements were captured under specific environmental conditions, with an irradiance level of 1000 W/m^2^ and a temperature of 33 °C. The second dataset comprises 44 pairs of the voltage and current data collected from PVM752 GaAs thin-film PV cells. These measurements were acquired under 1000 W/m^2^ irradiation and 25 °C temperature. The third dataset comprises data collected from a Photowatt-PWP201 PV module. Similarly, these measurements were acquired under standardized conditions, featuring an irradiance of 1000 W/m^2^ and a temperature of 45 °C. Consequently, within the scope of this research, both the SDM, DDM, and TDM identification experiments were conducted for the RTC France Si cell, the Photowatt-PWP201 PV module, and the PVM752 GaAs thin-film PV cell. In order to effectively execute these experiments and apply the proposed RL-GJO, it is essential to establish the upper and lower bounds for all the unknown parameters associated with various PV models. These bounds, defining the permissible range of these parameters, are meticulously detailed in Table 8. This information is instrumental in guiding the parameter estimation process and ensuring that the algorithm operates within the accurate boundaries of the problem space.

**Table 8:** Limits of various parameters of PV models.

Parameters	RTC France Si PV Cell		Photowatt-PWM201 PV Module		PVM752 GaAs Thin-Film Cell		SM55 PV Module	
	$ub$	$lb$	$ub$	$lb$	$ub$	$lb$	$ub$	$lb$
${I}_{ph}$(A)	1	0	8	0	0.5	0	2 ${I}_{sc}$	0
${a}_{1}$ and ${a}_{2}$	2	1	50	1	2	1	5	1
${a}_{3}$	5	2	50	1	5	2	5	1
${I}_{sd1}$*,* ${I}_{sd2}$ and ${I}_{sd3}$ (µA)	1	0	50	0	1	0	100	0
*R*_*se*_ (Ω)	0.5	0	0.4	0	0.8	0	2	0
*R*_*sh*_ (Ω)	100	0	1500	0	1000	0	5000	0

The comparative assessment of algorithm performance involves a comprehensive examination of various metrics. These evaluations are based on several key factors, including the RMSE values, performance indicators such as Relative Error (RE), Absolute Error (AE), Mean Bias Error (MBE), and R^2^, statistical metrics encompassing Minimum (Min), Maximum (Max), Mean, Median, Standard Deviation (STD), and Runtime (RT). The expressions to find the RE, AE, MBE, and R^2^ are provided as follows.

42 $$RE=\frac{\left|{I}_{ex}-{I}_{es}\right|}{{I}_{ex}}$$

43 $$AE=\left|{I}_{ex}-{I}_{es}\right|$$

44 $$MBE=\frac{1}{N}\sum_{i=1}^{N}{\left({I}_{ex}-{I}_{es}\right)}^{2}$$

45 $${R}^{2}=1-\frac{\sum_{i=1}^{N}{\left({I}_{ex}-{I}_{es}\right)}^{2}}{\sum_{i=1}^{N}{\left({I}_{ex}-{I}_{ex,av}\right)}^{2}}, {I}_{ex,av}= \frac{1}{N}\sum_{i=1}^{N}{\left({I}_{ex}\right)}^{2}$$

where ${I}_{ex}$ signifies the experimental current, ${I}_{es}$ represents the estimated current, ${I}_{ex,av}$ signifies the average value of the experimental current, and $N$ denotes the number of data samples. Furthermore, a statistical test called FRT is applied. Additionally, the convergence graph and boxplots are utilized to visualize and interpret the performance data. As previously mentioned, each algorithm undergoes 30 runs to provide a robust assessment of its reliability and consistency. In line with this, the RMSE plays a pivotal role in quantifying the dissimilarity between estimated and experimental values. A lower RMSE value indicates a closer alignment between the estimated parameters and the empirical data. This, in turn, signifies the superior effectiveness of the chosen method in characterizing unidentified parameters within the PV cell or PV module. In simpler terms, algorithms that yield lower RMSE values tend to produce models that more accurately represent the true characteristics of the solar cell or module. Consequently, minimizing the error value is of significant importance in this context, as it signifies the algorithm’s ability to deliver results with the highest degree of accuracy possible.

#### Results of SDM/DDM/TDM of RTC France Si PV cell

**Figure 12:**
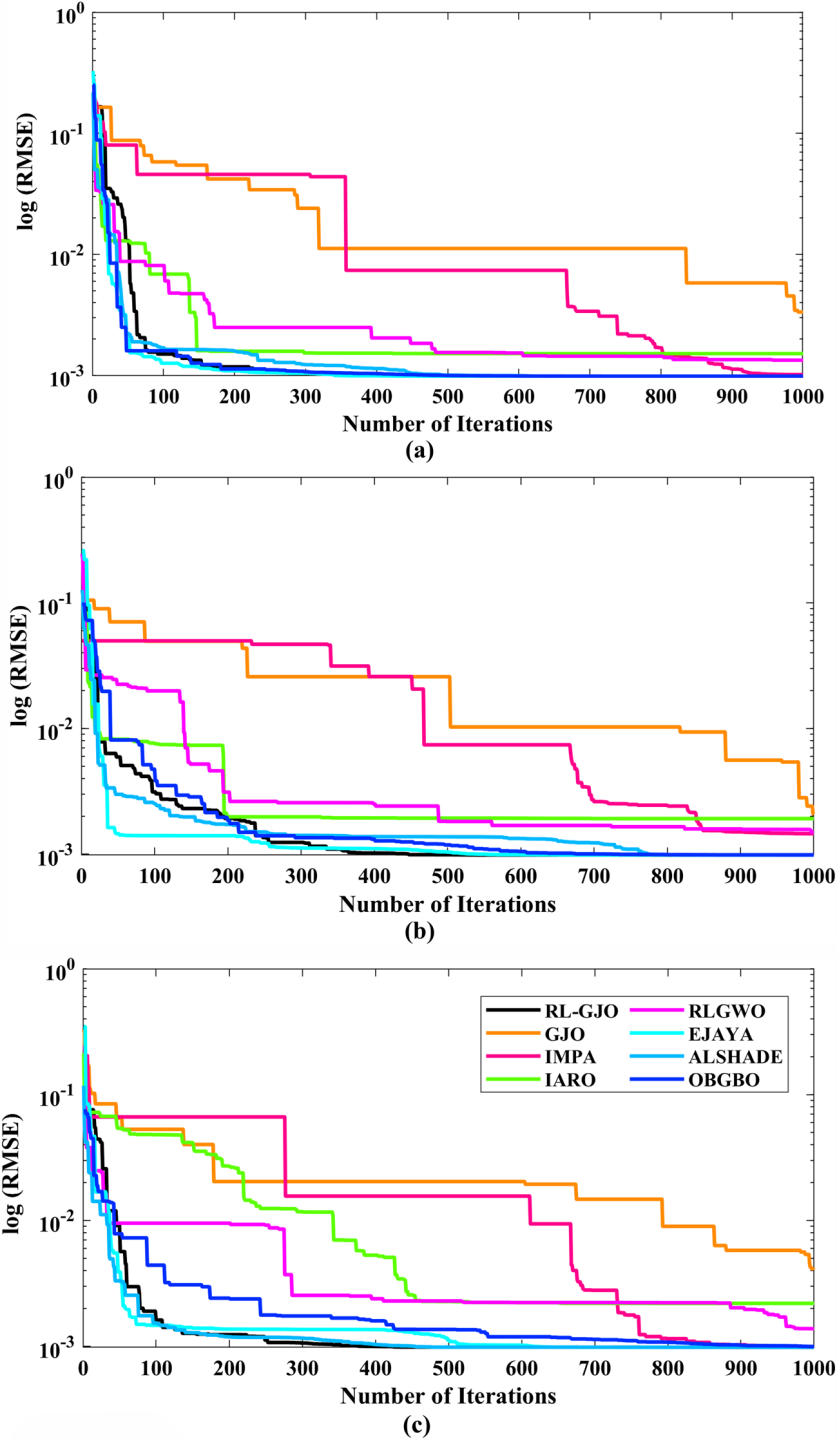
Convergence curves (Case 1); (**a**) SDM, (**b**) DDM, (**c**) TDM.

**Figure 13:**
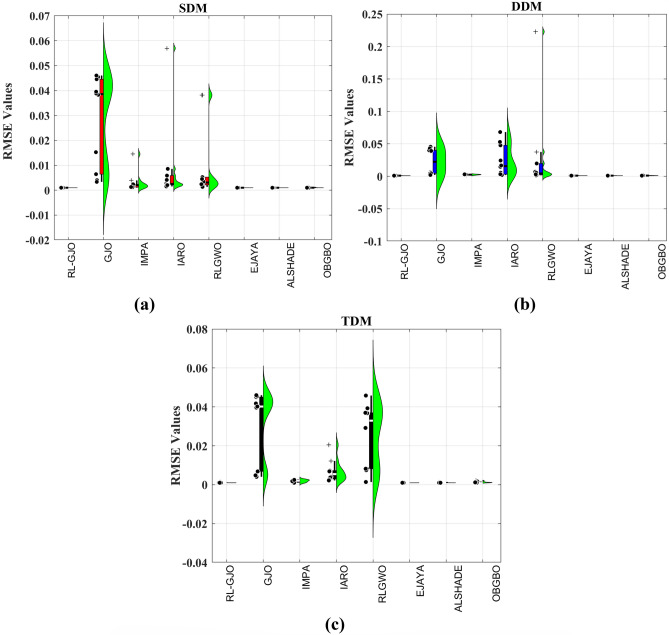
Boxplots (Case 1); (**a**) SDM, (**b**) DDM, (**c**) TDM.

This section delves into the outcomes generated by all the algorithms, including the newly proposed RL-GJO. Notably, the reliability and efficacy of RL-GJO exhibit enhancements when compared to the fundamental version of the GJO. Tables 9, 10 and 11 present the parameter results derived from each algorithm following 30 individual runs for the SDM, DDM, and TDM, respectively. Table 9 reveals that the RMSE obtained by the ALSHADE, OBGBO, and RL-GJO is remarkably similar, specifically at 9.8602E−04. However, this value surpasses the performance of all the other algorithms in the selection. In Table 10, it is observed that the RMSE value for the DDM, as obtained by the proposed RL-GJO, significantly outperforms all other algorithms, registering a value of 9.8255E−04. Similarly, Table 11 demonstrates that the RMSE value for the TDM achieved by RL-GJO is notably superior to all other algorithms, standing at 9.8085E−04. Even though ALSHADE and OBGBO results are equal to RL-GJO in the SDM case, RL-GJO exhibits superior reliability and convergence speed across SDM, DDM, and TDM. The RL-GJO distinguishes itself through its fast convergence speed and runtime. Consequently, RL-GJO emerges as a robust tool for addressing parameter estimation challenges. The convergence curve (Fig. 12) illustrates that RL-GJO’s convergence speed surpasses that of all the selected algorithms, while the box plot (Fig. 13) demonstrates RL-GJO’s higher reliability, denoted by its minimal STD, in comparison to other algorithms. Boxplots are invaluable due to their ability to represent data distributions briefly. This visualization aids in identifying outliers, which is crucial in parameter estimation as they can significantly impact model accuracy. Boxplots also facilitate the comparison of distributions across different datasets or scenarios, highlighting differences in variability, central tendencies, and the presence of extreme values. This comparative aspect is critical for choosing the most suitable model or adjusting existing models. Additionally, boxplots indicate data symmetry and skewness, informing the model selection process. Their robustness against outliers, deriving from their focus on medians and quartiles, makes them a reliable tool in parameter estimation, ensuring extreme values do not unduly influence data summaries. In Tables 9, 10 and 11, symbols like ‘+’ indicate that RL-GJO outperforms other algorithms, ‘−’ indicates a comparatively poorer performance, and ‘=’ signifies an equivalent performance. Furthermore, it is evident from Tables 9, 10 and 11 and Figs. 12 and 13 that, the performance of the original GJO ranks lower than RL-GJO. The proposed RL-GJO stands first, followed by ALSHADE, EJAYA, OBGBO, IMPA, RLGWO, IARO, and GJO. For a more detailed statistical analysis of all algorithms in estimating solar cell parameters, please refer to the subsequent section of this paper. The paper also explores statistical test analyses to substantiate the superiority of RL-GJO over other algorithms. Boldface entries in all tables denote the best results.

**Table 9:** Optimized 5-parameters by selected algorithms for RTC France Si PV cell.

Algorithm	${I}_{ph}$(A)	${I}_{sd}$(A)	${R}_{se}$(Ω)	${R}_{sh}$(Ω)	$a$	$RMSE$	*sign*
**RL-GJO**	**0.7608**	**3.23E−07**	**0.0364**	**53.72**	**1.4812**	**9.8602E−04**	
GJO	0.7640	7.47E−07	0.0321	35.66	1.5720	3.3403E−03	+
IMPA	0.7611	2.97E−07	0.0366	48.56	1.4730	1.0153E−03	+
IARO	0.7597	2.28E−07	0.0377	59.29	1.4467	1.5139E−03	+
RLGWO	0.7597	3.73E−07	0.0362	84.50	1.4956	1.3306E−03	+
**EJAYA**	**0.7608**	**3.23E−07**	**0.0364**	**53.72**	**1.4812**	**9.8602E−04**	=
**ALSHADE**	**0.7608**	**3.23E−07**	**0.0364**	**53.72**	**1.4812**	**9.8602E−04**	=
**OBGBO**	**0.7608**	**3.23E−07**	**0.0364**	**53.72**	**1.4812**	**9.8602E−04**	=

**Table 10:** Optimized 7-parameters by selected algorithms for RTC France Si PV cell.

Algorithm	${I}_{ph}$(A)	${I}_{sd1}$(A)	${R}_{se}$(Ω)	${R}_{sh}$(Ω)	${a}_{1}$	${I}_{sd2}$(A)	${a}_{2}$	$RSME$	*sign*
**RL-GJO**	**0.7608**	**2.16E−07**	**0.0368**	**55.76**	**1.4472**	**8.39E−07**	**2.0000**	**9.8255E−04**	
GJO	0.7600	0.00E + 00	0.0368	84.79	1.5113	2.89E−07	1.4693	2.0603E−03	+
IMPA	0.7609	1.91E−08	0.0341	65.06	2.0000	5.46E−07	1.5363	1.4538E−03	+
IARO	0.7613	5.09E−07	0.0332	67.88	1.9620	5.69E−07	1.5455	1.9164E−03	+
RLGWO	0.7599	8.88E−09	0.0345	99.14	1.8258	5.36E−07	1.5338	1.4993E−03	+
EJAYA	0.7608	2.07E−07	0.0368	55.50	1.4442	8.19E−07	1.9687	9.8350E−04	+
ALSHADE	0.7608	2.00E−07	0.0369	56.14	1.4407	9.87E−07	2.0000	9.8292E−04	+
OBGBO	0.7608	3.24E−07	0.0364	53.94	1.4815	9.13E−10	1.8833	9.8610E−04	+

**Table 11:** Optimized 9-parameters by selected algorithms for RTC France Si PV cell.

Algorithm	${I}_{ph}$(A)	${I}_{sd1}$(A)	${R}_{se}$(Ω)	${R}_{sh}$(Ω)	${a}_{1}$	${I}_{sd2}$(A)	${a}_{2}$	${I}_{sd3}$(A)	${a}_{3}$	$RSME$	*sign*
**RL-GJO**	**0.7608**	**2.30E−07**	**0.0366**	**55.21**	**1.9986**	**2.66E−07**	**1.4643**	**9.87E−07**	**2.4837**	**9.8085E−04**	
GJO	0.7606	9.35E−07	0.0295	78.25	1.5974	0.00E + 00	1.0690	5.60E−07	2.5256	4.0595E−03	+
IMPA	0.7609	4.29E−07	0.0367	52.45	1.9985	2.54E−07	1.4603	1.57E−07	3.8437	9.8792E−04	+
IARO	0.7615	1.74E−07	0.0396	42.28	1.4212	1.00E−21	1.3548	5.21E−07	3.4090	2.1938E−03	+
RLGWO	0.7596	3.40E−07	0.0353	93.87	1.5164	1.07E−07	1.5083	6.58E−07	4.4471	1.3802E−03	+
EJAYA	0.7608	1.98E−07	0.0369	56.53	1.4398	9.15E−07	2.0000	1.00E−06	2.8500	9.8167E−04	+
ALSHADE	0.7608	8.31E−07	0.0368	56.02	2.0000	2.18E−07	1.4479	9.68E−07	5.0000	9.8233E−04	+
OBGBO	0.7607	8.54E−09	0.0361	56.96	1.9832	3.43E−07	1.4874	1.00E−06	3.9486	9.9554E−04	+

**Figure 14:**
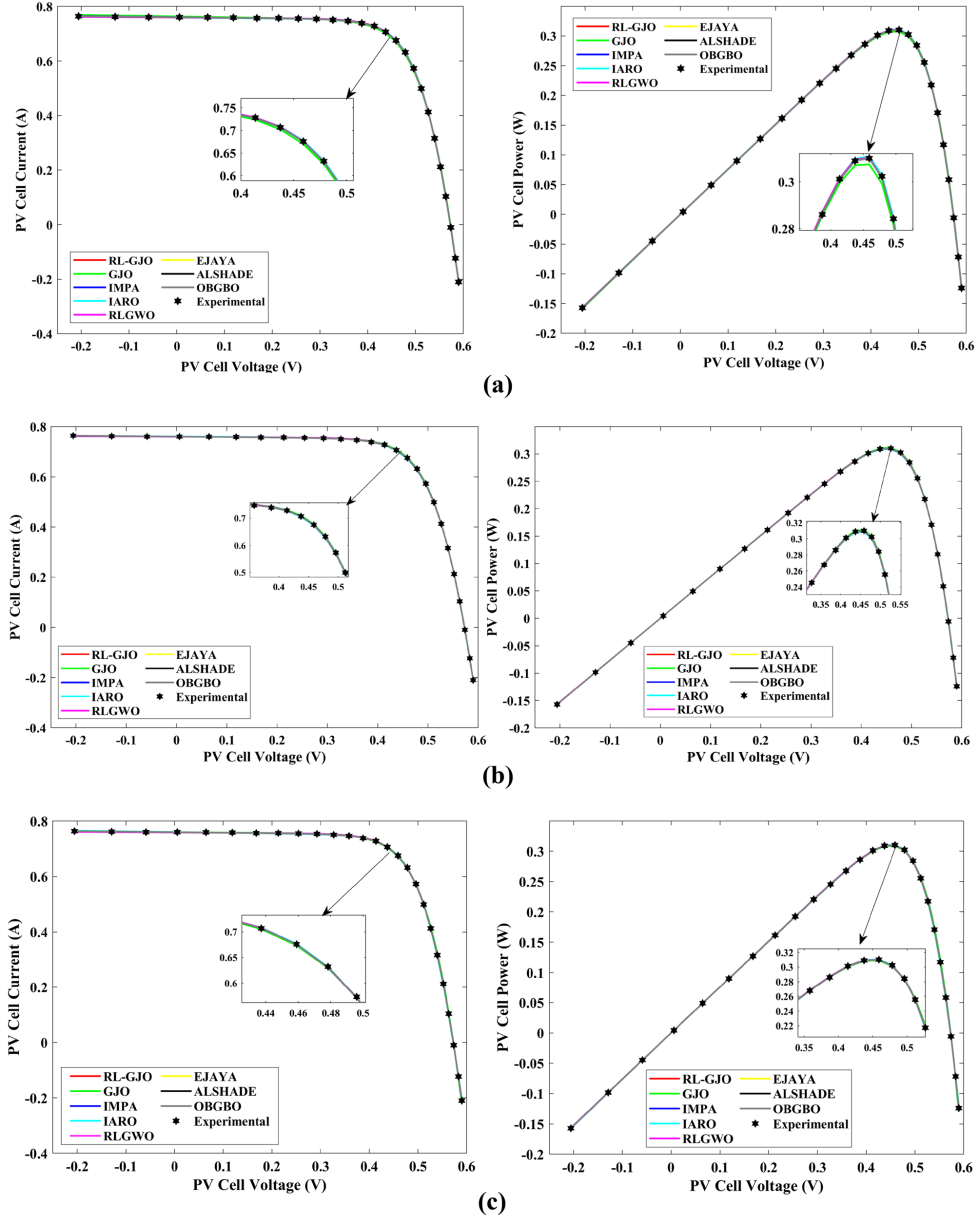
I–V and P–V Characteristics (Case 1); (**a**) SDM, (**b**) DDM, (**c**) TDM.

In addition to the outcomes discussed earlier, an important visual representation of the I–V and P–V characteristic of the RTC Si solar cell, estimated through the proposed RL-GJO for the SDM, DDM, and TDM, is provided in Fig. 14. This figure serves as a compelling illustration of the algorithm’s performance in modelling the behaviour of the solar cell. Upon careful examination of Fig. 14, it becomes evident that the estimated data points closely align with the experimental data points. This alignment showcases a remarkable level of correspondence between the estimated I–V characteristic and the actual behaviour of the RTC Si solar cell. Such a close match between estimated and experimental data is a strong indicator of the efficacy and superiority of the RL-GJO in comparison to other algorithms explored in this study. In essence, Fig. 14 provides tangible evidence of how well RL-GJO captures the intricate details of the I–V characteristic of the solar cell, thus emphasizing its efficiency and effectiveness in the realm of parameter estimation when compared to competing algorithms.

Tables 12, 13 and 14 provide a comprehensive breakdown of the AE, RE, MBE, and R^2^ values for all the data samples obtained through the utilization of the proposed RL-GJO. These tables offer valuable insights into the accuracy and reliability of the algorithm across different scenarios. Examining Table 12, it is observed that the AE values consistently fall below the threshold of 2.51E−03, while the RE, MBE, and R^2^ stay within the confines of 1.25E−01, 2.42E−07, and 0.9822. These metrics are indicative of the algorithm’s exceptional ability to model and predict the behaviour of the SDM accurately. Moving on to Table 13, it is found that the AE values for this dataset are all below 2.54E−03, and the RE, MBE, and R^2^ are below 1.2E−01, 2.49E−07, and 0.9845. These values underscore the algorithm’s strong performance in capturing the characteristics of the DDM effectively. Similarly, Table 14 reveals that for the TDM dataset, the AE values consistently stay below 2.5E−03, and the RE, MBE, and R^2^ are below 1.26E−01, 2.41E−07, and 0.9829. These statistics underscore the algorithm’s remarkable capacity to extract the actual characteristics of the TDM accurately. When we look at the average values across these datasets, it is found that for the SDM, the average values of AE, RE, MBE, and R^2^ are 8.28E−04, 4.74E−03, 3.74E−08, and 0.9993, respectively. Similarly, for the DDM, the average values of AE, RE, MBE, and R^2^ are 8.17E−04, 4.45E−03, 3.71E−08, and 0.9994, respectively. Finally, for the TDM, the average values of AE, RE, MBE, and R^2^ are 8.19E−04, 4.65E−03, 3.70E−08, and 0.9993, respectively. These average values further reinforce the conclusion that the proposed RL-GJO excels in extracting and representing the true characteristics of SDM, DDM, and TDM datasets accurately. In summary, Tables 12, 13 and 14 and the calculated averages collectively emphasize the impressive performance of the RL-GJO in capturing the behaviours of SDM, DDM, and TDM datasets, thereby underlining its effectiveness and reliability in the realm of parameter estimation.

**Table 12:** Different evaluation metrics of the Case 1 (SDM).

Data points	${V}_{ex}$(V)	${I}_{ex}$(A)	${I}_{es}$(A)	${P}_{ex}$(W)	${P}_{es}$(W)	$AE (A)$	$RE$	$MBE$	${R}^{2}$
1	− 0.2057	0.7640	0.7641	− 0.1572	− 0.1572	8.77E−05	− 1.15E−04	2.96E−10	1.0000
2	− 0.1291	0.7620	0.7627	− 0.0985	− 0.0984	6.63E−04	− 8.70E−04	1.69E−08	1.0000
3	− 0.0588	0.7605	0.7614	− 0.0448	− 0.0447	8.55E−04	− 1.12E−03	2.81E−08	1.0000
4	0.0057	0.7605	0.7602	0.0043	0.0043	3.46E−04	4.55E−04	4.60E−09	1.0000
5	0.0646	0.7600	0.7591	0.0490	0.0491	9.45E−04	1.24E−03	3.43E−08	1.0000
6	0.1185	0.7590	0.7580	0.0898	0.0899	9.58E−04	1.26E−03	3.53E−08	1.0000
7	0.1678	0.7570	0.7571	0.1270	0.1270	9.17E−05	− 1.21E−04	3.23E−10	1.0000
8	0.2132	0.7570	0.7561	0.1612	0.1614	8.59E−04	1.13E−03	2.84E−08	1.0000
9	0.2545	0.7555	0.7551	0.1922	0.1923	4.13E−04	5.47E−04	6.56E−09	1.0000
10	0.2924	0.7540	0.7537	0.2204	0.2205	3.36E−04	4.46E−04	4.35E−09	1.0000
11	0.3269	0.7505	0.7514	0.2456	0.2453	8.91E−04	− 1.19E−03	3.05E−08	1.0000
12	0.3585	0.7465	0.7474	0.2679	0.2676	8.54E−04	− 1.14E−03	2.80E−08	1.0000
13	0.3873	0.7385	0.7401	0.2866	0.2860	1.62E−03	− 2.19E−03	1.01E−07	1.0000
14	0.4137	0.7280	0.7274	0.3009	0.3012	6.18E−04	8.49E−04	1.47E−08	1.0000
15	0.4373	0.7065	0.7070	0.3092	0.3090	4.73E−04	− 6.69E−04	8.59E−09	1.0000
16	0.4590	0.6755	0.6753	0.3100	0.3101	2.20E−04	3.25E−04	1.86E−09	1.0000
17	0.4784	0.6320	0.6308	0.3018	0.3023	1.24E−03	1.96E−03	5.93E−08	1.0000
18	0.4960	0.5730	0.5719	0.2837	0.2842	1.07E−03	1.87E−03	4.42E−08	1.0000
19	0.5119	0.4990	0.4996	0.2557	0.2554	6.07E−04	− 1.22E−03	1.42E−08	1.0000
20	0.5265	0.4130	0.4136	0.2178	0.2174	6.49E−04	− 1.57E−03	1.62E−08	1.0000
21	0.5398	0.3165	0.3175	0.1714	0.1708	1.01E−03	− 3.19E−03	3.92E−08	1.0000
22	0.5521	0.2120	0.2122	0.1171	0.1170	1.55E−04	− 7.31E−04	9.23E−10	1.0000
23	0.5633	0.1035	0.1023	0.0576	0.0583	1.25E−03	1.21E−02	6.00E−08	0.9998
24	0.5736	− 0.0100	− 0.0087	− 0.0050	− 0.0057	1.28E−03	1.28E−01	6.33E−08	0.9822
25	0.5833	− 0.1230	− 0.1255	− 0.0732	− 0.0717	2.51E−03	− 2.04E−02	2.42E−07	0.9996
26	0.5900	− 0.2100	− 0.2085	− 0.1230	− 0.1239	1.53E−03	7.27E−03	8.98E−08	0.9999
Average values						8.28E−04	4.74E−03	3.74E−08	0.9993

**Table 13:** Different evaluation metrics of the Case 1 (DDM).

Data points	${V}_{ex}$(V)	${I}_{ex}$(A)	${I}_{es}$(A)	${P}_{ex}$(W)	${P}_{es}$(W)	$AE (A)$	$RE$	$MBE$	${R}^{2}$
1	− 0.2057	0.7640	0.7640	− 0.1571	− 0.1572	3.13E−05	4.10E−05	3.78E−11	1.0000
2	− 0.1291	0.7620	0.7626	− 0.0985	− 0.0984	5.96E−04	− 7.82E−04	1.37E−08	1.0000
3	− 0.0588	0.7605	0.7613	− 0.0448	− 0.0447	8.36E−04	− 1.10E−03	2.69E−08	1.0000
4	0.0057	0.7605	0.7602	0.0043	0.0043	3.23E−04	4.24E−04	4.00E−09	1.0000
5	0.0646	0.7600	0.7591	0.0490	0.0491	8.84E−04	1.16E−03	3.00E−08	1.0000
6	0.1185	0.7590	0.7581	0.0898	0.0899	8.66E−04	1.14E−03	2.88E−08	1.0000
7	0.1678	0.7570	0.7572	0.1271	0.1270	2.04E−04	− 2.70E−04	1.60E−09	1.0000
8	0.2132	0.7570	0.7563	0.1612	0.1614	7.40E−04	9.77E−04	2.10E−08	1.0000
9	0.2545	0.7555	0.7552	0.1922	0.1923	3.07E−04	4.06E−04	3.62E−09	1.0000
10	0.2924	0.7540	0.7537	0.2204	0.2205	2.65E−04	3.52E−04	2.71E−09	1.0000
11	0.3269	0.7505	0.7514	0.2456	0.2453	9.05E−04	− 1.21E−03	3.15E−08	1.0000
12	0.3585	0.7465	0.7473	0.2679	0.2676	8.00E−04	− 1.07E−03	2.46E−08	1.0000
13	0.3873	0.7385	0.7400	0.2866	0.2860	1.50E−03	− 2.03E−03	8.67E−08	1.0000
14	0.4137	0.7280	0.7272	0.3009	0.3012	7.66E−04	1.05E−03	2.26E−08	1.0000
15	0.4373	0.7065	0.7068	0.3091	0.3090	3.37E−04	− 4.78E−04	4.38E−09	1.0000
16	0.4590	0.6755	0.6752	0.3099	0.3101	2.97E−04	4.40E−04	3.40E−09	1.0000
17	0.4784	0.6320	0.6308	0.3018	0.3023	1.24E−03	1.96E−03	5.91E−08	1.0000
18	0.4960	0.5730	0.5720	0.2837	0.2842	1.00E−03	1.74E−03	3.84E−08	1.0000
19	0.5119	0.4990	0.4997	0.2558	0.2554	7.14E−04	− 1.43E−03	1.96E−08	1.0000
20	0.5265	0.4130	0.4137	0.2178	0.2174	7.38E−04	− 1.79E−03	2.10E−08	1.0000
21	0.5398	0.3165	0.3175	0.1714	0.1708	1.04E−03	− 3.30E−03	4.19E−08	1.0000
22	0.5521	0.2120	0.2121	0.1171	0.1170	1.12E−04	− 5.28E−04	4.82E−10	1.0000
23	0.5633	0.1035	0.1021	0.0575	0.0583	1.35E−03	1.31E−02	7.04E−08	0.9998
24	0.5736	− 0.0100	− 0.0088	− 0.0050	− 0.0057	1.20E−03	1.20E−01	5.51E−08	0.9845
25	0.5833	− 0.1230	− 0.1255	− 0.0732	− 0.0717	2.54E−03	− 2.07E−02	2.49E−07	0.9995
26	0.5900	− 0.2100	− 0.2084	− 0.1229	− 0.1239	1.65E−03	7.86E−03	1.05E−07	0.9999
Average values						8.17E−04	4.45E−03	3.71E−08	0.9994

**Table 14:** Different evaluation metrics of the Case 1 (TDM).

Data points	${V}_{ex}$(V)	${I}_{ex}$(A)	${I}_{es}$(A)	${P}_{ex}$(W)	${P}_{es}$(W)	$AE (A)$	$RE$	$MBE$	${R}^{2}$
1	− 0.2057	0.7640	0.7640	− 0.1572	− 0.1572	2.42E−05	− 3.17E−05	2.26E−11	1.0000
2	− 0.1291	0.7620	0.7626	− 0.0985	− 0.0984	6.38E−04	− 8.37E−04	1.57E−08	1.0000
3	− 0.0588	0.7605	0.7614	− 0.0448	− 0.0447	8.65E−04	− 1.14E−03	2.88E−08	1.0000
4	0.0057	0.7605	0.7602	0.0043	0.0043	3.05E−04	4.01E−04	3.57E−09	1.0000
5	0.0646	0.7600	0.7591	0.0490	0.0491	8.77E−04	1.15E−03	2.96E−08	1.0000
6	0.1185	0.7590	0.7581	0.0898	0.0899	8.69E−04	1.14E−03	2.90E−08	1.0000
7	0.1678	0.7570	0.7572	0.1271	0.1270	1.93E−04	− 2.56E−04	1.44E−09	1.0000
8	0.2132	0.7570	0.7562	0.1612	0.1614	7.55E−04	9.97E−04	2.19E−08	1.0000
9	0.2545	0.7555	0.7552	0.1922	0.1923	3.22E−04	4.26E−04	3.99E−09	1.0000
10	0.2924	0.7540	0.7537	0.2204	0.2205	2.74E−04	3.63E−04	2.88E−09	1.0000
11	0.3269	0.7505	0.7514	0.2456	0.2453	9.11E−04	− 1.21E−03	3.19E−08	1.0000
12	0.3585	0.7465	0.7473	0.2679	0.2676	8.24E−04	− 1.10E−03	2.61E−08	1.0000
13	0.3873	0.7385	0.7400	0.2866	0.2860	1.54E−03	− 2.09E−03	9.15E−08	1.0000
14	0.4137	0.7280	0.7273	0.3009	0.3012	7.18E−04	9.87E−04	1.99E−08	1.0000
15	0.4373	0.7065	0.7069	0.3091	0.3090	3.77E−04	− 5.33E−04	5.46E−09	1.0000
16	0.4590	0.6755	0.6752	0.3099	0.3101	2.82E−04	4.18E−04	3.07E−09	1.0000
17	0.4784	0.6320	0.6307	0.3017	0.3023	1.26E−03	1.99E−03	6.07E−08	1.0000
18	0.4960	0.5730	0.5720	0.2837	0.2842	1.04E−03	1.82E−03	4.17E−08	1.0000
19	0.5119	0.4990	0.4997	0.2558	0.2554	6.63E−04	− 1.33E−03	1.69E−08	1.0000
20	0.5265	0.4130	0.4137	0.2178	0.2174	7.00E−04	− 1.69E−03	1.88E−08	1.0000
21	0.5398	0.3165	0.3175	0.1714	0.1708	1.04E−03	− 3.27E−03	4.12E−08	1.0000
22	0.5521	0.2120	0.2121	0.1171	0.1170	1.42E−04	− 6.71E−04	7.77E−10	1.0000
23	0.5633	0.1035	0.1022	0.0576	0.0583	1.29E−03	1.25E−02	6.41E−08	0.9998
24	0.5736	− 0.0100	− 0.0087	− 0.0050	− 0.0057	1.26E−03	1.26E−01	6.07E−08	0.9829
25	0.5833	− 0.1230	− 0.1255	− 0.0732	− 0.0717	2.50E−03	− 2.03E−02	2.41E−07	0.9996
26	0.5900	− 0.2100	− 0.2084	− 0.1229	-0.1239	1.63E−03	7.75E−03	1.02E−07	0.9999
Average values						8.19E−04	4.65E−03	3.70E−08	0.9993

**Figure 15:**
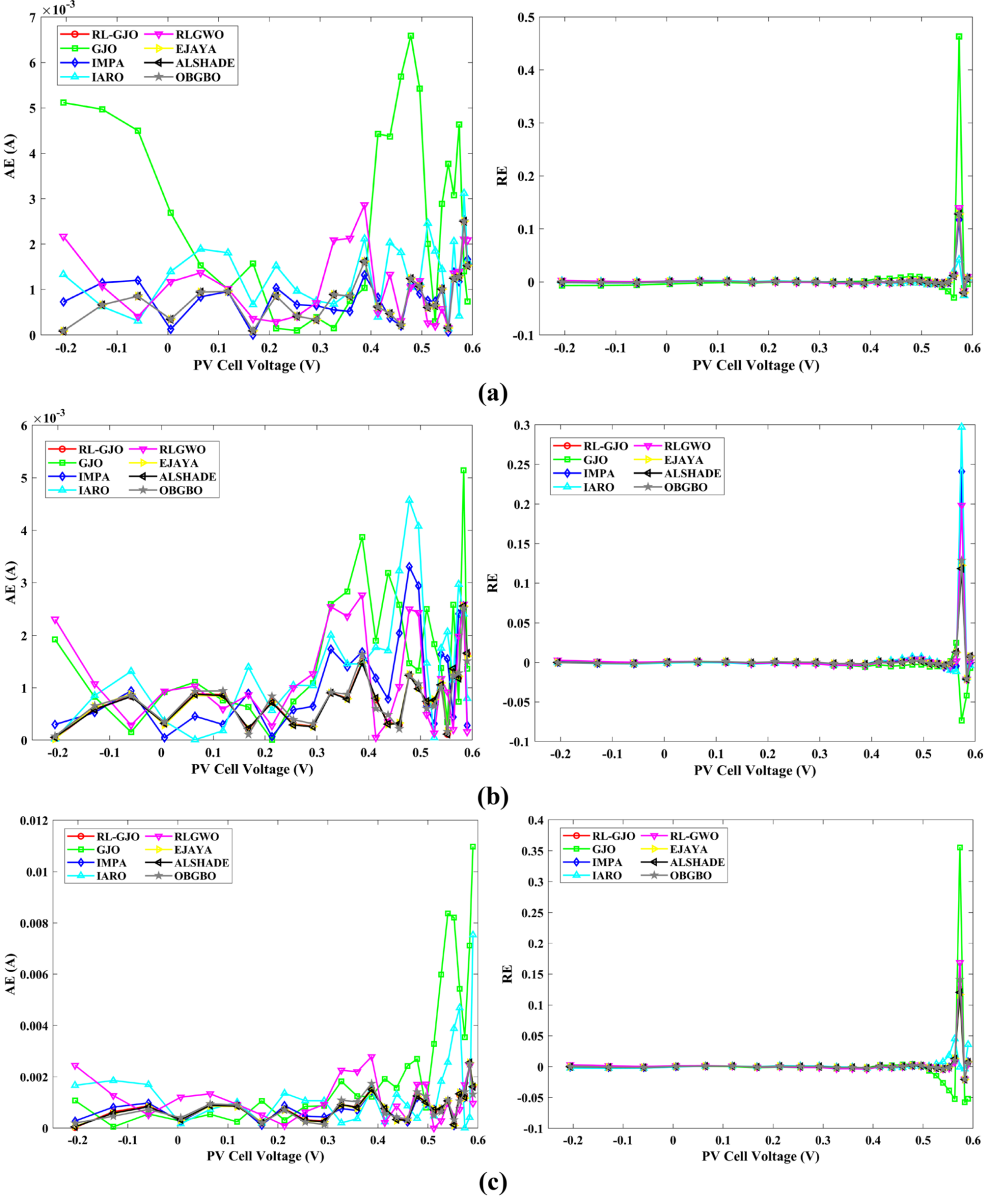
AE and RE Metrics (Case 1); (**a**) SDM, (**b**) DDM, (**c**) TDM.

The study has gathered RE and AE values from multiple algorithms. These values represent how well each algorithm’s predictions match the actual measured cell voltage. To make these results more comprehensible and visually informative, different plots are created. For each algorithm, there is a set of RE and IAE values corresponding to different measured cell voltages. Each algorithm is likely represented by a different line or set of data points on the graph, as illustrated in Fig. 15. Figure 15 can gain insights into how well each algorithm performs at different voltage levels. It allows for a more intuitive understanding of which algorithms perform better or worse under various conditions. This visual representation can help researchers and readers quickly identify trends and patterns in the algorithm’s performance.

#### Results of SDM/DDM/TDM of PVM752 GaAs thin-film PV cell

**Figure 16:**
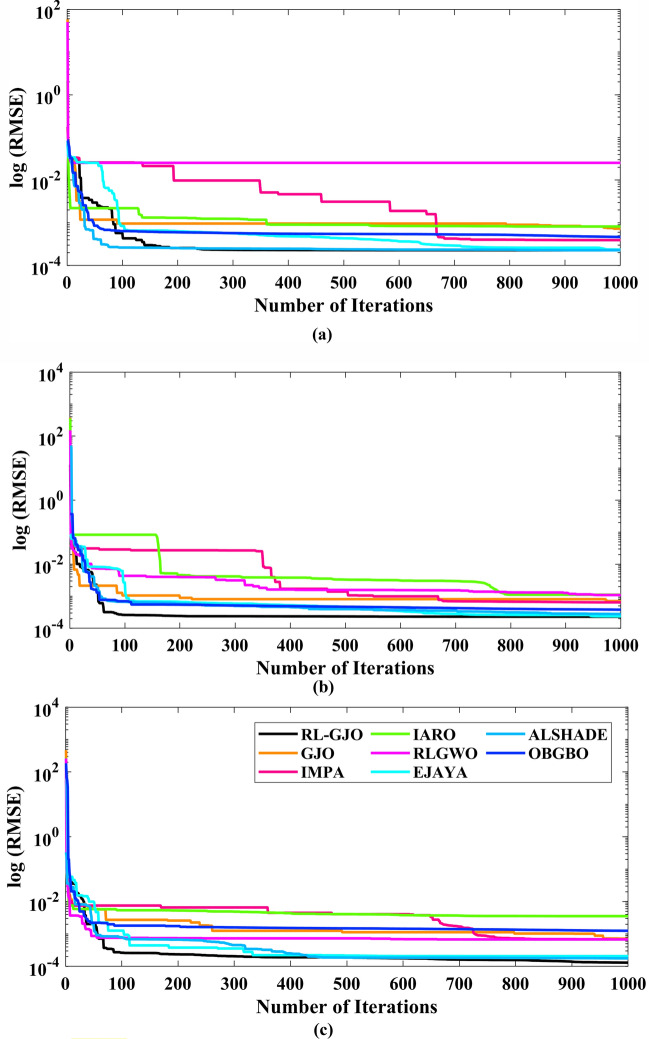
Convergence curves (Case 2); (**a**) SDM, (**b**) DDM, (**c**) TDM.

**Figure 17:**
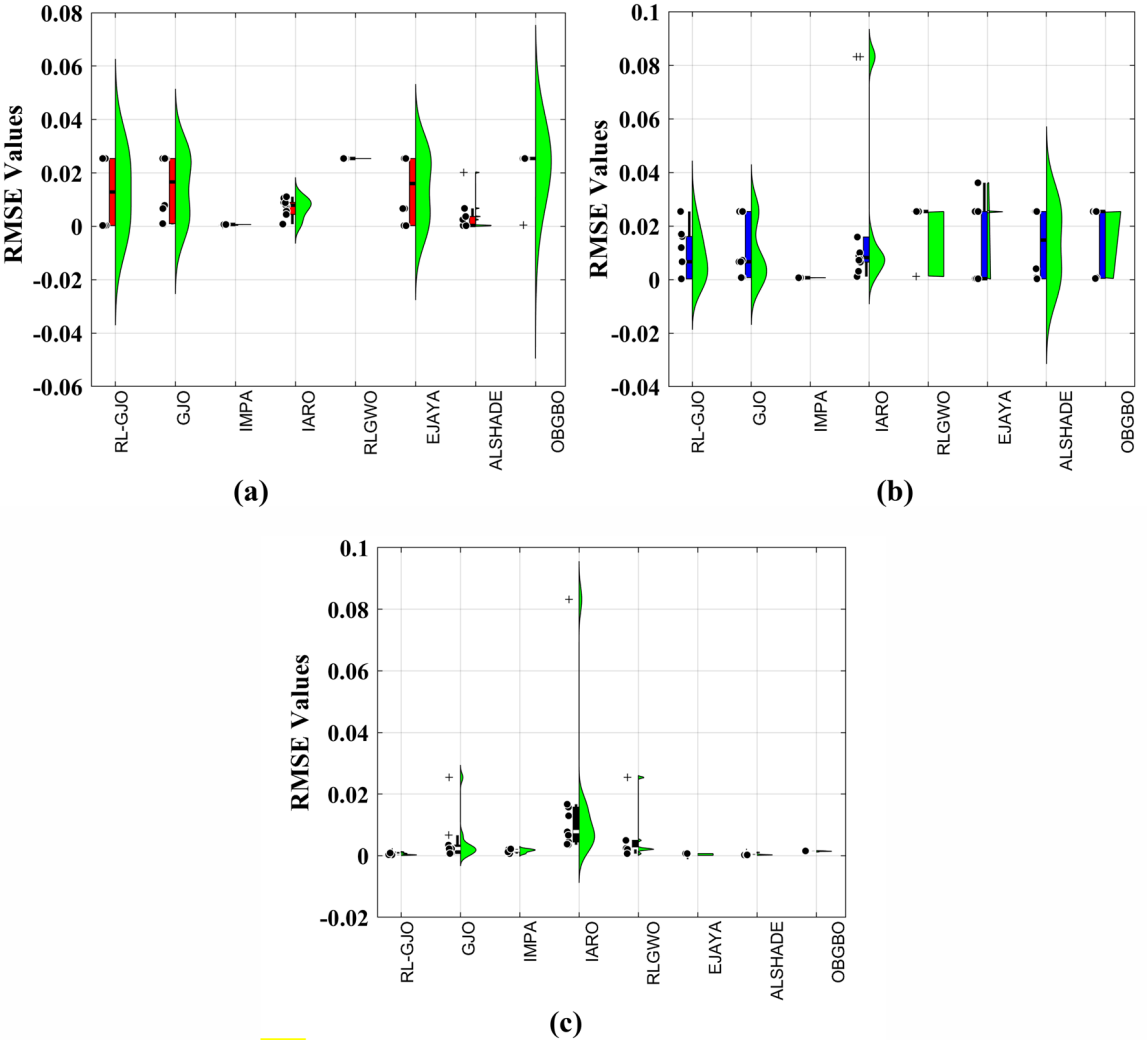
Boxplots (Case 2); (**a**) SDM, (**b**) DDM, (**c**) TDM.

Similar to the previous case, this section delves into the outcomes generated by all the algorithms, including the newly proposed RL-GJO. Notably, the reliability and efficacy of RL-GJO exhibit enhancements when compared to the fundamental version of the GJO. Tables 15, 16 and 17 present the parameter results derived from each algorithm following 30 individual runs for the SDM, DDM, and TDM, respectively. Table 15 reveals that the RMSE obtained by the ALSHADE and RL-GJO is remarkably similar, specifically at 2.2781E−04. However, this value surpasses the performance of all the other algorithms in the selection. In Table 16, it is observed that the RMSE value for the DDM, as obtained by the proposed RL-GJO, significantly outperforms all other algorithms, registering a value of 2.2969E−04. Similarly, Table 17 demonstrates that the RMSE value for the TDM achieved by RL-GJO is notably superior to all other algorithms, standing at 1.2991E−04. Even though ALSHADE results are equal to RL-GJO in the SDM case, RL-GJO exhibits superior reliability and convergence speed across SDM, DDM, and TDM. The convergence curve (Fig. 16) illustrates that RL-GJO’s convergence speed surpasses that of all the selected algorithms, while the box plot (Fig. 17) demonstrates RL-GJO’s higher reliability, denoted by its minimal STD, in comparison to other algorithms. In Tables 15, 16 and 17, symbols like ‘+’ indicate that RL-GJO outperforms other algorithms, ‘−’ indicates a comparatively poorer performance, and ‘=’ signifies an equivalent performance. Furthermore, it is evident from Tables 15, 16 and 17 and Figs. 16 and 17, the performance of the original GJO ranks lower than RL-GJO. The proposed RL-GJO stands first, followed by ALSHADE, EJAYA, IMPA, OBGBO, RLGWO, GJO, and IARO. The paper also explores statistical test analyses to substantiate the superiority of RL-GJO over other algorithms.

**Table 15:** Optimized 5-parameters by selected algorithms for PVM752 GaAs PV cell.

Algorithm	${I}_{ph}$(A)	${I}_{sd}$(A)	${R}_{se}$(Ω)	${R}_{sh}$(Ω)	$a$	$RMSE$	*sign*
**RL-GJO**	**0.1001**	**3.81E−12**	**0.6603**	**609.18**	**1.6161**	**2.2781E−04**	
GJO	0.1002	3.81E−10	0.5209	801.52	2.0000	7.2242E−04	+
IMPA	0.1000	4.35E−11	0.5968	928.22	1.7986	3.9376E−04	+
IARO	0.1002	3.84E−10	0.5016	1000.00	2.0000	8.1996E−04	+
RLGWO	0.1138	0.00E + 00	0.0000	14.59	1.0046	2.5400E−02	+
EJAYA	0.1000	6.17E−12	0.6494	686.40	1.6493	2.3590E−04	=
**ALSHADE**	**0.1001**	**3.67E−12**	**0.6612**	**604.73**	**1.6138**	**2.2781E−04**	=
OBGBO	0.1000	7.81E−11	0.5775	999.96	1.8488	4.6623E−04	=

**Table 16:** Optimized 7-parameters by selected algorithms for PVM752 GaAs PV cell.

Algorithm	${I}_{ph}$(A)	${I}_{sd1}$(A)	${R}_{se}$(Ω)	${R}_{sh}$(Ω)	${a}_{1}$	${I}_{sd2}$(A)	${a}_{2}$	$RSME$	*sign*
**RL-GJO**	**0.1000**	**2.33E−13**	**0.6587**	**680.09**	**1.5405**	**4.62E−12**	**1.6445**	**2.2969E−04**	
GJO	0.1002	3.82E−10	0.5218	950.14	2.0000	0.00E+00	1.6956	7.0489E−04	+
IMPA	0.1001	2.79E−12	0.5382	1000.00	1.7318	3.26E−10	2.0000	6.4578E−04	+
IARO	0.0988	2.50E−11	0.5760	719.95	1.8118	2.50E−11	1.8118	1.1152E−03	+
RLGWO	0.1019	0.00E+00	0.6437	160.22	1.0727	2.68E−12	1.5947	1.0885E−03	+
EJAYA	0.1000	0.00E+00	0.6488	682.10	2.0000	6.02E−12	1.6476	2.3688E−04	+
ALSHADE	0.0999	1.38E−11	0.6313	999.02	1.7074	0.00E+00	1.0004	2.7803E−04	+
OBGBO	0.1000	3.98E−11	0.5987	999.92	1.7911	0.00E+00	1.5297	3.8134E−04	+

**Table 17:** Optimized 9-parameters by selected algorithms for PVM752 GaAs PV cell.

Algorithm	${I}_{ph}$(A)	${I}_{sd1}$(A)	${R}_{se}$(Ω)	${R}_{sh}$(Ω)	${a}_{1}$	${I}_{sd2}$(A)	${a}_{2}$	${I}_{sd3}$(A)	${a}_{3}$	$RSME$	*sign*
**RL-GJO**	**0.1000**	**4.06E−14**	**0.7098**	**999.94**	**1.3638**	**6.27E−19**	**1.9870**	**2.72E−07**	**3.6746**	**1.2991E−04**	
GJO	0.1002	3.82E−10	0.5221	984.31	2.0000	0.00E+00	1.5341	0.00E+00	4.2363	7.0247E−04	+
IMPA	0.1002	2.10E−10	0.5362	1000.00	1.9415	6.54E−34	1.9700	6.12E−07	5.0000	6.7833E−04	+
IARO	0.1015	5.36E−11	0.7192	899.06	1.8793	4.89E−11	1.8789	8.99E−07	4.6523	3.5532E−03	+
RLGWO	0.1002	0.00E+00	0.5305	899.46	1.4736	3.12E−10	1.9795	0.00E+00	2.9893	6.7752E−04	+
EJAYA	0.1001	2.43E−14	0.6112	952.63	2.0000	1.61E−11	1.7208	1.00E−06	4.9941	2.0650E−04	+
ALSHADE	0.0999	0.00E+00	0.6968	994.29	1.9944	6.54E−15	1.2942	5.33E−10	2.1593	1.7704E−04	+
OBGBO	0.1004	0.00E+00	0.3717	999.98	1.2462	0.00E+00	1.6447	9.17E−09	2.3921	1.2525E−03	+

**Figure 18:**
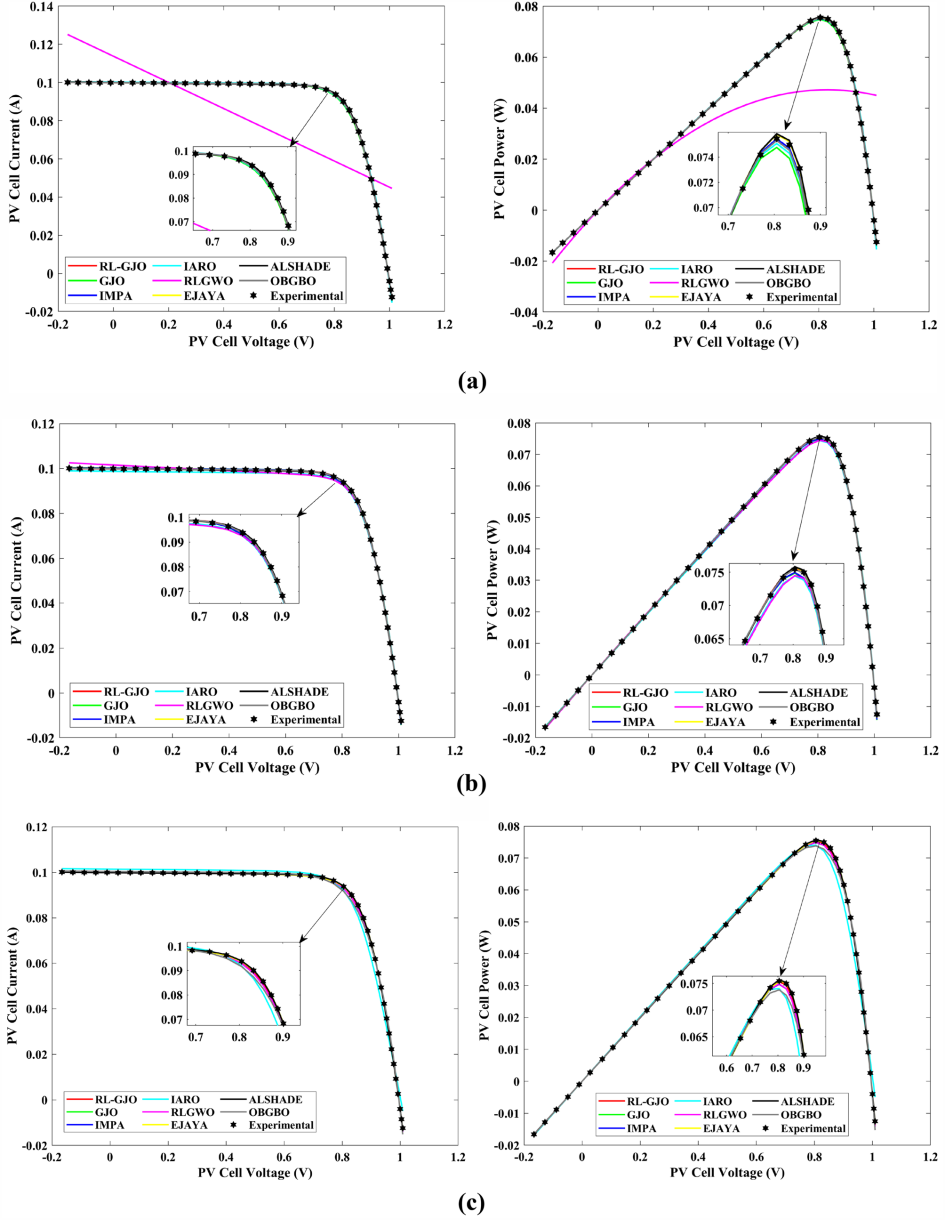
I–V and P–V Characteristics (Case 2); (**a**) SDM, (**b**) DDM, (**c**) TDM.

In addition to the outcomes discussed earlier, an important visual representation of the I–V and P–V characteristics of the PVM752 GaAs thin-film cell, estimated through the proposed RL-GJO for the SDM, DDM, and TDM, is provided in Fig. 18. This figure serves as a compelling illustration of the algorithm’s performance in modelling the behaviour of the solar cell. Upon careful examination of Fig. 18, it becomes evident that the estimated data points closely align with the experimental data points. This alignment showcases a remarkable level of correspondence between the estimated I–V characteristic and the actual behaviour of the PVM752 GaAs thin-film cell. Such a close match between estimated and experimental data is a strong indicator of the efficacy and superiority of the RL-GJO in comparison to other algorithms explored in this study. In essence, Fig. 18 provides tangible evidence of how well RL-GJO captures the intricate details of the I–V and P–V characteristics of the solar cell, thus emphasizing its efficiency and effectiveness in the realm of parameter estimation when compared to competing algorithms.

Tables 18, 19 and 20 provide a comprehensive breakdown of the AE, RE, MBE, and R^2^ values for all the data samples obtained through the utilization of the proposed RL-GJO. These tables offer valuable insights into the accuracy and reliability of the algorithm across different scenarios. Examining Table 18, it is observed that the AE values consistently fall below the threshold of 4.62E−04, while the RE, MBE, and R^2^ stay within the confines of 5.02E−02, 8.34E−09, and 0.9852. These metrics are indicative of the algorithm’s exceptional ability to model and predict the behaviour of the SDM accurately. Moving on to Table 19, it is found that the AE values for this dataset are all below 5.54E−04, and the RE, MBE, and R^2^ are below 6.67E−02, 1.56E−08, and 0.9812. These values underscore the algorithm’s strong performance in capturing the characteristics of the DDM effectively. Similarly, Table 20 reveals that for the TDM dataset, the AE values consistently stay below 7.74E−04, and the RE, MBE, and R^2^ are below 9.18E−02, 2.95E−08, and 0.9849. These statistics underscore the algorithm’s remarkable capacity to extract the actual characteristics of the TDM accurately. When we look at the average values across these datasets, it is found that for the SDM, the average values of AE, RE, MBE, and R^2^ are 1.82E−04, 5.90E−03, 1.18E−09, and 0.9995, respectively. Similarly, for the DDM, the average values of AE, RE, MBE, and R^2^ are 2.09E−04, 8.09E−03, 1.83E−09, and 0.9993, respectively. Finally, for the TDM, the average values of AE, RE, MBE, and R^2^ are 2.60E−04, 1.17E−02, 3.18E−09, and 0.9988, respectively. These average values further reinforce the conclusion that the proposed RL-GJO excels in extracting and representing the true characteristics of SDM, DDM, and TDM datasets accurately. In summary, Tables 18, 19 and 20 and the calculated averages collectively emphasize the impressive performance of the RL-GJO in capturing the behaviours of SDM, DDM, and TDM datasets, thereby underlining its effectiveness and reliability in the realm of parameter estimation.

**Table 18:** Different evaluation metrics of the Case 2 (SDM).

Data points	${V}_{ex}$(V)	${I}_{ex}$(A)	${I}_{es}$(A)	${P}_{ex}$(W)	${P}_{es}$(W)	$AE (A)$	$RE$	$MBE$	${R}^{2}$
1	− 0.1659	0.1001	0.1002	− 0.0166	− 0.0166	1.29E−04	− 1.29E−03	3.79E−10	1.0000
2	− 0.1281	0.1000	0.1002	− 0.0128	− 0.0128	1.67E−04	− 1.67E−03	6.36E−10	1.0000
3	− 0.0888	0.0999	0.1001	− 0.0089	− 0.0089	2.03E−04	− 2.03E−03	9.35E−10	1.0000
4	− 0.0490	0.0999	0.1000	− 0.0049	− 0.0049	1.38E−04	− 1.38E−03	4.30E−10	1.0000
5	− 0.0102	0.0999	0.1000	− 0.0010	− 0.0010	7.38E−05	− 7.39E−04	1.24E−10	1.0000
6	0.0275	0.0998	0.0999	0.0027	0.0027	1.12E−04	− 1.12E−03	2.85E−10	1.0000
7	0.0695	0.0999	0.0998	0.0069	0.0069	5.70E−05	5.71E−04	7.39E−11	1.0000
8	0.1061	0.0998	0.0998	0.0106	0.0106	1.70E−05	1.70E−04	6.56E−12	1.0000
9	0.1460	0.0998	0.0997	0.0146	0.0146	8.25E−05	8.27E−04	1.55E−10	1.0000
10	0.1828	0.0997	0.0997	0.0182	0.0182	4.28E−05	4.29E−04	4.16E−11	1.0000
11	0.2230	0.0997	0.0996	0.0222	0.0222	1.09E−04	1.09E−03	2.69E−10	1.0000
12	0.2600	0.0996	0.0995	0.0259	0.0259	6.94E−05	6.97E−04	1.10E−10	1.0000
13	0.3001	0.0997	0.0995	0.0298	0.0299	2.35E−04	2.36E−03	1.26E−09	1.0000
14	0.3406	0.0996	0.0994	0.0339	0.0339	2.02E−04	2.03E−03	9.25E−10	1.0000
15	0.3789	0.0995	0.0993	0.0376	0.0377	1.65E−04	1.65E−03	6.16E−10	1.0000
16	0.4168	0.0994	0.0993	0.0414	0.0414	1.27E−04	1.28E−03	3.67E−10	1.0000
17	0.4583	0.0994	0.0992	0.0455	0.0456	1.96E−04	1.97E−03	8.72E−10	1.0000
18	0.4949	0.0993	0.0991	0.0491	0.0491	1.57E−04	1.59E−03	5.63E−10	1.0000
19	0.5370	0.0993	0.0991	0.0532	0.0533	2.31E−04	2.33E−03	1.22E−09	1.0000
20	0.5753	0.0992	0.0990	0.0570	0.0571	2.06E−04	2.07E−03	9.62E−10	1.0000
21	0.6123	0.0990	0.0989	0.0606	0.0606	9.37E−05	9.46E−04	1.99E−10	1.0000
22	0.6546	0.0988	0.0988	0.0646	0.0647	4.49E−05	4.55E−04	4.59E−11	1.0000
23	0.6918	0.0983	0.0985	0.0681	0.0680	2.11E−04	− 2.14E−03	1.01E−09	1.0000
24	0.7318	0.0977	0.0979	0.0717	0.0715	2.47E−04	− 2.53E−03	1.38E−09	1.0000
25	0.7702	0.0963	0.0967	0.0745	0.0742	3.96E−04	− 4.11E−03	3.57E−09	1.0000
26	0.8053	0.0937	0.0942	0.0758	0.0755	4.72E−04	− 5.04E−03	5.06E−09	1.0000
27	0.8329	0.0900	0.0904	0.0753	0.0750	4.08E−04	− 4.53E−03	3.78E−09	1.0000
28	0.8550	0.0855	0.0856	0.0732	0.0731	8.26E−05	− 9.66E−04	1.55E−10	1.0000
29	0.8738	0.0799	0.0799	0.0698	0.0698	3.87E−05	4.85E−04	3.41E−11	1.0000
30	0.8887	0.0743	0.0741	0.0658	0.0660	2.43E−04	3.27E−03	1.34E−09	1.0000
31	0.9016	0.0683	0.0682	0.0615	0.0616	1.34E−04	1.97E−03	4.10E−10	1.0000
32	0.9141	0.0618	0.0615	0.0562	0.0565	2.80E−04	4.53E−03	1.78E−09	1.0000
33	0.9248	0.0555	0.0552	0.0511	0.0513	2.98E−04	5.38E−03	2.02E−09	1.0000
34	0.9344	0.0493	0.0491	0.0458	0.0461	2.45E−04	4.97E−03	1.36E−09	1.0000
35	0.9445	0.0422	0.0422	0.0398	0.0399	3.36E−05	7.96E−04	2.57E−11	1.0000
36	0.9533	0.0357	0.0357	0.0340	0.0340	2.63E−06	− 7.36E−05	1.57E−13	1.0000
37	0.9618	0.0291	0.0291	0.0280	0.0280	1.31E−05	− 4.49E−04	3.87E−12	1.0000
38	0.9702	0.0222	0.0224	0.0217	0.0215	1.82E−04	− 8.18E−03	7.49E−10	0.9999
39	0.9778	0.0157	0.0161	0.0157	0.0154	3.69E−04	− 2.35E−02	3.09E−09	0.9994
40	0.9852	0.0092	0.0097	0.0095	0.0091	4.62E−04	− 5.02E−02	4.85E−09	0.9974
41	0.9926	0.0026	0.0029	0.0029	0.0026	3.09E−04	− 1.19E−01	2.18E−09	0.9852
42	0.9999	− 0.0040	− 0.0041	− 0.0041	− 0.0040	1.08E−04	− 2.71E−02	2.67E−10	0.9992
43	1.0046	− 0.0085	− 0.0085	− 0.0085	− 0.0085	3.05E−05	3.59E−03	2.11E−11	1.0000
44	1.0089	− 0.0124	− 0.0130	− 0.0131	− 0.0125	6.06E−04	− 4.88E−02	8.34E−09	0.9975
Average values						1.82E−04	− 5.90E−03	1.18E−09	0.9995

**Table 19:** Different evaluation metrics of the Case 2 (DDM).

Data points	${V}_{ex}$(V)	${I}_{ex}$(A)	${I}_{es}$(A)	${P}_{ex}$(W)	${P}_{es}$(W)	$AE (A)$	$RE$	$MBE$	${R}^{2}$
1	− 0.1659	0.1001	0.1000	− 0.0166	− 0.0166	9.48E−05	9.47E−04	2.04E−10	1.0000
2	− 0.1281	0.1000	0.1000	− 0.0128	− 0.0128	3.25E−05	3.25E−04	2.41E−11	1.0000
3	− 0.0888	0.0999	0.0999	− 0.0089	− 0.0089	2.82E−05	− 2.82E−04	1.80E−11	1.0000
4	− 0.0490	0.0999	0.0999	− 0.0049	− 0.0049	1.17E−05	1.17E−04	3.09E−12	1.0000
5	− 0.0102	0.0999	0.0998	− 0.0010	− 0.0010	5.05E−05	5.06E−04	5.80E−11	1.0000
6	0.0275	0.0998	0.0998	0.0027	0.0027	1.18E−05	− 1.18E−04	3.18E−12	1.0000
7	0.0695	0.0999	0.0998	0.0069	0.0069	1.30E−04	1.30E−03	3.86E−10	1.0000
8	0.1061	0.0998	0.0997	0.0106	0.0106	6.69E−05	6.70E−04	1.02E−10	1.0000
9	0.1460	0.0998	0.0997	0.0146	0.0146	1.07E−04	1.07E−03	2.59E−10	1.0000
10	0.1828	0.0997	0.0997	0.0182	0.0182	4.36E−05	4.37E−04	4.31E−11	1.0000
11	0.2230	0.0997	0.0996	0.0222	0.0222	8.38E−05	8.41E−04	1.60E−10	1.0000
12	0.2600	0.0996	0.0996	0.0259	0.0259	2.08E−05	2.09E−04	9.83E−12	1.0000
13	0.3001	0.0997	0.0995	0.0299	0.0299	1.61E−04	1.62E−03	5.89E−10	1.0000
14	0.3406	0.0996	0.0995	0.0339	0.0339	1.02E−04	1.02E−03	2.35E−10	1.0000
15	0.3789	0.0995	0.0995	0.0377	0.0377	4.01E−05	4.03E−04	3.65E−11	1.0000
16	0.4168	0.0994	0.0994	0.0414	0.0414	2.16E−05	− 2.18E−04	1.06E−11	1.0000
17	0.4583	0.0994	0.0994	0.0455	0.0456	2.11E−05	2.13E−04	1.01E−11	1.0000
18	0.4949	0.0993	0.0993	0.0492	0.0491	3.97E−05	− 4.00E−04	3.59E−11	1.0000
19	0.5370	0.0993	0.0993	0.0533	0.0533	9.77E−06	9.84E−05	2.17E−12	1.0000
20	0.5753	0.0992	0.0992	0.0571	0.0571	3.54E−05	− 3.57E−04	2.85E−11	1.0000
21	0.6123	0.0990	0.0992	0.0607	0.0606	1.61E−04	− 1.63E−03	5.89E−10	1.0000
22	0.6546	0.0988	0.0990	0.0648	0.0647	2.12E−04	− 2.15E−03	1.02E−09	1.0000
23	0.6918	0.0983	0.0987	0.0683	0.0680	4.48E−04	− 4.55E−03	4.55E−09	1.0000
24	0.7318	0.0977	0.0981	0.0718	0.0715	4.21E−04	− 4.31E−03	4.02E−09	1.0000
25	0.7702	0.0963	0.0967	0.0745	0.0742	4.48E−04	− 4.66E−03	4.57E−09	1.0000
26	0.8053	0.0937	0.0941	0.0757	0.0755	3.58E−04	− 3.82E−03	2.91E−09	1.0000
27	0.8329	0.0900	0.0902	0.0751	0.0750	1.55E−04	− 1.72E−03	5.48E−10	1.0000
28	0.8550	0.0855	0.0853	0.0729	0.0731	2.46E−04	2.87E−03	1.37E−09	1.0000
29	0.8738	0.0799	0.0795	0.0695	0.0698	3.79E−04	4.74E−03	3.26E−09	1.0000
30	0.8887	0.0743	0.0738	0.0655	0.0660	5.44E−04	7.32E−03	6.72E−09	0.9999
31	0.9016	0.0683	0.0679	0.0612	0.0616	3.73E−04	5.46E−03	3.16E−09	1.0000
32	0.9141	0.0618	0.0614	0.0561	0.0565	4.27E−04	6.91E−03	4.15E−09	0.9999
33	0.9248	0.0555	0.0551	0.0510	0.0513	3.57E−04	6.44E−03	2.90E−09	1.0000
34	0.9344	0.0493	0.0491	0.0459	0.0461	2.25E−04	4.55E−03	1.15E−09	1.0000
35	0.9445	0.0422	0.0423	0.0399	0.0399	5.75E−05	− 1.36E−03	7.51E−11	1.0000
36	0.9533	0.0357	0.0358	0.0342	0.0340	1.48E−04	− 4.15E−03	5.00E−10	1.0000
37	0.9618	0.0291	0.0293	0.0282	0.0280	1.94E−04	− 6.68E−03	8.59E−10	1.0000
38	0.9702	0.0222	0.0226	0.0219	0.0215	3.66E−04	− 1.65E−02	3.05E−09	0.9997
39	0.9778	0.0157	0.0162	0.0159	0.0154	5.27E−04	− 3.36E−02	6.32E−09	0.9988
40	0.9852	0.0092	0.0098	0.0096	0.0091	5.71E−04	− 6.20E−02	7.40E−09	0.9960
41	0.9926	0.0026	0.0029	0.0029	0.0026	3.48E−04	− 1.34E−01	2.76E−09	0.9812
42	0.9999	− 0.0040	− 0.0042	− 0.0042	− 0.0040	1.60E−04	− 4.01E−02	5.85E−10	0.9983
43	1.0046	− 0.0085	− 0.0086	− 0.0087	− 0.0085	1.25E−04	− 1.47E−02	3.53E−10	0.9998
44	1.0089	− 0.0124	− 0.0132	− 0.0133	− 0.0125	8.28E−04	− 6.67E−02	1.56E−08	0.9953
Average values						2.09E−04	− 8.09E−03	1.83E−09	0.9993

**Table 20:** Different evaluation metrics of the Case 2 (TDM).

Data points	${V}_{ex}$(V)	${I}_{ex}$(A)	${I}_{es}$(A)	${P}_{ex}$(W)	${P}_{es}$(W)	$AE (A)$	$RE$	$MBE$	${R}^{2}$
1	− 0.1659	0.1001	0.1002	− 0.0166	− 0.0166	6.93E−05	− 6.93E−04	1.09E−10	1.0000
2	− 0.1281	0.1000	0.1001	− 0.0128	− 0.0128	1.30E−04	− 1.30E−03	3.81E−10	1.0000
3	− 0.0888	0.0999	0.1001	− 0.0089	− 0.0089	1.88E−04	− 1.88E−03	8.05E−10	1.0000
4	− 0.0490	0.0999	0.1000	− 0.0049	− 0.0049	1.46E−04	− 1.46E−03	4.85E−10	1.0000
5	− 0.0102	0.0999	0.1000	− 0.0010	− 0.0010	1.05E−04	− 1.05E−03	2.50E−10	1.0000
6	0.0275	0.0998	0.1000	0.0027	0.0027	1.65E−04	− 1.65E−03	6.18E−10	1.0000
7	0.0695	0.0999	0.0999	0.0069	0.0069	2.00E−05	− 2.00E−04	9.11E−12	1.0000
8	0.1061	0.0998	0.0999	0.0106	0.0106	8.08E−05	− 8.09E−04	1.48E−10	1.0000
9	0.1460	0.0998	0.0998	0.0146	0.0146	3.75E−05	− 3.76E−04	3.20E−11	1.0000
10	0.1828	0.0997	0.0998	0.0182	0.0182	9.73E−05	− 9.76E−04	2.15E−10	1.0000
11	0.2230	0.0997	0.0998	0.0222	0.0222	5.26E−05	− 5.28E−04	6.30E−11	1.0000
12	0.2600	0.0996	0.0997	0.0259	0.0259	1.11E−04	− 1.11E−03	2.79E−10	1.0000
13	0.3001	0.0997	0.0997	0.0299	0.0299	3.59E−05	3.60E−04	2.93E−11	1.0000
14	0.3406	0.0996	0.0996	0.0339	0.0339	1.54E−05	− 1.55E−04	5.40E−12	1.0000
15	0.3789	0.0995	0.0996	0.0377	0.0377	6.71E−05	− 6.75E−04	1.02E−10	1.0000
16	0.4168	0.0994	0.0995	0.0415	0.0414	1.16E−04	− 1.17E−03	3.08E−10	1.0000
17	0.4583	0.0994	0.0995	0.0456	0.0456	5.58E−05	− 5.62E−04	7.08E−11	1.0000
18	0.4949	0.0993	0.0994	0.0492	0.0491	9.61E−05	− 9.67E−04	2.10E−10	1.0000
19	0.5370	0.0993	0.0993	0.0533	0.0533	1.50E−05	− 1.51E−04	5.14E−12	1.0000
20	0.5753	0.0992	0.0992	0.0571	0.0571	2.19E−05	− 2.21E−04	1.09E−11	1.0000
21	0.6123	0.0990	0.0991	0.0607	0.0606	9.89E−05	− 9.99E−04	2.22E−10	1.0000
22	0.6546	0.0988	0.0989	0.0647	0.0647	7.68E−05	− 7.77E−04	1.34E−10	1.0000
23	0.6918	0.0983	0.0985	0.0682	0.0680	2.29E−04	− 2.33E−03	1.20E−09	1.0000
24	0.7318	0.0977	0.0978	0.0716	0.0715	9.45E−05	− 9.68E−04	2.03E−10	1.0000
25	0.7702	0.0963	0.0963	0.0742	0.0742	1.28E−05	− 1.32E−04	3.69E−12	1.0000
26	0.8053	0.0937	0.0936	0.0753	0.0755	1.50E−04	1.60E−03	5.10E−10	1.0000
27	0.8329	0.0900	0.0896	0.0747	0.0750	3.51E−04	3.90E−03	2.81E−09	1.0000
28	0.8550	0.0855	0.0848	0.0725	0.0731	6.85E−04	8.02E−03	1.07E−08	0.9999
29	0.8738	0.0799	0.0792	0.0692	0.0698	7.07E−04	8.85E−03	1.14E−08	0.9999
30	0.8887	0.0743	0.0736	0.0654	0.0660	7.48E−04	1.01E−02	1.27E−08	0.9999
31	0.9016	0.0683	0.0678	0.0612	0.0616	4.57E−04	6.70E−03	4.75E−09	1.0000
32	0.9141	0.0618	0.0614	0.0561	0.0565	3.85E−04	6.22E−03	3.36E−09	1.0000
33	0.9248	0.0555	0.0553	0.0511	0.0513	2.12E−04	3.82E−03	1.02E−09	1.0000
34	0.9344	0.0493	0.0493	0.0461	0.0461	5.28E−07	− 1.07E−05	6.33E−15	1.0000
35	0.9445	0.0422	0.0425	0.0402	0.0399	3.43E−04	− 8.13E−03	2.68E−09	0.9999
36	0.9533	0.0357	0.0362	0.0345	0.0340	4.68E−04	− 1.31E−02	4.99E−09	0.9998
37	0.9618	0.0291	0.0296	0.0285	0.0280	5.23E−04	− 1.80E−02	6.21E−09	0.9997
38	0.9702	0.0222	0.0229	0.0222	0.0215	6.68E−04	− 3.01E−02	1.01E−08	0.9991
39	0.9778	0.0157	0.0165	0.0161	0.0154	7.74E−04	− 4.93E−02	1.36E−08	0.9975
40	0.9852	0.0092	0.0099	0.0098	0.0091	7.35E−04	− 7.99E−02	1.23E−08	0.9933
41	0.9926	0.0026	0.0030	0.0030	0.0026	4.03E−04	− 1.55E−01	3.69E−09	0.9749
42	0.9999	− 0.0040	− 0.0042	− 0.0042	− 0.0040	2.44E−04	− 6.10E−02	1.35E−09	0.9961
43	1.0046	− 0.0085	− 0.0088	− 0.0089	− 0.0085	3.30E−04	− 3.89E−02	2.48E−09	0.9984
44	1.0089	− 0.0124	− 0.0135	− 0.0137	− 0.0125	1.14E−03	− 9.18E−02	2.95E−08	0.9912
Average values						2.60E−04	− 1.17E−02	3.18E−09	0.9988

**Figure 19:**
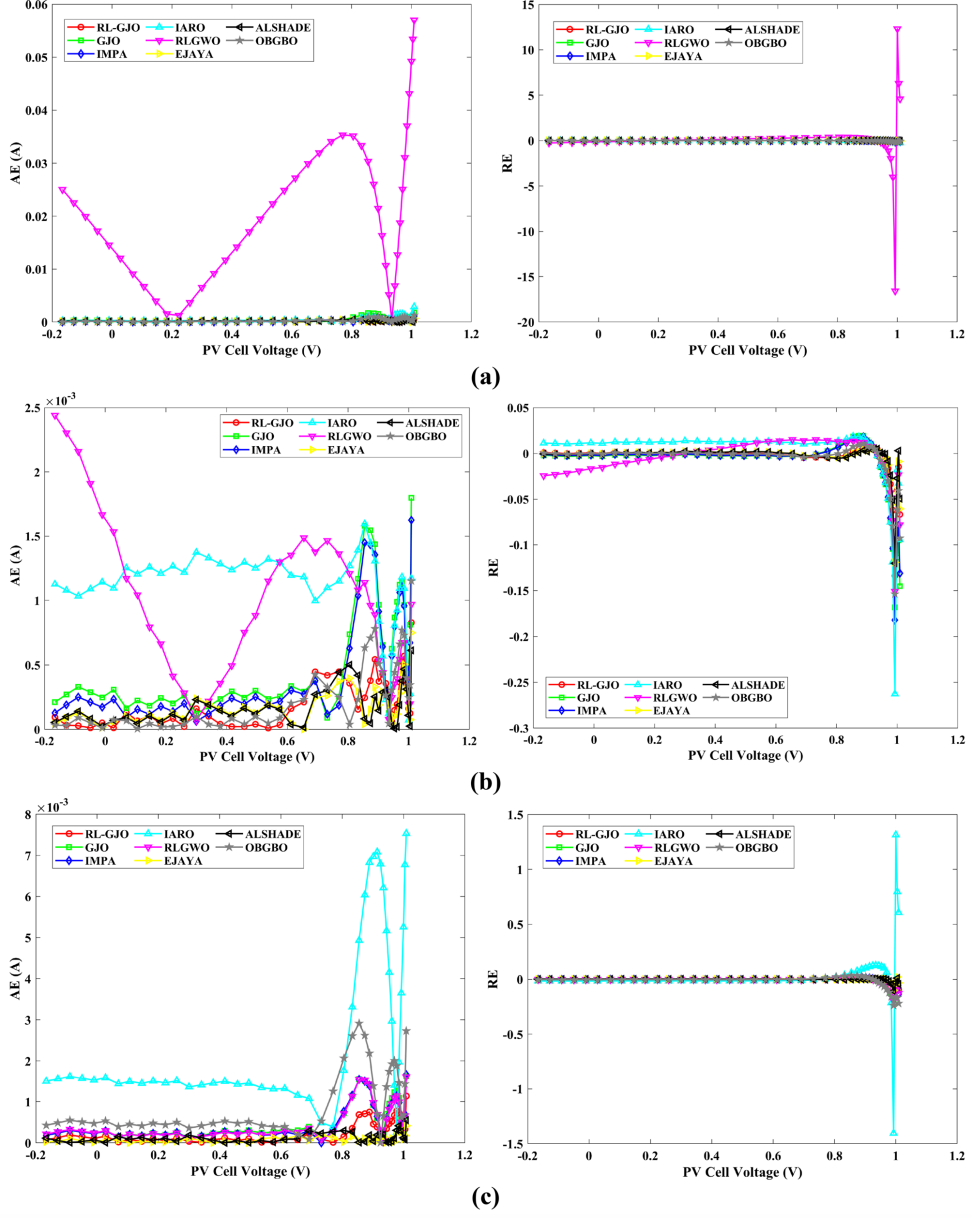
AE and RE Metrics (Case 2); (**a**) SDM, (**b**) DDM, (**c**) TDM.

The study has gathered RE and AE values from multiple algorithms. These values represent how well each algorithm’s predictions match the actual measured cell voltage. To make these results more comprehensible and visually informative, different plots are created. For each algorithm, there is a set of RE and IAE values corresponding to different measured cell voltages. Each algorithm is likely represented by a different line or set of data points on the graph, as illustrated in Fig. 19. Figure 19 can gain insights into how well each algorithm performs at different voltage levels. It allows for a more intuitive understanding of which algorithms perform better or worse under various conditions. This visual representation can help researchers and readers quickly identify trends and patterns in the algorithm’s performance.

#### Results of SDM/DDM/TDM of photowatt-PWP 201 PV module

**Figure 20:**
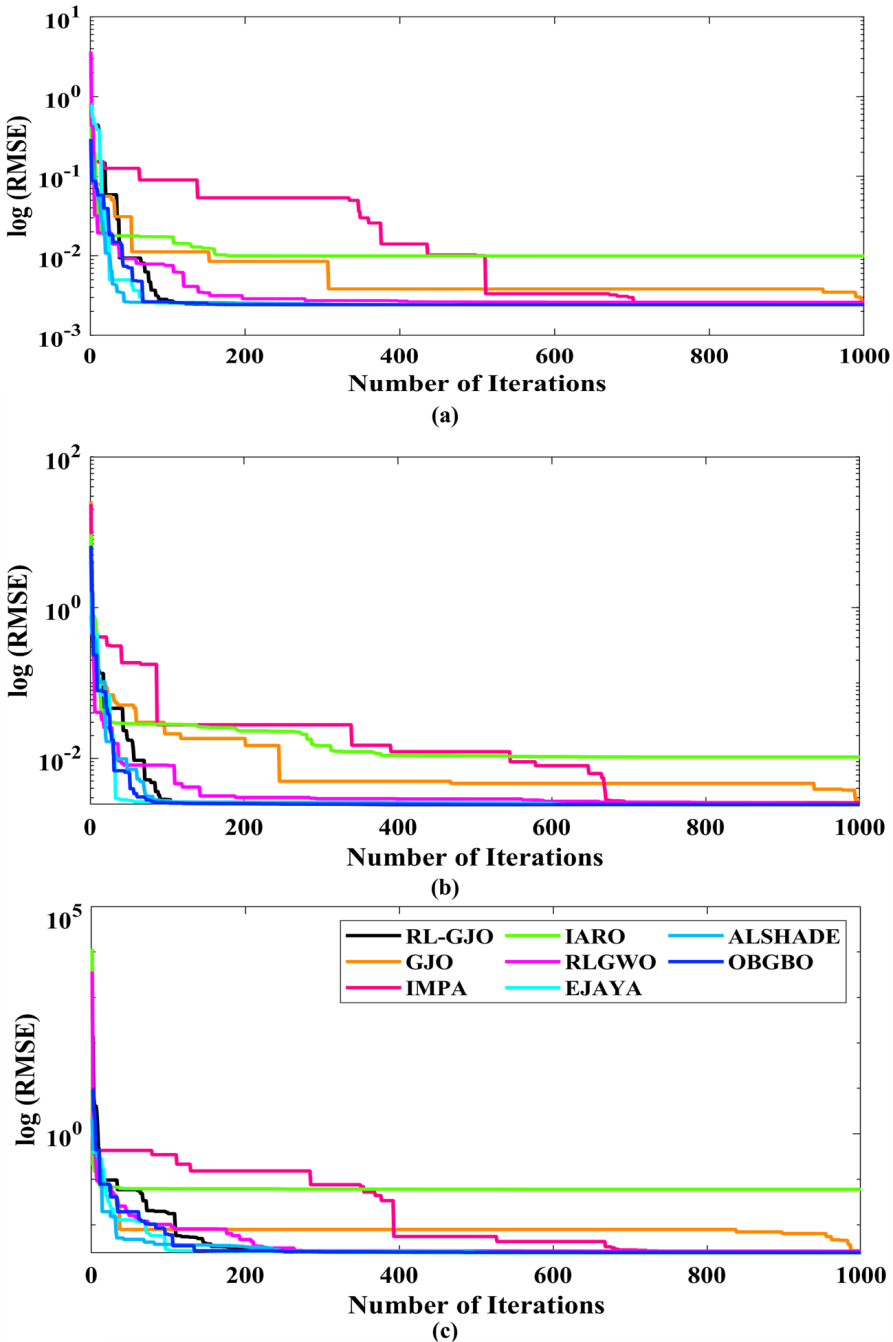
Convergence curves (Case 3); (**a**) SDM, (**b**) DDM, (**c**) TDM.

**Figure 21:**
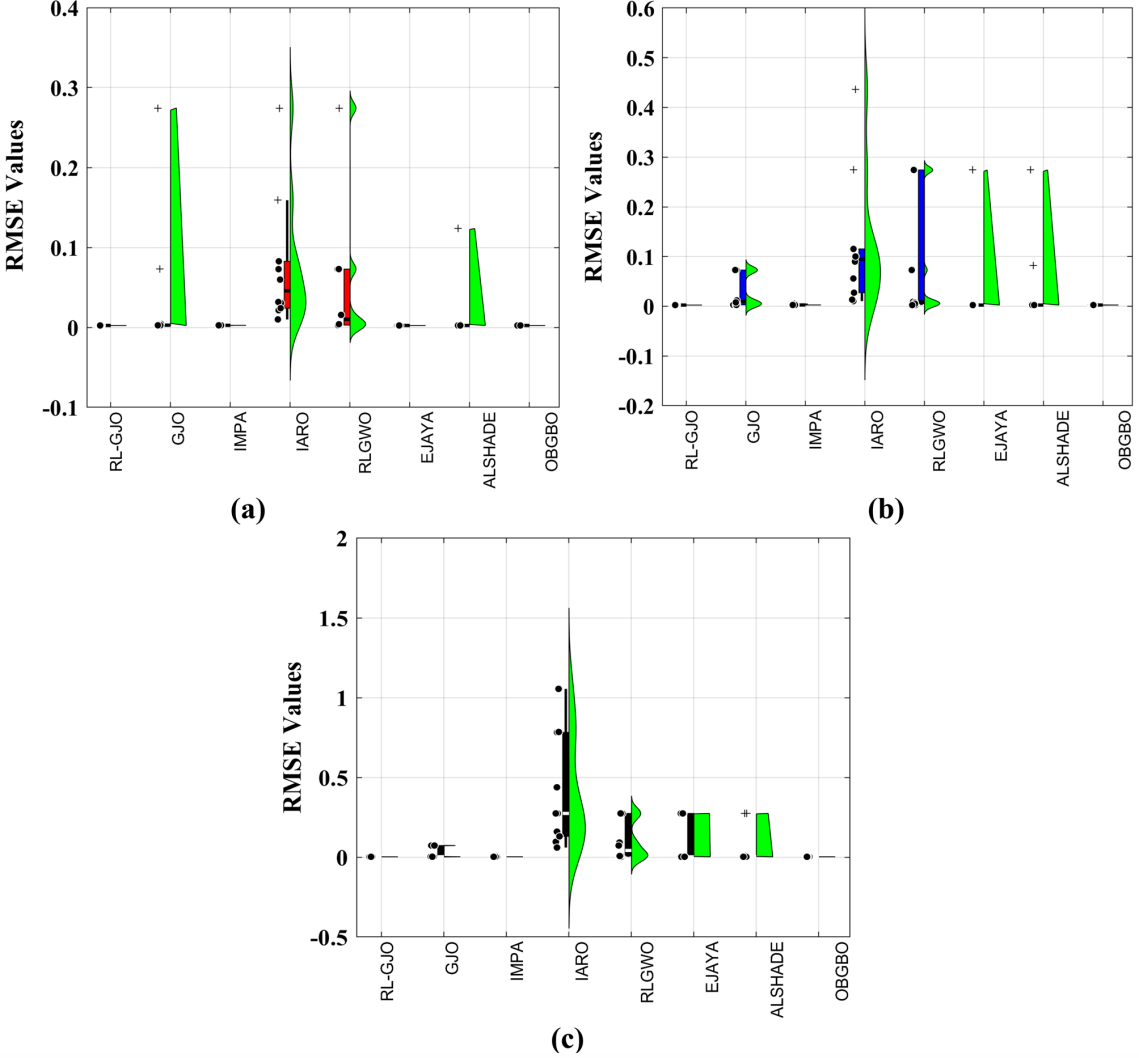
Boxplots (Case 3); (**a**) SDM, (b) DDM, (**c**) TDM.

Similar to the previous cases, this section delves into the outcomes generated by all the algorithms, including the newly proposed RL-GJO. Notably, the reliability and efficacy of RL-GJO exhibit enhancements when compared to the fundamental version of the GJO. Tables 21, 22 and 23 present the parameter results derived from each algorithm following 30 individual runs for the SDM, DDM, and TDM, respectively. Table 21 reveals that the RMSE obtained by the ALSHADE, EJAYA, OBGBO, and RL-GJO is remarkably similar, specifically at 2.43E−03. However, this value surpasses the performance of all the other algorithms in the selection. In Table 22, it is observed that the RMSE value for the DDM, as obtained by the proposed RL-GJO, is remarkably similar to ALSHADE, EJAYA, and OBGBO, significantly outperforms all other algorithms, registering a value of 2.43E−03. Similarly, Table 23 demonstrates that the RMSE value for the TDM achieved by RL-GJO is remarkably similar to ALSHADE, EJAYA, and OBGBO and is notably superior to all other algorithms, standing at 2.43E−03. Even though ALSHADE, EJAYA, and OBGBO results are equal to RL-GJO in the SDM case, RL-GJO exhibits superior reliability and convergence speed across SDM, DDM, and TDM. The convergence curve (Fig. 20) illustrates that RL-GJO’s convergence speed surpasses that of all the selected algorithms, while the box plot (Fig. 21) demonstrates RL-GJO’s higher reliability, denoted by its minimal STD, in comparison to other algorithms. In Tables 21, 22 and 23, symbols like ‘+’ indicate that RL-GJO outperforms other algorithms, ‘−’ indicates a comparatively poorer performance, and ‘=’ signifies an equivalent performance. Furthermore, it is evident from Tables 21, 22 and 23 and Figs. 20 and 21, the performance of the original GJO ranks lower than RL-GJO. The proposed RL-GJO stands first, followed by ALSHADE, EJAYA, OBGBO, IMPA, RLGWO, GJO, and IARO. The paper also explores statistical test analyses to substantiate the superiority of RL-GJO over other algorithms.

**Table 21:** Optimized 5-parameters by selected algorithms for Photowatt-PWP 201 PV module.

Algorithm	${I}_{ph}$(A)	${I}_{sd}$(A)	${R}_{se}$(Ω)	${R}_{sh}$(Ω)	$a$	$RMSE$	*sign*
**RL-GJO**	**1.0305**	**3.48E−06**	**1.2013**	**981.98**	**48.64**	**2.43E−03**	
GJO	1.0281	4.88E−06	1.1649	1651.17	49.97	2.62E−03	+
IMPA	1.0285	3.62E−06	1.2012	1318.25	48.79	2.49E−03	+
IARO	1.0247	4.86E−06	1.0702	1430.93	49.98	9.98E−03	+
RLGWO	1.0285	4.92E−06	1.1652	1637.44	50.00	2.61E−03	+
**EJAYA**	**1.0305**	**3.48E−06**	**1.2013**	**981.98**	**48.64**	**2.43E−03**	=
**ALSHADE**	**1.0305**	**3.48E−06**	**1.2013**	**981.98**	**48.64**	**2.43E−03**	=
**OBGBO**	**1.0305**	**3.48E−06**	**1.2013**	**981.98**	**48.64**	**2.43E−03**	=

**Table 22:** Optimized 7-parameters by selected algorithms for Photowatt-PWP 201 PV module.

Algorithm	${I}_{ph}$(A)	${I}_{sd1}$(A)	${R}_{se}$(Ω)	${R}_{sh}$(Ω)	${a}_{1}$	${I}_{sd2}$(A)	${a}_{2}$	$RSME$	*sign*
**RL-GJO**	**1.0305**	**6.89E−11**	**1.2013**	**981.98**	**30.86**	**3.48E−06**	**48.64**	**2.43E−03**	
GJO	1.0305	0.00E+00	1.1608	1131.44	3.26	4.91E−06	50.00	2.70E−03	+
IMPA	1.0291	0.00E+00	1.1677	1408.04	48.59	4.75E−06	49.86	2.58E−03	+
IARO	1.0268	8.05E−07	1.3800	1828.75	45.80	8.06E−07	45.81	1.04E−02	+
RLGWO	1.0292	3.46E−06	1.1972	1205.35	48.94	2.62E−07	48.30	2.45E−03	+
**EJAYA**	**1.0305**	**3.48E−06**	**1.2013**	**981.98**	**48.64**	**0.00E+00**	**1.00**	**2.43E−03**	=
**ALSHADE**	**1.0305**	**3.48E−06**	**1.2013**	**982.08**	**48.64**	**0.00E+00**	**1.00**	**2.43E−03**	=
**OBGBO**	**1.0303**	**3.54E−06**	**1.1996**	**1017.12**	**48.71**	**0.00E+00**	**1.00**	**2.43E−03**	=

**Table 23:** Optimized 9-parameters by selected algorithms for Photowatt-PWP 201 PV module.

Algorithm	${I}_{ph}$(A)	${I}_{sd1}$(A)	${R}_{se}$(Ω)	${R}_{sh}$(Ω)	${a}_{1}$	${I}_{sd2}$(A)	${a}_{2}$	${I}_{sd3}$(A)	${a}_{3}$	$RSME$	*sign*
**RL-GJO**	**1.0305**	**2.18E−06**	**1.2013**	**981.98**	**49.98**	**3.48E−06**	**48.64**	**0.00E+00**	**1.00**	**2.43E−03**	
GJO	1.0287	0.00E+00	1.1655	1621.37	1.66	0.00E+00	44.91	4.92E−06	50.00	2.63E−03	+
IMPA	1.0278	1.21E−87	1.1717	1794.47	5.00	0.00E+00	2.94	4.71E−06	49.82	2.58E−03	+
IARO	0.9884	2.51E−06	0.6223	1727.56	47.66	0.00E+00	10.33	0.00E+00	19.71	6.04E−02	+
RLGWO	1.0277	4.92E−06	1.1670	1954.97	50.00	0.00E+00	1.48	0.00E+00	1.08	2.63E−03	+
**EJAYA**	**1.0305**	**3.48E−06**	**1.2013**	**981.98**	**48.64**	**0.00E+00**	**50.00**	**0.00E+00**	**50.00**	**2.43E−03**	=
**ALSHADE**	**1.0305**	**5.11E−10**	**1.2013**	**982.19**	**44.86**	**0.00E+00**	**4.78**	**3.48E−06**	**48.64**	**2.43E−03**	=
OBGBO	1.0298	0.00E+00	1.1895	1138.02	34.48	3.89E−06	49.08	1.27E−08	50.00	2.45E−03	=

**Figure 22:**
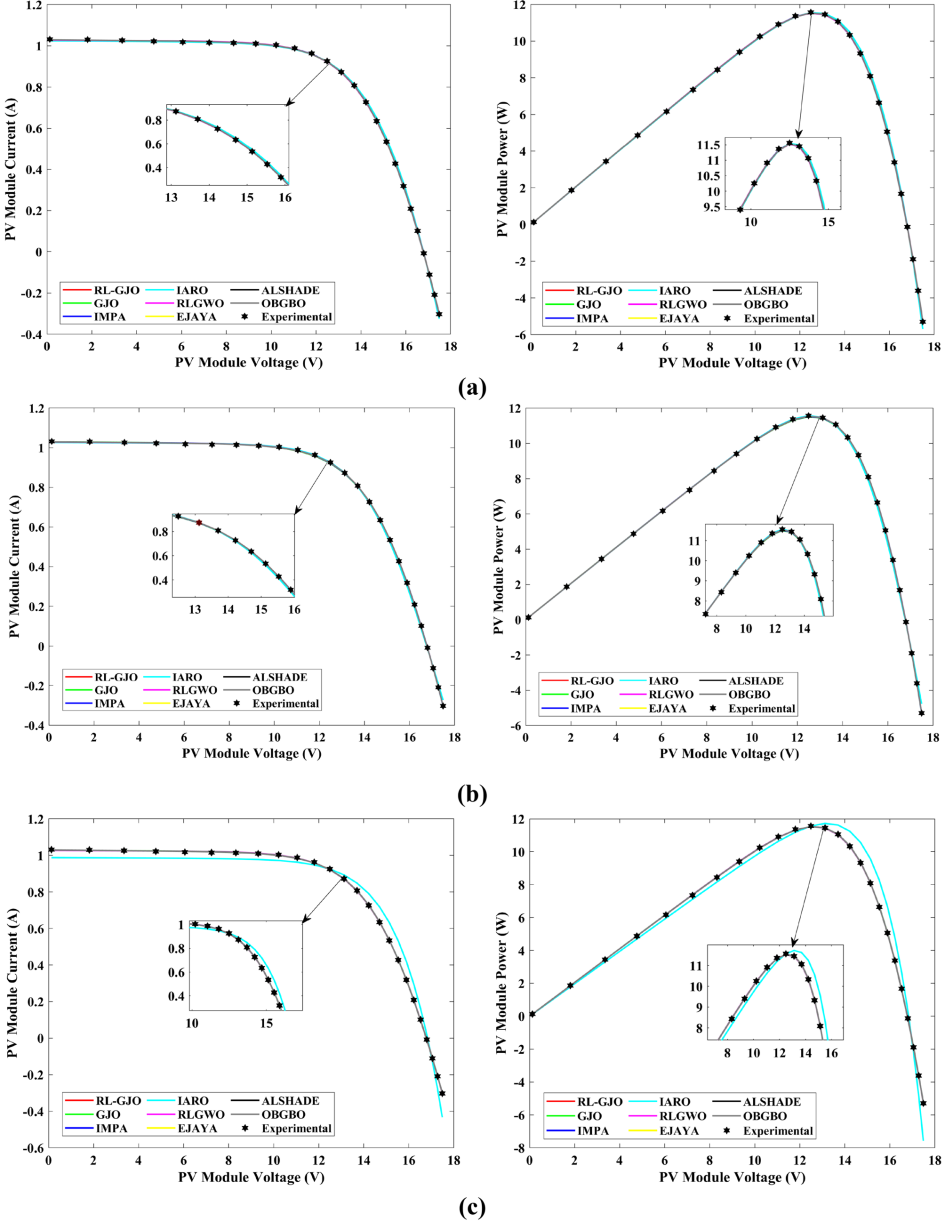
I–V and P–V Characteristics (Case 2); (**a**) SDM, (**b**) DDM, (**c**) TDM.

In addition to the outcomes discussed earlier, an important visual representation of the I–V and P–V characteristics of the Photowatt-PWP 201 PV module, estimated through the proposed RL-GJO for the SDM, DDM, and TDM, is provided in Fig. 22. This figure serves as a compelling illustration of the algorithm’s performance in modelling the behaviour of the solar cell. Upon careful examination of Fig. 22, it becomes evident that the estimated data points closely align with the experimental data points. This alignment showcases a remarkable level of correspondence between the estimated I–V characteristic and the actual behaviour of the Photowatt-PWP 201 PV module. Such a close match between estimated and experimental data is a strong indicator of the efficacy and superiority of the RL-GJO in comparison to other algorithms explored in this study. In essence, Fig. 22 provides tangible evidence of how well RL-GJO captures the intricate details of the I–V and P–V characteristics of the solar cell, thus emphasizing its efficiency and effectiveness in the realm of parameter estimation when compared to competing algorithms.

Tables 24, 25 and 26 provide a comprehensive breakdown of the AE, RE, MBE, and R^2^ values for all the data samples obtained through the utilization of the proposed RL-GJO. These tables offer valuable insights into the accuracy and reliability of the algorithm across different scenarios. Examining Table 24, it is observed that the AE values consistently fall below the threshold of 4.83E−03, while the RE, MBE, and R^2^ stay within the confines of 4.78E−02, 9.34E−07, and 0.9975. These metrics are indicative of the algorithm’s exceptional ability to model and predict the behaviour of the SDM accurately. Moving on to Table 25, it is found that the AE values for this dataset are all below 4.83E−03, and the RE, MBE, and R^2^ are below 4.78E−02, 9.34E−07, and 0.9975. These values underscore the algorithm’s strong performance in capturing the characteristics of the DDM effectively. Similarly, Table 26 reveals that for the TDM dataset, the AE values consistently stay below 4.83E−03, and the RE, MBE, and R^2^ are below 4.78E−02, 9.34E−07, and 0.9975. These statistics underscore the algorithm’s remarkable capacity to extract the actual characteristics of the TDM accurately. When we look at the average values across these datasets, it is found that for the SDM, the average values of AE, RE, MBE, and R^2^ are 1.96E−03, 3.25E−04, 2.35E−07, and 0.9998, respectively. Similarly, for the DDM, the average values of AE, RE, MBE, and R^2^ are 1.96E−03, 3.25E−04, 2.35E−07, and 0.9998, respectively. Finally, for the TDM, the average values of AE, RE, MBE, and R^2^ are 1.96E−03, 3.25E−04, 2.35E−07, and 0.9998, respectively. These average values further reinforce the conclusion that the proposed RL-GJO excels in extracting and representing the true characteristics of SDM, DDM, and TDM datasets accurately. In summary, Tables 24, 25 and 26 and the calculated averages collectively emphasize the impressive performance of the RL-GJO in capturing the behaviours of SDM, DDM, and TDM datasets, thereby underlining its effectiveness and reliability in the realm of parameter estimation.

**Table 24:** Different evaluation metrics of Case 3 (SDM).

Data points	${V}_{ex}$(V)	${I}_{ex}$(A)	${I}_{es}$(A)	${P}_{ex}$(W)	${P}_{es}$(W)	$AE (A)$	$RE$	$MBE$	${R}^{2}$
1	0.1248	1.0315	1.0291	0.1284	0.1287	2.38E−03	2.31E−03	2.27E−07	1.0000
2	1.8093	1.0300	1.0274	1.8588	1.8636	2.62E−03	2.54E−03	2.74E−07	1.0000
3	3.3511	1.0260	1.0257	3.4374	3.4382	2.58E−04	2.52E−04	2.67E−09	1.0000
4	4.7622	1.0220	1.0241	4.8770	4.8670	2.11E−03	− 2.06E−03	1.78E−07	1.0000
5	6.0538	1.0180	1.0223	6.1888	6.1628	4.29E−03	− 4.22E−03	7.37E−07	1.0000
6	7.2364	1.0155	1.0199	7.3806	7.3486	4.43E−03	− 4.36E−03	7.85E−07	1.0000
7	8.3189	1.0140	1.0164	8.4550	8.4354	2.36E−03	− 2.33E−03	2.23E−07	1.0000
8	9.3097	1.0100	1.0105	9.4074	9.4028	4.96E−04	− 4.91E−04	9.85E−09	1.0000
9	10.2163	1.0035	1.0006	10.2227	10.2521	2.87E−03	2.86E−03	3.30E−07	1.0000
10	11.0449	0.9880	0.9845	10.8742	10.9124	3.45E−03	3.49E−03	4.77E−07	1.0000
11	11.8018	0.9630	0.9595	11.3241	11.3651	3.48E−03	3.61E−03	4.84E−07	1.0000
12	12.4929	0.9255	0.9228	11.5289	11.5622	2.66E−03	2.88E−03	2.83E−07	1.0000
13	13.1231	0.8725	0.8726	11.4512	11.4499	9.97E−05	− 1.14E−04	3.97E−10	1.0000
14	13.6983	0.8075	0.8073	11.0583	11.0614	2.26E−04	2.80E−04	2.04E−09	1.0000
15	14.2221	0.7265	0.7283	10.3585	10.3324	1.84E−03	− 2.53E−03	1.35E−07	1.0000
16	14.6995	0.6345	0.6371	9.3656	9.3268	2.64E−03	− 4.16E−03	2.78E−07	1.0000
17	15.1346	0.5345	0.5362	8.1154	8.0894	1.71E−03	− 3.20E−03	1.17E−07	1.0000
18	15.5311	0.4275	0.4295	6.6708	6.6395	2.01E−03	− 4.70E−03	1.62E−07	1.0000
19	15.8929	0.3185	0.3188	5.0663	5.0619	2.74E−04	− 8.62E−04	3.01E−09	1.0000
20	16.2229	0.2085	0.2074	3.3645	3.3825	1.11E−03	5.33E−03	4.93E−08	1.0000
21	16.5241	0.1010	0.0962	1.5891	1.6689	4.83E−03	4.78E−02	9.34E−07	0.9975
22	16.7987	− 0.0080	− 0.0083	− 0.1399	− 0.1344	3.25E−04	− 4.07E−02	4.24E−09	0.9982
23	17.0499	− 0.1110	− 0.1109	− 1.8915	− 1.8925	6.35E−05	5.72E−04	1.61E−10	1.0000
24	17.2793	− 0.2090	− 0.2092	− 3.6156	− 3.6114	2.47E−04	− 1.18E−03	2.45E−09	1.0000
25	17.4885	− 0.3030	− 0.3009	− 5.2617	− 5.2990	2.14E−03	7.05E−03	1.83E−07	0.9999
Average values						1.96E−03	3.25E−04	2.35E−07	0.9998

**Table 25:** Different evaluation metrics of the Case 3 (DDM).

Data points	${V}_{ex}$(V)	${I}_{ex}$(A)	${I}_{es}$(A)	${P}_{ex}$(W)	${P}_{es}$(W)	$AE (A)$	$RE$	$MBE$	${R}^{2}$
1	0.1248	1.0315	1.0291	0.1284	0.1287	2.38E−03	2.31E−03	2.27E−07	1.0000
2	1.8093	1.0300	1.0274	1.8588	1.8636	2.62E−03	2.54E−03	2.74E−07	1.0000
3	3.3511	1.0260	1.0257	3.4374	3.4382	2.58E−04	2.52E−04	2.67E−09	1.0000
4	4.7622	1.0220	1.0241	4.8770	4.8670	2.11E−03	− 2.06E−03	1.78E−07	1.0000
5	6.0538	1.0180	1.0223	6.1888	6.1628	4.29E−03	− 4.22E−03	7.37E−07	1.0000
6	7.2364	1.0155	1.0199	7.3806	7.3486	4.43E−03	− 4.36E−03	7.85E−07	1.0000
7	8.3189	1.0140	1.0164	8.4550	8.4354	2.36E−03	− 2.33E−03	2.23E−07	1.0000
8	9.3097	1.0100	1.0105	9.4074	9.4028	4.96E−04	− 4.91E−04	9.85E−09	1.0000
9	10.2163	1.0035	1.0006	10.2227	10.2521	2.87E−03	2.86E−03	3.30E−07	1.0000
10	11.0449	0.9880	0.9845	10.8742	10.9124	3.45E−03	3.49E−03	4.77E−07	1.0000
11	11.8018	0.9630	0.9595	11.3241	11.3651	3.48E−03	3.61E−03	4.84E−07	1.0000
12	12.4929	0.9255	0.9228	11.5289	11.5622	2.66E−03	2.88E−03	2.83E−07	1.0000
13	13.1231	0.8725	0.8726	11.4512	11.4499	9.97E−05	− 1.14E−04	3.97E−10	1.0000
14	13.6983	0.8075	0.8073	11.0583	11.0614	2.26E−04	2.80E−04	2.04E−09	1.0000
15	14.2221	0.7265	0.7283	10.3585	10.3324	1.84E−03	− 2.53E−03	1.35E−07	1.0000
16	14.6995	0.6345	0.6371	9.3656	9.3268	2.64E−03	− 4.16E−03	2.78E−07	1.0000
17	15.1346	0.5345	0.5362	8.1154	8.0894	1.71E−03	− 3.20E−03	1.17E−07	1.0000
18	15.5311	0.4275	0.4295	6.6708	6.6395	2.01E−03	− 4.70E−03	1.62E−07	1.0000
19	15.8929	0.3185	0.3188	5.0663	5.0619	2.74E−04	− 8.62E−04	3.01E−09	1.0000
20	16.2229	0.2085	0.2074	3.3645	3.3825	1.11E−03	5.33E−03	4.93E−08	1.0000
21	16.5241	0.1010	0.0962	1.5891	1.6689	4.83E−03	4.78E−02	9.34E−07	0.9975
22	16.7987	− 0.0080	− 0.0083	− 0.1399	− 0.1344	3.25E−04	− 4.07E−02	4.24E−09	0.9982
23	17.0499	− 0.1110	− 0.1109	− 1.8915	− 1.8925	6.35E−05	5.72E−04	1.61E−10	1.0000
24	17.2793	− 0.2090	− 0.2092	− 3.6156	− 3.6114	2.47E−04	− 1.18E−03	2.45E−09	1.0000
25	17.4885	− 0.3030	− 0.3009	− 5.2617	− 5.2990	2.14E−03	7.05E−03	1.83E−07	0.9999
Average values						1.96E−03	3.25E−04	2.35E−07	0.9998

**Table 26:** Different evaluation metrics of the Case 3 (TDM).

Data points	${V}_{ex}$(V)	${I}_{ex}$(A)	${I}_{es}$(A)	${P}_{ex}$(W)	${P}_{es}$(W)	$AE (A)$	$RE$	$MBE$	${R}^{2}$
1	0.1248	1.0315	1.0291	0.1284	0.1287	2.38E−03	2.31E−03	2.27E−07	1.0000
2	1.8093	1.0300	1.0274	1.8588	1.8636	2.62E−03	2.54E−03	2.74E−07	1.0000
3	3.3511	1.0260	1.0257	3.4374	3.4382	2.58E−04	2.52E−04	2.67E−09	1.0000
4	4.7622	1.0220	1.0241	4.8770	4.8670	2.11E−03	− 2.06E−03	1.78E−07	1.0000
5	6.0538	1.0180	1.0223	6.1888	6.1628	4.29E−03	− 4.22E−03	7.37E−07	1.0000
6	7.2364	1.0155	1.0199	7.3806	7.3486	4.43E−03	− 4.36E−03	7.85E−07	1.0000
7	8.3189	1.0140	1.0164	8.4550	8.4354	2.36E−03	− 2.33E−03	2.23E−07	1.0000
8	9.3097	1.0100	1.0105	9.4074	9.4028	4.96E−04	− 4.91E−04	9.85E−09	1.0000
9	10.2163	1.0035	1.0006	10.2227	10.2521	2.87E−03	2.86E−03	3.30E−07	1.0000
10	11.0449	0.9880	0.9845	10.8742	10.9124	3.45E−03	3.49E−03	4.77E−07	1.0000
11	11.8018	0.9630	0.9595	11.3241	11.3651	3.48E−03	3.61E−03	4.84E−07	1.0000
12	12.4929	0.9255	0.9228	11.5289	11.5622	2.66E−03	2.88E−03	2.83E−07	1.0000
13	13.1231	0.8725	0.8726	11.4512	11.4499	9.97E−05	− 1.14E−04	3.97E−10	1.0000
14	13.6983	0.8075	0.8073	11.0583	11.0614	2.26E−04	2.80E−04	2.04E−09	1.0000
15	14.2221	0.7265	0.7283	10.3585	10.3324	1.84E−03	− 2.53E−03	1.35E−07	1.0000
16	14.6995	0.6345	0.6371	9.3656	9.3268	2.64E−03	− 4.16E−03	2.78E−07	1.0000
17	15.1346	0.5345	0.5362	8.1154	8.0894	1.71E−03	− 3.20E−03	1.17E−07	1.0000
18	15.5311	0.4275	0.4295	6.6708	6.6395	2.01E−03	− 4.70E−03	1.62E−07	1.0000
19	15.8929	0.3185	0.3188	5.0663	5.0619	2.74E−04	− 8.62E−04	3.01E−09	1.0000
20	16.2229	0.2085	0.2074	3.3645	3.3825	1.11E−03	5.33E−03	4.93E−08	1.0000
21	16.5241	0.1010	0.0962	1.5891	1.6689	4.83E−03	4.78E−02	9.34E−07	0.9975
22	16.7987	− 0.0080	− 0.0083	− 0.1399	− 0.1344	3.25E−04	− 4.07E−02	4.24E−09	0.9982
23	17.0499	− 0.1110	− 0.1109	− 1.8915	− 1.8925	6.35E−05	5.72E−04	1.61E−10	1.0000
24	17.2793	− 0.2090	− 0.2092	− 3.6156	− 3.6114	2.47E−04	− 1.18E−03	2.45E−09	1.0000
25	17.4885	− 0.3030	− 0.3009	− 5.2617	− 5.2990	2.14E−03	7.05E−03	1.83E−07	0.9999
Average values						1.96E−03	3.25E−04	2.35E−07	0.9998

**Figure 23:**
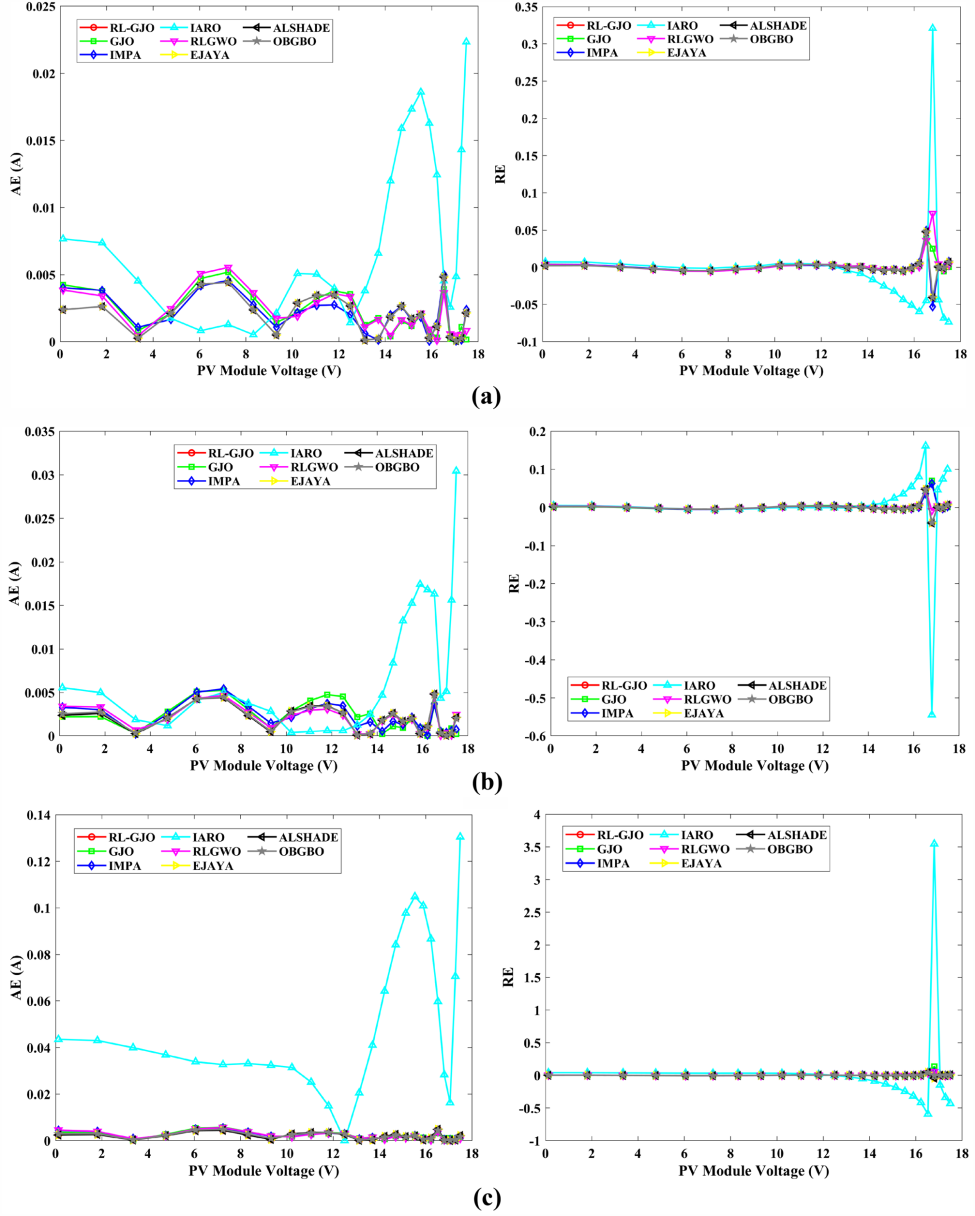
AE and RE Metrics (Case 3); (**a**) SDM, (**b**) DDM, (**c**) TDM.

The study has gathered RE and AE values from multiple algorithms. These values represent how well each algorithm’s predictions match the actual measured cell voltage. To make these results more comprehensible and visually informative, different plots are created. For each algorithm, there is a set of RE and IAE values corresponding to different measured cell voltages. Each algorithm is likely represented by a different line or set of data points on the graph, as illustrated in Fig. 23. Figure 23 can gain insights into how well each algorithm performs at different voltage levels. It allows for a more intuitive understanding of which algorithms perform better or worse under various conditions. This visual representation can help researchers and readers quickly identify trends and patterns in the algorithm’s performance.

### Case 4—SDM of the commercial SM55 monocrystalline PV module

The SM55 is a specific type of PV module made up of monocrystalline solar cells. Monocrystalline cells are made from a single, pure silicon crystal, and they tend to be more efficient than their polycrystalline counterparts. The module comprises 36 cells connected in series. This series connection implies that the current flowing through each cell is the same, but the voltage is the sum of the voltages across each cell. The proposed RL-GJO algorithm is applied to estimate the parameters of the SM55 module based on the SDM model. To verify how well the algorithm performs, experimental values from the commercial module under different temperature and irradiance conditions are collected. Comparing the algorithm’s estimations with these real-world measurements gives an idea of its accuracy. Equation (37) presents the short-circuit current ${I}_{sc}$ under various operating conditions.

46 $${I}_{sc}\left(T,G\right)=\frac{G}{{G}_{STC}}\times {I}_{sc-STC}+\alpha \left(T-{T}_{STC}\right)$$

where $\alpha$ signifies the temperature coefficient of the short-circuit current, ${I}_{sc-STC}$ signifies the short-circuit current under Standard Testing Condition (STC), $T$ and ${T}_{STC}$ denotes the temperature of the module under actual and STC, and $G$ and ${G}_{STC}$ denotes the irradiation of the module under actual and STC. The term $\frac{G}{{G}_{STC}}\times {I}_{sc-STC}$ calculates how the short-circuit current changes with irradiance. If G is the same as ${G}_{STC}$, the short-circuit current remains ${I}_{sc-STC}$. The term $\alpha \left(T-{T}_{STC}\right)$ accounts for the change in short-circuit current due to temperature deviations from STC.

The proposed algorithm and other peers are tested on their ability to estimate the parameters of the SM55, a commercial PV module. This validation process is essential for evaluating the algorithm’s precision and reliability, especially when applied to real-world situations. The effectiveness of any model or algorithm is determined by how accurately it can predict the behaviour of the PV module under different conditions. The proposed algorithm is the focal algorithm being discussed, validated for its capability to estimate parameters essential for the performance characterization of the SM55 PV module. For this study, the SDM is used to provide a theoretical framework. The SDM is a widely accepted electrical model to describe the I–V and P–V characteristics of the PV module. Within this framework, the RL-GJO algorithm and a few other selected algorithms are tasked with estimating the relevant parameters. The experiment involves recording the I–V and P–V characteristics of the SM55 PV module under varying irradiance conditions. These irradiance conditions represent different levels of sunlight intensity: 200 W/m^2^, 400 W/m^2^, 600 W/m^2^, 800 W/m^2^, and 1000 W/m^2^. While altering the irradiance, the temperature is maintained at a consistent 25 °C. This consistent temperature ensures that any variations observed in the module’s performance are solely due to changes in sunlight intensity. These recordings serve as the experimental data points. After collecting this data, the proposed algorithm, along with other selected algorithms, is applied to estimate the PV module’s parameters based on the SDM. All the estimated parameters derived for the SM55 PV module within the SDM framework are systematically listed in Table 27.

**Table 27:** Optimized 5-parameters of SM55 module under different irradiation and 25 ℃ temperature by all algorithms.

*G*	Algorithm	${I}_{ph}$(A)	${I}_{sd}$(A)	${R}_{se}$(Ω)	${R}_{sh}$(Ω)	$a$	$RMSE$
1000 W/m^2^	**RL-GJO**	**3.4502**	**1.73E−07**	**0.3288**	**480.13**	**1.3965**	**1.1549E−03**
	GJO	3.4653	5.49E−05	0.0000	1005.05	2.1198	3.8947E−02
	IMPA	3.4463	7.58E−06	0.1506	4999.71	1.7991	2.1554E−02
	IARO	3.4901	1.75E−05	0.4537	4125.15	1.9505	1.6461E−01
	RLGWO	3.4531	3.17E−05	0.0000	1000.58	2.0180	3.7297E−02
	EJAYA	3.4479	2.43E−07	0.3161	564.16	1.4251	1.4770E−03
	ALSHADE	3.4447	3.41E−07	0.3098	799.35	1.4551	4.2270E−03
	OBGBO	3.4408	1.96E−06	0.2296	4617.02	1.6308	1.2573E−02
800 W/m^2^	**RL-GJO**	**2.7604**	**1.44E−07**	**0.3377**	**459.52**	**1.3810**	**6.6861E−04**
	GJO	2.7516	7.65E−06	0.0000	3440.56	1.7947	1.3388E−02
	IMPA	2.7524	1.10E−05	0.0000	4998.72	1.8501	1.3320E−02
	IARO	2.7865	7.59E−05	0.2294	3372.05	2.2394	7.6404E−02
	RLGWO	2.7583	8.70E−06	0.0157	1016.29	1.8166	1.3424E−02
	EJAYA	2.7567	2.16E−07	0.3144	563.13	1.4145	1.5508E−03
	ALSHADE	2.7455	6.40E−07	0.2613	4987.69	1.5135	6.5282E−03
	OBGBO	2.7508	2.95E−06	0.1265	2087.90	1.6770	8.8223E−03
600 W/m^2^	RL-GJO	2.0704	2.41E−07	0.2997	481.36	1.4253	1.3667E−03
	GJO	2.0700	1.21E−05	0.0000	1488.09	1.8864	1.5924E−02
	IMPA	2.0620	2.65E−06	0.1046	1598.95	1.6737	7.5186E−03
	IARO	2.0886	8.31E−05	0.1056	2564.70	2.2681	5.7956E−02
	RLGWO	2.0843	8.40E−06	0.0111	361.31	1.8325	1.9227E−02
	**EJAYA**	**2.0709**	**1.55E−07**	**0.3305**	**450.05**	**1.3875**	**8.2395E−04**
	ALSHADE	2.0707	2.41E−07	0.2929	458.27	1.4252	1.6903E−03
	OBGBO	2.0647	1.31E−06	0.1696	908.50	1.5923	5.5961E−03
400 W/m^2^	**RL-GJO**	**1.3826**	**1.01E−07**	**0.3963**	**431.34**	**1.3526**	**7.1421E−04**
	GJO	1.3808	8.95E−06	0.0293	1175.28	1.8650	1.4281E−02
	IMPA	1.3690	2.56E−06	0.0232	4838.13	1.6767	6.6649E−03
	IARO	1.3697	1.66E−06	0.0210	2024.32	1.6217	5.8309E−03
	RLGWO	1.3727	6.07E−06	0.0000	4553.91	1.7993	9.7091E−03
	EJAYA	1.3817	1.94E−07	0.3121	463.04	1.4071	1.0368E−03
	ALSHADE	1.3824	1.22E−07	0.3730	439.06	1.3677	7.4903E−04
	OBGBO	1.3792	9.69E−07	0.0550	557.07	1.5616	2.9361E−03
200 W/m^2^	RL-GJO	0.6920	1.37E−07	0.2972	438.04	1.3748	5.2093E−04
	GJO	0.6801	4.57E−06	0.0101	4881.48	1.7853	7.3992E−03
	IMPA	0.6788	1.97E−06	0.0094	3876.80	1.6611	6.5416E−03
	IARO	0.6913	7.78E−05	0.8415	1810.23	2.4382	2.4152E−02
	RLGWO	0.6863	2.89E−06	0.0000	914.32	1.7195	5.5646E−03
	**EJAYA**	**0.6920**	**1.31E−07**	**0.3124**	**438.04**	**1.3709**	**5.2054E−04**
	ALSHADE	0.6920	1.31E−07	0.3134	438.01	1.3706	5.2055E−04
	OBGBO	0.6912	3.08E−07	0.0352	458.46	1.4484	7.3624E−04

**Figure 24:**
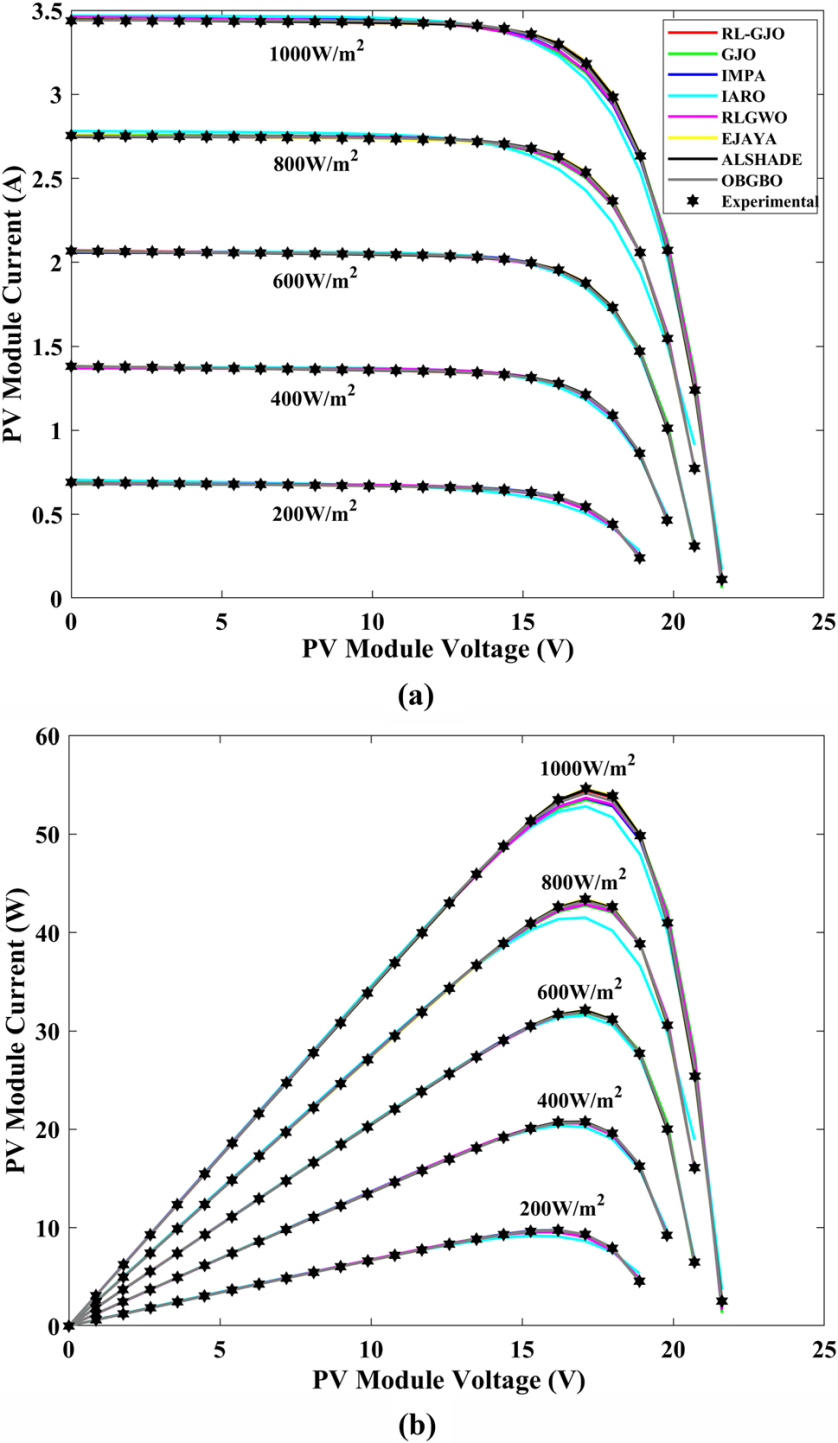
Characteristics of the SM55 module obtained by all algorithms under different irradiation conditions; (**a**) P–V, (**b**) I–V.

Moreover, to provide a visual representation, the I–V and P–V characteristics derived from these estimations for the selected PV module using SDM are graphically illustrated in Fig. 24. These figures are crucial because they visually compare the estimated data (from all the algorithms) with the experimental data. If the estimated and experimental data points align closely, it signifies that the algorithm’s predictions are precise. The comparison, as indicated by Fig. 24, demonstrates a high degree of accuracy in the curve fitting between estimated and experimental data, especially across various irradiance conditions. From the discussions and observations, one can deduce a significant conclusion that the proposed RL-GJO algorithm is proficient at accurately solving multimodal problems, especially in the context of PV module parameter estimation under variable irradiance conditions. This validation not only speaks to the algorithm’s capability but also underscores its potential applications in real-world solar energy scenarios.

In addition to the above experiment, the focus is on understanding how the SM55 PV module behaves under different temperature scenarios. The chosen temperatures for study are 25 °C, 40 °C, and 60 °C. These temperatures span from relatively cool to considerably hot, offering a broad spectrum of performance data. While the temperature is varied in the experiment, the sunlight intensity or irradiance is kept constant at 1000 W/m^2^. This ensures that any changes or variations in the I–V and P–V characteristics are due to temperature changes alone, eliminating the variable of sunlight intensity from the equation. Table 28 is pivotal as it lists out the parameters of the PV modules obtained by all algorithms, specifically the SM55 when modelled using the SDM. These parameters essentially define the model’s understanding of the PV module’s behaviour.

**Table 28:** Optimized 5-parameters of SM55 module under different temperatures and 1000 W/m^2^ irradiation by all algorithms.

$T$	Algorithm	${I}_{ph}$(A)	${I}_{sd}$(A)	${R}_{se}$(Ω)	${R}_{sh}$(Ω)	$a$	$RMSE$
25 °C	**RL-GJO**	**3.4501**	**1.71E−07**	**0.3291**	**483.90**	**1.3958**	**1.1462E−03**
	GJO	3.4572	3.90E−05	0.0422	2860.09	2.0568	3.4992E−02
	IMPA	3.4498	1.63E−05	0.0979	4999.95	1.9105	2.7214E−02
	IARO	3.4807	5.52E−05	0.1422	1712.95	2.1299	6.9311E−02
	RLGWO	3.4646	1.39E−05	0.1099	553.98	1.8872	3.0050E−02
	EJAYA	3.4439	3.77E−07	0.3014	825.26	1.4641	3.7282E−03
	ALSHADE	3.4494	2.24E−07	0.3190	503.75	1.4182	1.9395E−03
	OBGBO	3.4401	1.90E−06	0.2270	4998.49	1.6269	1.2538E−02
40 °C	**RL-GJO**	**3.4691**	**1.15E−06**	**0.3131**	**533.07**	**1.4178**	**3.7888E−03**
	GJO	3.4709	9.72E−05	0.0000	4629.32	2.0120	3.7987E−02
	IMPA	3.4673	1.27E−05	0.1933	2536.56	1.6884	1.6698E−02
	IARO	3.5456	1.65E−05	0.3952	193.37	1.7402	1.2490E−01
	RLGWO	3.4680	1.80E−05	0.1687	3254.73	1.7370	1.9495E−02
	EJAYA	3.4679	1.26E−06	0.3095	590.26	1.4272	3.8319E−03
	ALSHADE	3.4675	1.30E−06	0.3079	602.18	1.4296	3.8809E−03
	OBGBO	3.4601	4.48E−06	0.2514	4925.99	1.5593	9.1290E−03
60 °C	RL-GJO	3.4937	7.96E−06	0.3122	538.16	1.4203	3.8864E−03
	GJO	3.5273	9.53E−05	0.0743	159.10	1.7549	4.1903E−02
	IMPA	3.4919	4.89E−05	0.2072	4995.40	1.6492	1.4760E−02
	IARO	3.5258	4.67E−05	0.4914	3758.59	1.6574	1.5981E−01
	RLGWO	3.4876	2.02E−05	0.2682	3259.67	1.5290	7.9181E−03
	**EJAYA**	**3.4946**	**6.91E−06**	**0.3187**	**484.88**	**1.4051**	**3.7804E−03**
	ALSHADE	3.4829	1.16E−05	0.2986	4999.99	1.4624	5.9185E−03
	OBGBO	3.4875	1.52E−05	0.2806	1642.36	1.4939	6.2548E−03

**Figure 25:**
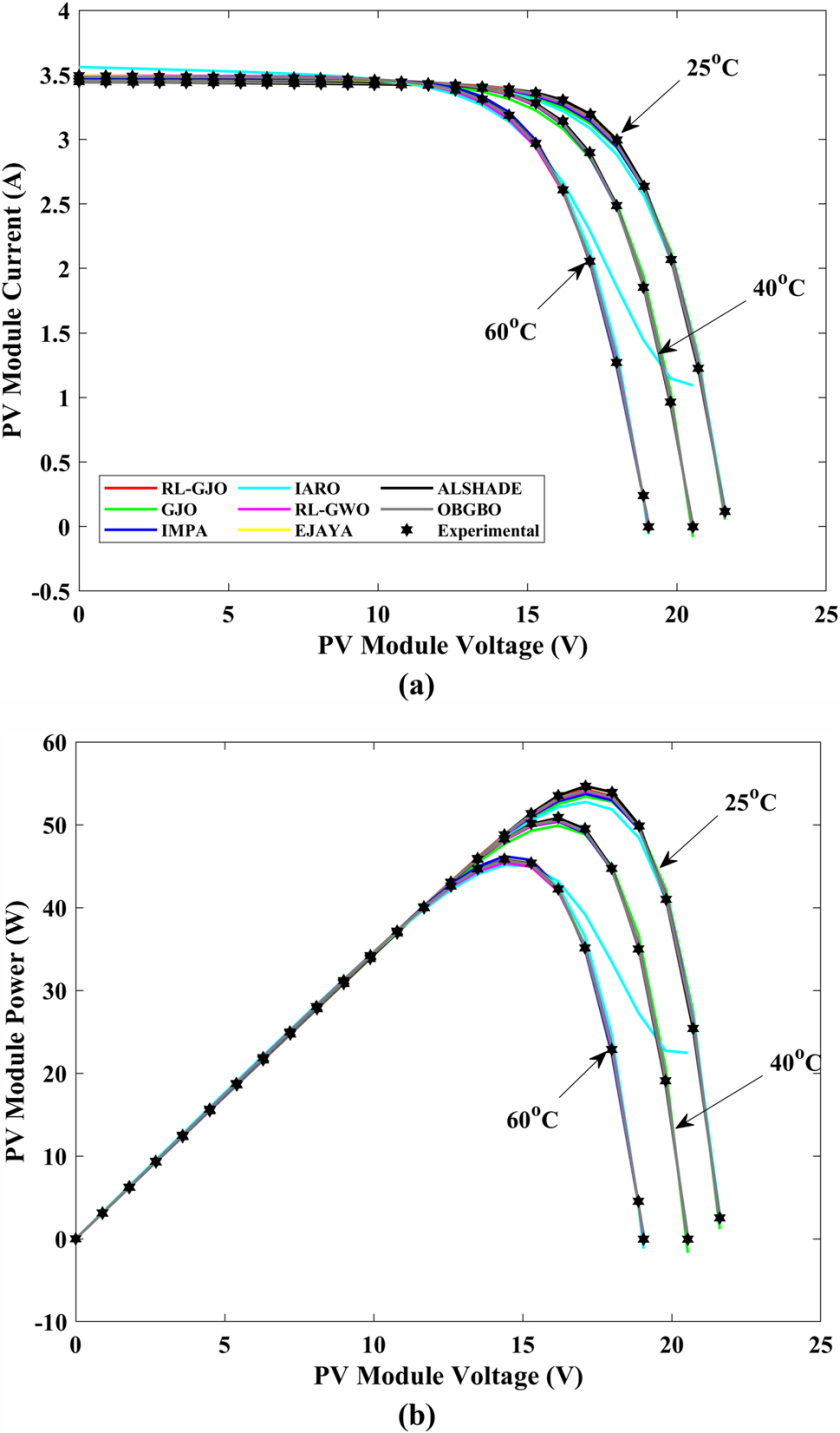
Characteristics of the SM55 module obtained by all algorithms under different temperature conditions; (**a**) P–V, (**b**) I–V.

Figure 25 serves as a visual representation of the I–V and P–V characteristics of the SM55 PV module, as deduced using the SDM. The smartness of this figure lies in its comparative nature: it contrasts the “estimated” I–V and P–V characteristics against the “experimental” data points. If these two sets of data align closely on the graph, it signifies that the model’s predictions are remarkably accurate. From the given context, Fig. 25 evidently demonstrates an impressive degree of accuracy in the curve fitting between the estimated and the experimental data, especially across the varying temperature conditions. The experiment, as detailed, showcases the proficiency of the modelling technique in predicting the behaviour of the SM55 PV module across different temperatures with constant irradiance. The close alignment of the estimated (by RLGJO) and experimental data, as visualized in Fig. 25, underscores the precision and reliability of the model, making it a valuable tool for predicting PV module performance under varying real-world conditions.

Throughout the discussions, a significant emphasis has been placed on the comparative analysis of experimental and estimated data points for selected PV module. The results of this comparison have been overwhelmingly positive. The experimental and estimated data have shown a remarkable alignment across different operating conditions, implying that the algorithms or models in use can predict the behaviour of the PV modules with a high degree of accuracy. Another noteworthy metric brought into focus is the RMSE. RMSE serves as a measure of the differences between values predicted by a model or estimator and the values observed. Essentially, a lower RMSE signifies that the predicted values are closer to the observed values, indicating a higher accuracy of the prediction. For the proposed RL-GJO, the RMSE values have been particularly impressive. Specifically, on the collected data samples, the RMSE values have been found to be relatively low, underscoring the precision and reliability of the RL-GJO algorithm’s predictions.

The I–V and P–V characteristics further substantiate the efficacy of the RL-GJO algorithm. These characteristics provide a visual representation of the relationship between the current flowing through a PV module and the voltage across it. By analyzing how closely the I–V and P–V curves match the real-world observations, one can gauge the algorithm’s accuracy. In this case, the I–V and P–V characteristics generated using the RL-GJO algorithm have exhibited an exceptional alignment with real-world data. This showcases not just the accuracy but also the efficiency of the RL-GJO algorithm in modelling and predicting the performance of PV modules.

In light of the evidence presented, a resounding conclusion can be drawn regarding the RL-GJO algorithm. This proposed algorithm stands out as a robust, accurate, and efficient tool for deducing unknown parameters of various PV models. Its ability to closely match experimental data, its commendable RMSE values, and the precise I–V characteristics it produces all collectively attest to its potential as a premier tool in the realm of PV module analysis and prediction.

### Performance comparison

The efficacy of the proposed RL-GJO has been thoroughly examined through a variety of methods: (i) The RL-GJO was put head-to-head against several other leading algorithms to gauge its performance in comparison, (ii) Obtained data was meticulously analyzed to draw meaningful conclusions about the algorithm’s performance metrics, and (iii) The Friedman Rank Test (FRT) was specifically employed. This test is a robust tool in statistical analysis that helps determine if there are any significant differences between two sets of data. For a well-rounded analysis, the proposed RL-GJO algorithm was compared with several state-of-the-art algorithms, as stated earlier. To comprehensively evaluate the algorithms, a variety of performance metrics were used: (i) Min, Max, Mean, and Median provide fundamental statistics about the data set for each algorithm; (ii) STD measures the amount of variation or dispersion in a set of values. A lower STD indicates that the values tend to be close to the mean, while a high STD indicates wide discrepancies in values, (iii) Run Time (RT) measures how long each algorithm takes to execute. These metrics for the discussed algorithms are tabulated in Table 29 across four distinct cases (Cases 1–4).

**Table 29:** Performance metrics for all case studies.

Algorithm	Min	Max	Mean	Median	STD	RT	FRT
Case 1—SDM							
RL-GJO	9.8602E−04	9.8602E−04	9.8602E−04	9.8602E−04	7.6945E−11	8.877	1.3
GJO	3.3403E−03	4.6040E−02	2.8168E−02	3.8563E−02	1.8469E−02	8.397	7.8
IMPA	1.0153E−03	1.4512E−02	3.1824E−03	2.0368E−03	4.0804E−03	17.364	5.4
IARO	1.5139E−03	5.6968E−02	8.8384E−03	2.4937E−03	1.7053E−02	20.861	6.5
RLGWO	1.3306E−03	3.8169E−02	9.9420E−03	3.0728E−03	1.4925E−02	9.572	6.3
EJAYA	9.8602E−04	9.8605E−04	9.8603E−04	9.8602E−04	8.6292E−09	15.453	2.8
ALSHADE	9.8602E−04	9.9478E−04	9.8712E−04	9.8604E−04	2.7465E−06	79.152	2.8
OBGBO	9.8602E−04	1.1876E−03	1.0223E−03	9.9297E−04	6.2523E−05	9.194	3.6
Case 1—DDM							
RL-GJO	9.8255E−04	1.2026E−03	1.0204E−03	9.8602E−04	6.8918E−05	8.369	2.1
GJO	2.0603E−03	4.5515E−02	2.2894E−02	2.2377E−02	1.9438E−02	8.181	7
IMPA	1.4538E−03	3.0821E−03	2.4905E−03	2.5199E−03	5.4655E−04	17.072	5.6
IARO	1.9164E−03	6.8173E−02	2.3810E−02	1.5679E−02	2.4052E−02	20.473	6.9
RLGWO	1.4993E−03	2.2286E−01	3.0342E−02	4.2702E−03	6.8606E−02	8.603	6.5
EJAYA	9.8350E−04	1.3603E−03	1.0232E−03	9.8602E−04	1.1846E−04	16.216	2.5
ALSHADE	9.8292E−04	1.1807E−03	1.0265E−03	9.8586E−04	8.1259E−05	79.425	2.1
OBGBO	9.8610E−04	1.3132E−03	1.1257E−03	1.0813E−03	1.2835E−04	8.652	3.3
Case 1—TDM							
RL-GJO	9.8085E−04	1.2919E−03	1.0206E−03	9.8533E−04	9.6502E−05	9.642	1.8
GJO	4.0595E−03	4.6002E−02	3.1393E−02	4.0166E−02	1.8213E−02	9.181	7.3
IMPA	9.8792E−04	2.6930E−03	1.9191E−03	2.0706E−03	6.6410E−04	17.691	4.6
IARO	2.1938E−03	2.0454E−02	6.9453E−03	5.4406E−03	5.6446E−03	22.117	6.6
RLGWO	1.3802E−03	4.5817E−02	2.4979E−02	3.2907E−02	1.6678E−02	10.666	7
EJAYA	9.8167E−04	9.8602E−04	9.8468E−04	9.8553E−04	1.4215E−06	17.672	2
ALSHADE	9.8233E−04	1.2812E−03	1.0201E−03	9.8661E−04	9.3056E−05	81.931	2.5
OBGBO	9.9554E−04	2.0725E−03	1.2711E−03	1.1477E−03	3.4203E−04	10.333	4.2
Case 2—SDM							
RL-GJO	2.2781E−04	2.5400E−02	1.2836E−02	1.2855E−02	1.3243E−02	7.514	2.5
GJO	7.2242E−04	2.5400E−02	1.4418E−02	1.6624E−02	1.1809E−02	7.759	5.6
IMPA	3.9376E−04	6.9210E−04	6.5458E−04	6.8677E−04	9.2075E−05	15.219	3.75
IARO	8.1996E−04	1.1076E−02	6.6942E−03	7.8886E−03	3.6787E−03	18.764	4.2
RLGWO	2.5400E−02	2.5401E−02	2.5400E−02	2.5400E−02	3.2514E−07	7.748	7.7
EJAYA	2.3590E−04	2.5400E−02	1.4105E−02	1.6021E−02	1.2132E−02	13.236	4.2
ALSHADE	2.2781E−04	2.0108E−02	3.4654E−03	3.1897E−04	6.2319E−03	70.723	2.5
OBGBO	4.6623E−04	2.5400E−02	2.2906E−02	2.5400E−02	7.8846E−03	7.056	5.55
Case 2—DDM							
RL-GJO	2.2969E−04	2.5400E−02	1.3239E−02	1.4720E−02	1.2866E−02	6.725	2.7
GJO	7.0489E−04	2.5400E−02	9.9573E−03	6.6433E−03	1.0986E−02	6.375	5.1
IMPA	6.4578E−04	6.9373E−04	6.8136E−04	6.8685E−04	1.5046E−05	13.275	3.75
IARO	1.1152E−03	8.3219E−02	2.2662E−02	8.1890E−03	3.2159E−02	16.809	5.4
RLGWO	1.0885E−03	2.5401E−02	2.2969E−02	2.5400E−02	7.6879E−03	6.916	7.2
EJAYA	2.3688E−04	2.5400E−02	8.4662E−03	6.6425E−03	8.8369E−03	13.163	3.6
ALSHADE	2.7803E−04	3.6111E−02	1.1410E−02	3.2070E−04	1.4674E−02	61.952	3.6
OBGBO	3.8134E−04	2.5400E−02	1.5430E−02	2.5400E−02	1.2870E−02	7.802	4.65
Case 2—TDM							
RL-GJO	1.2991E−04	6.9936E−04	5.8933E−04	6.9936E−04	2.3214E−04	6.981	1.7
GJO	7.0247E−04	2.5400E−02	4.6822E−03	2.3048E−03	7.4881E−03	6.634	6
IMPA	6.7833E−04	2.1886E−03	1.5754E−03	1.7725E−03	5.4809E−04	12.263	4.8
IARO	3.5532E−03	8.3219E−02	1.6268E−02	7.8174E−03	2.4007E−02	14.797	7.6
RLGWO	6.7752E−04	2.5400E−02	6.9265E−03	2.1849E−03	9.7941E−03	7.508	6.2
EJAYA	2.0650E−04	1.0895E−03	3.9367E−04	2.9213E−04	2.6183E−04	12.170	2.4
ALSHADE	1.7704E−04	1.2051E−03	5.0730E−04	3.0309E−04	3.4547E−04	57.103	2.5
OBGBO	1.2525E−03	1.5871E−03	1.4617E−03	1.4603E−03	9.4246E−05	8.580	4.8
Case 3—SDM							
RL-GJO	2.4251E−03	2.4251E−03	2.4251E−03	2.4251E−03	1.3467E−14	8.508	1.2
GJO	2.6192E−03	2.7425E−01	3.7149E−02	2.7217E−03	8.6160E−02	8.136	6.2
IMPA	2.4926E−03	2.6279E−03	2.6065E−03	2.6225E−03	4.1009E−05	16.606	4.9
IARO	9.9783E−03	2.7425E−01	7.6642E−02	4.5790E−02	8.2052E−02	20.033	7.5
RLGWO	2.6087E−03	2.7426E−01	7.2683E−02	1.0082E−02	1.0982E−01	8.909	6.9
EJAYA	2.4251E−03	2.4251E−03	2.4251E−03	2.4251E−03	5.0887E−13	15.698	1.9
ALSHADE	2.4251E−03	1.2370E−01	1.4654E−02	2.4251E−03	3.8314E−02	74.386	4.1
OBGBO	2.4251E−03	2.4251E−03	2.4251E−03	2.4251E−03	1.8754E−08	9.117	3.3
Case 3—DDM							
RL-GJO	2.4251E−03	2.5260E−03	2.4369E−03	2.4251E−03	3.1454E−05	6.117	1.9
GJO	2.7032E−03	7.2956E−02	3.2504E−02	9.8419E−03	3.4924E−02	5.853	6.3
IMPA	2.5763E−03	4.6217E−03	2.8334E−03	2.6264E−03	6.3085E−04	11.627	4.9
IARO	1.0394E−02	4.3627E−01	1.2233E−01	9.5028E−02	1.3399E−01	14.752	7.5
RLGWO	2.4541E−03	2.7425E−01	9.3089E−02	8.9786E−03	1.2674E−01	6.716	6.2
EJAYA	2.4251E−03	2.7425E−01	2.9632E−02	2.4251E−03	8.5950E−02	10.748	2.6
ALSHADE	2.4251E−03	2.7425E−01	3.7582E−02	2.4252E−03	8.6846E−02	53.683	3.4
OBGBO	2.4260E−03	2.5442E−03	2.4797E−03	2.4856E−03	4.7485E−05	8.427	3.2
Case 3—TDM							
RL-GJO	2.4251E−03	2.6078E−03	2.4723E−03	2.4302E−03	7.2319E−05	6.416	1.8
GJO	2.6315E−03	7.2958E−02	3.0877E−02	3.0722E−03	3.6216E−02	5.588	5.7
IMPA	2.5841E−03	2.6816E−03	2.6263E−03	2.6219E−03	2.8726E−05	12.070	4.3
IARO	6.0361E−02	1.0548E+00	4.0558E−01	2.7428E−01	3.4856E−01	14.884	7.7
RLGWO	2.6258E−03	2.7425E−01	1.0257E−01	4.2205E−02	1.2225E−01	6.866	6.4
EJAYA	2.4251E−03	2.7425E−01	8.3995E−02	2.4433E−03	1.3129E−01	11.742	3.2
ALSHADE	2.4251E−03	2.7425E−01	5.7665E−02	2.4598E−03	1.1417E−01	53.211	3.9
OBGBO	2.4457E−03	2.5592E−03	2.5030E−03	2.4983E−03	4.0747E−05	7.589	3
Case 4—different irradiation condition							
RL-GJO	7.7645E−04	1.2046E−03	9.9052E−04	9.9052E−04	3.0274E−04	8.750	1.00
GJO	1.7988E−02	3.1290E−02	2.4639E−02	2.4639E−02	9.4061E−03	8.259	6.60
IMPA	1.1120E−02	1.6632E−02	1.3876E−02	1.3876E−02	3.8976E−03	17.017	5.40
IARO	6.5791E−02	1.4824E−01	1.0701E−01	1.0701E−01	5.8298E−02	21.152	7.70
RLGWO	1.7044E−02	3.3407E−02	2.5226E−02	2.5226E−02	1.1570E−02	9.420	6.30
EJAYA	1.1905E−03	2.4845E−03	1.8375E−03	1.8375E−03	9.1506E−04	14.163	2.50
ALSHADE	2.7430E−03	3.1981E−03	2.9706E−03	2.9706E−03	3.2179E−04	78.617	2.50
OBGBO	6.1327E−03	8.5497E−03	7.3412E−03	7.3412E−03	1.7090E−03	10.659	4.00
Case 4—different temperature condition							
RL-GJO	2.9051E−03	3.2244E−03	3.0648E−03	3.0648E−03	2.2576E−04	9.175	1.17
GJO	3.8294E−02	4.7630E−02	4.2962E−02	4.2962E−02	6.6015E−03	8.846	6.83
IMPA	1.9557E−02	2.6506E−02	2.3031E−02	2.3031E−02	4.9132E−03	17.448	5.33
IARO	1.1801E−01	2.1427E−01	1.6614E−01	1.6614E−01	6.8067E−02	21.094	8.00
RLGWO	1.9154E−02	3.0415E−02	2.4785E−02	2.4785E−02	7.9627E−03	9.888	5.83
EJAYA	3.8155E−03	3.9727E−03	3.8941E−03	3.8941E−03	1.1118E−04	15.599	2.33
ALSHADE	3.9130E−03	4.8685E−03	4.3907E−03	4.3907E−03	6.7563E−04	77.935	2.50
OBGBO	9.3072E−03	1.1319E−02	1.0313E−02	1.0313E−02	1.4223E−03	10.195	4.00

Among all algorithms, rankings are provided based on the ‘Min’ and ‘STD’ values using FRT. In this context, the RLGJO stands out, producing the lowest RMSE value, which is a measure of the differences between the predicted and observed values. This suggests that the RLGJO is the most accurate among the tested algorithms. The subsequent rankings, based on this criterion, are EJAYA, ALSHADE, OBGBO, IMPA, GJO, RL-GWO, and IARO. Reliability refers to consistency in results. An algorithm with a lower STD value is considered more reliable because its performance doesn’t fluctuate as much. Table 29 demonstrates that the RL-GJO claims superior reliability, evident from its lowest STD value, as compared to other algorithms for all PV models. While the RL-GJO’s run time is marginally higher than the basic GJO, it still surpasses the other algorithms in terms of efficiency.FRT has been employed for a holistic ranking of all algorithms. As per FRT, the sequence of performance is as follows: RL-GJO is the top performer with an average FRT of 1.74, followed by EJAYA (average FRT is 2.73), ALSHADE (average FRT is 2.95), OBGBO (average FRT is 3.96), IMPA (average FRT is 4.79), GJO (average FRT is 6.4), RLGWO (average FRT is 6.59), and IARO (average FRT is 6.87) for all the tested cases. Based on the intricate analysis presented, it is evident that the proposed RL-GJO algorithm is exceptional in its performance, outperforming all selected algorithms in the majority of the case studies. Whether its accuracy, reliability, or efficiency, the RL-GJO consistently shines, establishing itself as a superior algorithm in this domain.

## Conclusions

In this research, an improved version of the GJO is presented. Named RL-GJO, this enhanced algorithm integrates the principles of reinforced learning and innovative non-linear hunting strategies. The primary objective is to precisely identify unknown parameters within various PV models, both at the cell and module levels. At the heart of RL-GJO lies the incorporation of a q-learning mechanism. This addition infuses the algorithm with the ability to learn and adapt from its past experiences, subsequently improving its future decisions. Another distinct aspect of the RL-GJO is its novel hunting approach. Unlike traditional linear methods, this non-linear strategy is geared towards refining both the exploration and exploitation capabilities of the algorithm. To discover the effectiveness of RL-GJO, its performance was benchmarked against various state-of-the-art algorithms in the realm of PV parameter estimation. The findings were clear: RL-GJO outperformed its competitors, showcasing a high proficiency in handling complicated PV parameter estimation challenges. The reliability of RL-GJO was evaluated through metrics such as RMSE, RE, AE, R^2^, and MBE. These metrics measure the deviations between the algorithm’s predictions and actual observations. The statistics like Min, Max, Mean, Median, and RT provided a holistic overview of RL-GJO’s performance across different test scenarios. The convergence curve visualizes the algorithm’s progression towards an optimal solution over time. A robust FRT was employed to rank the tested algorithms based on their performances. In this regard, RL-GJO emerged as a top performer, boasting an impressive average rank value of 1.742, thereby securing the first position among all the evaluated algorithms. Beyond numerical metrics, the RL-GJO’s ability was also validated visually using I–V and P–V curves. These curves depict the relationship between current, voltage, power, and voltage for PV cells/modules. By comparing the experimental and RL-GJO estimated data points on these curves, a compelling narrative emerged. The I–V and P–V curves revealed an impeccable alignment between the real-world and predicted data points, highlighting the accuracy. Upon synthesizing all the gathered insights, one can confidently conclude that the RL-GJO is not just another algorithm in the vast landscape of PV parameter estimation tools. Instead, it stands as a model of precision, robustness, and innovation, rendering it a crucial asset for accurately estimating or discerning unknown parameters within solar PV cells and modules.

Future extensions of the RL-GJO algorithm for PV parameter estimation include hybridizing it with other optimization techniques to enhance performance. Broadening its scope to diverse PV systems, such as bifacial modules or integrating with energy storage, can open new applications. It is essential to test RL-GJO in dynamic real-world environments, and transforming from simulations to real-world installations would provide a complete performance view. Producing user-friendly software platforms can democratize access while introducing the algorithm to interdisciplinary studies, like climate modelling or economic analysis, can maximize its impact. Lastly, continuously updating and learning from larger, diverse datasets ensures the algorithm’s robustness and adaptability.

## Data Availability

The datasets used during the current study are available from the corresponding author upon reasonable request.
